# Optimization and validation of multiresidual extraction methods for pharmaceuticals in Soil, Lettuce, and Earthworms

**DOI:** 10.1007/s11356-024-33492-7

**Published:** 2024-04-27

**Authors:** Ludmila Mravcová, Anna Amrichová, Jitka Navrkalová, Marie Hamplová, Marian Sedlář, Helena Zlámalová Gargošová, Jan Fučík

**Affiliations:** 1 Institute of Chemistry and Technology of Environmental Protection, Faculty of Chemistry, Brno University of Technology, Purkyňova 118, 612 00 Brno, Czech Republic; 2 CEITEC Brno University of Technology, Purkyňova 656/123, 612 00 Brno, Czech Republic

**Keywords:** Pharmaceutical pollution, Multiresidual analytical methods, Solid-phase extraction, QuEChERS, Liquid chromatography, Mass spectrometry

## Abstract

The presence of human and veterinary pharmaceuticals (PhACs) in the environment poses potential risks. To comprehensively assess these risks, robust multiresidual analytical methods are essential for determining a broad spectrum of PhAC classes in various environmental compartments (soil, plants, and soil organisms). This study optimized extraction methods for analyzing over 40 PhACs from various matrices, including soil, lettuce, and earthworms. A four-step ultrasonic extraction method with varying extraction conditions and subsequent solid phase extraction was developed for soil samples. QuEChERS methods were optimized for extracting PhACs from lettuce and earthworm samples, addressing a literature gap in these less-studied matrices. The quantification of PhACs in soil, lettuce, and earthworm extracts was performed using a single LC–MS/MS method. Following thorough method validation, earthworms and lettuce were exposed to a mixture of 27 pharmaceuticals in a soil environment. The method validation results demonstrated the robustness of these methods for a broad spectrum of PhACs. Specifically, 29 out of 42 PhACs were extracted with an average efficiency > 50% and RSD < 30% from the soil; 40 out of 42 PhACs exhibited average efficiency > 50% and %RSD < 30% from the earthworms, while 39 out of 42 PhACs showed average efficiency > 50% and RSD < 30% from the lettuce. Exposure experiments confirmed the viability of these methods for quantifying a diverse range of PhACs in different environmental compartments. This study presents three thoroughly validated methods for determining more than 40 PhACs in diverse matrices, enabling a comprehensive assessment of PhAC dissemination in the environment.

**Graphical Abstract:**

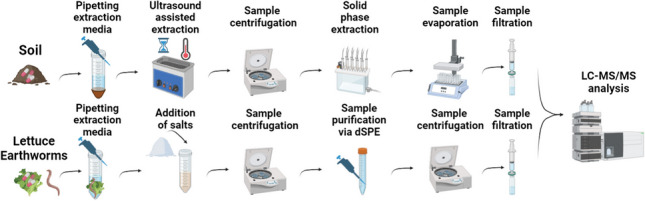

**Supplementary Information:**

The online version contains supplementary material available at 10.1007/s11356-024-33492-7.

## Introduction

The widespread use of pharmaceuticals (PhACs) is crucial for the effective treatment of diseases in both humans and animals (Cycoń et al. 2019; Rakonjac et al. 2022). Nevertheless, owing to pharmacokinetic variables, specific transformation processes, and the nature of the pharmaceutical class, a substantial portion—ranging from 30 to 90%—of the administered drug dosage may be excreted either unchanged or in a metabolized form, predominantly through feces or urine (Pan and Chu 2017; Solliec et al. 2016). Some drug residues are not fully removed by conventional treatment methods employed in wastewater treatment plants (WWTPs), potentially leading to their presence in both WWTP effluent and treated sewage sludge. A study by Zhang et al. (2017) investigated pharmaceutical removal at different WWTPs, revealing varying rates due to distinct treatment methods and operational factors. Sulfonamides showed -97.9% to 100%, tetracyclines 0–100%, macrolides -189.9–97.4%, and fluoroquinolones -42.2–100% removal. Another study by Gaffney et al. (2017) reported over 90% removal efficiency for nonsteroidal anti-inflammatory drugs, antidiabetics, and psychostimulants. These residues can subsequently enter the soil through wastewater irrigation (wastewater recycling), sewage sludge application, or the application of animal waste (such as manure or slurry) to agricultural land (Ajibola et al. 2020; Riva et al. 2021). In Europe, recycling practices into agricultural land are a widely promoted practice within the circular economy, as it is considered to be a potential substitute for mineral fertilizers (Riva et al. 2021).

The presence of human and veterinary pharmaceuticals in the environment has already been recognised as a potential environmental threat (Gao et al. 2020). The concentrations of PhACs in soil matrices exhibit a wide range, spanning from a few ng·g^−1^ to μg·g^−1^ of soil (Cycoń et al. 2019; Pan and Chu 2017). According to Table S1, these soil concentrations correspond to varying levels of risk for the emergence of antimicrobial resistance, ranging from none to high, depending on the specific antimicrobial agent and soil properties. Significantly elevated concentrations are commonly observed in agricultural fields where soil enrichment with manure (PhACs concentrations ranging from ng·g^−1^ to mg·g^−1^), wastewater (ng·L^−1^ to mg·L^−1^), or biosolids (ng·g^−1^ to μg·g^−1^) occurs (Cycoń et al. 2019; Pan and Chu 2017). Furthermore, these pharmaceuticals may be up-taken by plants (concentrations reported from ng·g^−1^ to μg·g^−1^ (Kodešová et al. 2019) or soil organisms (concentrations reported from ng·g^−1^ to μg·g^−1^ (Carter et al. 2014; Kinney et al. 2008), raising concerns about potential human health risks associated with prolonged dietary intake of these micropollutants (Gros et al. 2019). Furthermore, pharmaceuticals may undergo transformation into degradation products or metabolites within terrestrial environments, plants, or soil organisms. Previous studies have explored metabolites of NSAIDs (Emhofer et al. 2017), macrolides (Tian et al. 2019), sulfonamides (Tadić et al. 2022; Tian et al. 2019), fluoroquinolones (Maia et al. 2014), as well as tetracyclines (Xu et al. 2019). Despite the dispersion of some parent pharmaceuticals and their degradation products and metabolites into the environment over an extended period, their presence and toxicity remain to be definitively established, with no regulatory frameworks currently in place (Souza et al. 2018). Although, published findings indicate various adverse effects, including seed germination and crop growth inhibition, suppression of microbial and soil enzymatic activity, PhAC accumulation in crop biomass and soil invertebrates, and the emergence of antimicrobial resistance (Pereira et al. 2015; Pino et al. 2016).

Hence, a pressing demand exists for robust, dependable and detailed methodologies capable of quantifying trace concentrations of pharmaceuticals in various environmental compartments such as soil, plants, and soil organisms. This necessity arises from the imperative to comprehensively evaluate the potential impact of these micropollutants on both the environment and human health (Caldú and Diaz-Cruz 2021; Bergé and Vulliet 2015; Bielińska et al. 2016; Yu et al. 2017). These analytical methods play crucial roles in 1) Identifying contamination hotspots (Beek et al. 2016; Duarte et al. 2018), 2) Evaluating compliance with future pollutant limits standards (Duarte et al. 2018), 3) Identifying priority pharmaceuticals for focused monitoring and regulatory action in response to potential risks (Celiz et al. 2009; Safont et al. 2023), 4) Assessing the efficacy of removal technologies (advanced oxidation processes, phytoremediation, etc.) (Bair et al. 2016; Carvalho 2020; Celiz et al. 2009; Hernandez 2020; Ho et al. 2013; Li et al. 2020; Safont et al. 2023; Zhang et al. 2015) and 5) Informed fertilizer decision-making considering drug residue contamination levels (Bartrons and Peñuelas 2017).

Sample preparation plays a crucial role in the analysis of pharmaceuticals in environmental or biological samples. Analytes are frequently present at trace levels, and their quantification is often complicated by matrix interferences (Albero et al. 2019; Hu et al. 2012). Despite the availability of various extraction methods for quantifying pharmaceutical residues in different matrices, the analysis scope typically encompasses only a limited number of pharmaceuticals (< 25), leaving some pharmaceutical groups underrepresented. While numerous extraction methods have been devised for wastewater and soil, biological matrices such as plants, and especially soil invertebrates, have garnered limited research attention (Kim et al. 2019; Llompart et al. 2019; Manasfi et al. 2020). Moreover, the recovery rates reported in environmental studies of PhACs display significant variations, reflecting diverse aims and methodological approaches. This often leads to brief descriptions of extraction procedures, that lack comprehensive details on optimization and validation steps, thereby complicating reproducibility in other laboratories. Additionally, in the case of soil samples, the physicochemical properties of the soil are rarely or only partially reported, making it impossible to compare recovery rates due to the effects of soil properties on extraction efficiency (Ajibola et al. 2020; Carvalho et al. 2018; Golovko et al. 2016; Huang et al. 2018; Mullen et al. 2017; Rodrigues et al. 2023). A short review of existing, appropriately validated methods, including their recovery rates (RR%) in the literature, is presented for soil (Table S2), lettuce (Table S3), and earthworms (Table S4).

Hence, in the present work, three multiresidual extraction methods (soil, lettuce, and earthworms) have been developed for over 40 different pharmaceuticals, representing various pharmaceuticals groups (beta blockers, tetracyclines (TCs), sulfonamides (SAs), macrolides (MLs), fluoroquinolones (FQs), nonsteroidal anti-inflammatory drugs (NSAIDs), topical products for joint and muscular pain, throat preparations, and antiepileptics). The selection of pharmaceutical substances in the study followed a prioritization process, taking into account factors such as high prevalence, potential environmental impact (e.g., if they belong to critically important antimicrobials; Table S1), reported sales within the EU (EMA 2023), and findings from other environmental and monitoring studies (Ajibola et al. 2020; Carter et al. 2014; Cycoń et al. 2019; Gaffney et al. 2017; Gros et al. 2019; Kinney et al. 2008; Kodešová et al. 2019; Pan and Chu 2017; Riva et al. 2021; Souza et al. 2018). The aim of this study was to assemble a representative set of pharmaceuticals that span diverse therapeutic classes, ensuring a comprehensive assessment of their occurrence and fate in different matrices. This study have introduced a novel extraction method for PhACs from soil using a four-step ultrasound-assisted extraction method with varying extraction parameters, including solvent composition, extraction mechanism, and temperature. Subsequently, solid-phase extraction (SPE) was employed for the cleanup of soil extracts. Additionally, QuEChERS methods were developed for extracting PhACs from lettuce and earthworm samples, addressing the lack of appropriate investigation in the existing literature for these biological matrices. In contrast to soil, QuEChERS methods were chosen for lettuce and earthworm samples due to less complicated matrices and the advantages of simplicity, rapidity, and low solvent consumption offered by QuEChERS. For quantification, a single LC–MS/MS method was optimized and employed to quantify PhACs in all three matrices. These analytical methods were rigorously validated by assessing various parameters, including calibration range, detection limit, quantification limit, method recovery rate, matrix effect, intra-day precision, inter-day precision, selectivity/specificity, and identity confirmation. Given the robustness of these methods, they are transferable to other laboratories. Furthermore, revalidating for an extended array of pharmaceuticals should be uncomplicated, given that these methods were fine-tuned to cater to a broad spectrum of pharmaceutical classes.

## Experimental

### Reagents and standards

Ethylenediamine tetraacetic acid (EDTA, ≥ 99%), citric acid monohydrate (≥ 99%), disodium hydrogen phosphate dodecahydrate (≥ 99%) and sodium sulfate anhydrous (≥ 95%), potassium dihydrogen phosphate (≥ 99%) and hydrochloric acid (35%) were purchased from Lach:ner (Czech Republic). Magnesium nitrate hexahydrate (> 99%), ammonium (25%), methanol (LC–MS grade), acetonitrile (LC–MS grade) and water (LC–MS grade) were purchased from VWR (USA). Sodium hydroxide (> 98%) and phosphoric acid (85%) were purchased from Penta Chemicals (Czech Republic). Formic acid (LC–MS grade) and sodium chloride (> 99%) were purchased from Sigma Aldrich (Germany).

The following pharmaceuticals (see their properties in Table S5) were used: acebutolol hydrochloride (≥ 99%), doxycycline hyclate (≥ 95%), naproxen (≥ 99%), oxytetracycline hydrochloride (≥ 94%), sulfacetamide (≥ 98%) and sulfasalazine (≥ 97%) were purchased from Honeywell (USA). Meloxicam (≥ 97%), ofloxacin (≥ 98%), and sulfamethoxypyridazine (≥ 97%) were purchased from Thermo Fisher Scientific (USA). Atenolol (≥ 98%), azithromycin (≥ 98%), carbamazepine (≥ 99%), celecoxib (≥ 98%), chlortetracycline hydrochloride (≥ 91%), ciprofloxacin (≥ 98%), clarithromycin (≥ 97%), diclofenac sodium salt (≥ 98%), enrofloxacin (≥ 99%), erythromycin (≥ 97%), flurbiprofen (≥ 98%), indomethacin (≥ 98%), ketoprofen (≥ 98%), mefenamic acid (≥ 98%), minocycline (≥ 98%), moxifloxacin (≥ 96%), nadolol (≥ 98%), nimesulide (≥ 98%), norfloxacin (≥ 98%), pefloxacin mesylate dihydrate (≥ 97%), pindolol (≥ 98%), piroxicam (≥ 98%), propranolol hydrochloride (≥ 98%), roxithromycin (≥ 95%), sulfadiazine (≥ 99%), sulfadimethoxine (≥ 98%), sulfamerazine (≥ 99%), sulfamethazine (≥ 99%), sulfamethoxazole (≥ 98%), sulfapyridine (≥ 99%), sulfathiazole (≥ 99%), tetracycline (≥ 98%) and trimethoprim (≥ 98%) were purchased from Sigma Aldrich (Germany). The following substances were used as internal standards (IS): atenolol-d7 (≥ 97%), ciprofloxacin-d8 (≥ 99%), enrofloxacin-d5 (≥ 99%), spiramycin (≥ 90%) and trimethoprim-d9 (≥ 97%) were purchased from Sigma Aldrich (Germany). Sulfamethoxazole-d4 was purchased from Neochema GmbH (Germany). Sulfathiazole-d4 was purchased from Toronto Research Chemicals (Canada).

Individual stock standard solutions were prepared in methanol at a concentration of 1 mg·mL^−1^ for the target pharmaceuticals. A standard solution mixture (concentration of 10 μg·mL^−1^) containing all the target compounds was then prepared in methanol. Working standard solutions were obtained by further diluting the standard solution mixture. All standard solutions were stored in glass vials and kept at -20 °C in a freezer. Additionally, the preparation of the buffer solution for the extraction media can be found in the supplementary information, specifically regarding the Phosphate buffer and McIlvaine buffers.

Nitrogen gas (4.7) and argon gas (5.0) were purchased from SIAD Czech spol. s.r.o. (Czech Republic). Nylon syringe filters (13 mm, 0.22 μm) and solid phase extraction (SPE) HLB cartridges (200 mg/6 mL, particle diameter 25–35 μm) were purchased from Chromservis (Czech Republic). For QuEChERS, dispersive SPE (dSPE): DSC-18 SPE, PSA SPE, and ENVI-CARB SPE (Graphitized Carbon black, GCB) were purchased from Sigma Aldrich (Germany).

### Extraction methods

Three distinct extraction methods, systematically optimized and validated, were developed for the determination of pharmaceuticals in diverse matrices, including both environmental (soil) and biological (lettuce and earthworm) samples. Following the optimization, a comprehensive validation process was undertaken by inspecting the following parameters: calibration range, limit of detection, limit of quantification, method recovery rate, matrix effect, intra-day precision, inter-day precision, selectivity/specificity, and identity confirmation.

#### Extraction of pharmaceuticals from soil

The soil was obtained from an experimental field located in Brno (Czech Republic). The physicochemical analysis of soil properties was conducted (Table S6) due to their crucial influence on the fate of pharmaceuticals within the soil environment. Additionally, these properties play a significant role in determining the extraction efficiency and matrix effect, as indicated by a previous study from Gworek et al. (2021). During method development, air-dried soil was consistently spiked with a known concentration of PhACs and allowed to evaporate overnight to assess recovery rates. The optimization of the method involved various parameters, including the composition of the extraction medium (utilizing different organic solvents, adjusting the pH of the aqueous phase, and use of MgNO_3_), determining the optimal amount of EDTA, and optimizing the extraction temperature. The subsequent procedure was devised for the extraction of PhACs from soil samples (extraction scheme in Fig. S1).

In the initial step of sample preparation, 1 g of soil was precisely weighed and placed into a 50 mL polyethylene (PE) centrifugation tube. The extraction procedure (Steps 1–2) involved pipetting 5 mL of methanol (MeOH) and 5 mL of phosphate buffer (pH 3) into the sample, followed by vortexing for 30 s. Subsequently, PhACs were extracted using an ultrasound bath for 10 min at 12 °C. After sonication, the mixture was centrifuged at 4,800 rpm for 8 min at 20 °C. The resulting supernatant was transferred into a 30-mL dark glass vial. The extraction process was then repeated using the same extraction medium and extraction conditions. The extracts obtained from extraction rounds (1–2) were combined and placed in the same vial. In the subsequent extraction steps (3–4), 0.6 g of EDTA was added to the soil along with 7.5 mL of acetonitrile (ACN), 7.5 mL of McIlvaine buffer (pH 8), 4.8 mL of Mg(NO_3_)_2_·6H_2_O aqueous solution (concentration 0.5 g·mL^−1^), and 0.2 mL of 2.5% NH_3_ aqueous solution in the centrifugation tube. The mixture was vortexed for 30 s, followed by PhAC extraction using an ultrasound bath for 10 min at 35 °C. After sonication, the solution was centrifuged at 4,800 rpm for 8 min at 20 °C. Subsequently, the supernatant was transferred to a dark 30 mL glass vial. The extraction process was repeated with half the volume of the extraction medium (EM) without the addition of EDTA (3.75 mL of McIlvaine buffer, 3.75 mL of ACN, 2.4 mL of Mg(NO_3_)_2_ solution, and 0.1 mL of 2.5% NH_3_) under the same extraction conditions. The extracts obtained from extraction rounds (3–4) were combined and placed in the same vial. During the pre-concentration step, both vials were subjected to evaporation under a nitrogen stream in a thermostatic metal block heated to 40 °C until each vial’s weight loss reached 6 g. Subsequently, the contents of both vials were combined and transferred into a 600-mL beaker, and the soil extract was diluted by adding 480-mL Milli-Q water to decrease the percentage of the organic phase (no additional pH adjustment was made). In the solid phase extraction step, the pre-concentrated soil extracts were purified using Chromservis HLB cartridges (200 mg; 6 mL; Particle diameter 25–35 μm; Chromservis Czech Republic) using a Baker vacuum system (J.T. Baker, Deventer, The Netherlands). To outline the procedure briefly, the SPE column was conditioned with 6 mL of MeOH, followed by 6 mL of Milli-Q water, with a flow rate of approximately 1 mL∙min^−1^. Subsequently, the diluted soil extract was loaded onto the column at a flow rate of 5 mL∙min^−1^.The washing step was performed with 15 mL of Milli-Q water at a flow rate of 1 mL·min^−1^, followed by 2 min of vacuum drying of the sorbents. Finally, the elution of PhACs was achieved by passing 9 mL of 0.1% formic acid (FA) in MeOH, and the eluate was collected into 20 mL glass vials. This was followed by evaporation under the nitrogen stream in the thermostatic metal block heated to 40 °C to dryness. Subsequently, 5 μL of an internal standards mixture (concentration of mixture 10 μg∙mL^−1^) was introduced, followed by the addition of 995 μL of 0.1% FA in H_2_O:ACN (95:5, v/v). The sample was filtered through 0.22 μm nylon syringe filters (diameter 13 mm) into a 2-mL glass vial. The prepared sample was then subjected to LC–MS/MS analysis.

#### Extraction of pharmaceuticals from lettuce leaves

Lettuce seeds (*Lactuca sativa* – Australischer Gelber) were purchased from FloraSelf in the Czech Republic and subsequently cultivated under hydroponic conditions to generate blank lettuce samples. During method development, freeze-dried lettuce samples were consistently spiked with known concentrations of PhACs and left to evaporate overnight to assess recovery rates. The following parameters were optimized during method development: the composition of the extraction medium (varied organic solvents and pH of the aqueous phase), the quantity of EDTA, and the composition of the dSPE. A subsequent procedure was devised for the extraction of PhACs from lettuce samples (QuEChERS extraction scheme in Fig. S2).

In the initial step of sample preparation, 0.1 g of lyophilized and homogenized lettuce leaves were accurately weighed and placed into a 50 mL PE centrifugation tube. In the extraction step, ceramic homogenizers were initially introduced, followed by pipetting of 5 mL of the extraction medium (MeOH:McIlvaine buffer, pH 2.6:ACN in a ratio of 8:20:72). Following this, the sample was vortexed for 1 min. Subsequently, separation salts (2 g anhydrous Na_2_SO_4_ and 0.5 g NaCl) were added, and the mixture was further vortexed for 1.5 min before centrifugation at 3,500 rpm for 10 min at 20 °C. In the purification step, following centrifugation, 2 mL of the organic phase was carefully pipetted into a 15 mL PE centrifugation tube preloaded with dSPE sorbents (12.5 mg DSC-18, 12.5 mg PSA, and 225 mg of anhydrous Na_2_SO_4_). Subsequently, the sample was vortexed for 1 min, followed by another centrifugation at 3,500 rpm for 10 min at 20 °C. Finally, the sample was filtered through 0.22 μm nylon syringe filters (diameter 13 mm) into a 2-mL glass vial, ready for LC–MS/MS analysis.

#### Extraction of pharmaceuticals from earthworms

Earthworms (*Eisenia fetida*) were purchased from a local fish store (ProRyby, Czech Republic). Before PhAC extraction, all samples were lyophilized. During method development, freeze-dried earthworm samples were consistently spiked with known concentrations of PhACs and left overnight to evaporate, facilitating the determination of recovery rates. The following parameters were optimized during method development: the composition of the extraction medium (varied organic solvents and pH of the aqueous phase), the quantity of EDTA, and the composition of dSPE. A subsequent procedure was devised for the extraction of PhACs from earthworm samples (QuEChERS extraction scheme in Fig. S3).

In the initial step of sample preparation, 0.1 g of lyophilized and homogenized earthworms was accurately weighed and placed into a 50 mL PE centrifugation tube. In the extraction step, ceramic homogenizers were initially introduced, followed the addition of 10 mg EDTA and by pipetting of 5 mL of the extraction medium (MeOH:McIlvaine buffer pH 2.6 in a ratio of 80:20). Following this, the sample was vortexed for 1 min. Subsequently, separation salts (2 g anhydrous Na_2_SO_4_ and 0.5 g NaCl) were added, followed by vortexing for 1.5 min and centrifugation (3,500 rpm) for 10 min at 20 °C. In the purification step, after centrifugation, 2 mL of the organic phase was carefully pipetted into a 15 mL PE centrifugation tube preloaded with dSPE sorbents (12.5 mg DSC-18, 12.5 mg PSA, and 225 mg of anhydrous Na_2_SO_4_). The sample was vortexed for 1 min and centrifuged (3,500 rpm) for an additional 10 min at 20 °C. Finally, the sample was filtered through 0.22 μm nylon syringe filters (diameter 13 mm) into a 2-mL glass vial, ready for LC–MS/MS analysis.

### LC–MS/MS analysis

Instrumental analysis for the quantification of PhACs in all extracts (soil, lettuce, and earthworms) was performed using ultra-performance liquid chromatography (UHPLC Agilent 1290 Infinity LC) coupled with a triple quadrupole mass spectrometer (Bruker EVOQ LC-TQ) with electrospray ionization (ESI). The gas sources of nitrogen and air were provided by an external gas generator (Peak Scientific – Genius 3045).

Chromatographic separation was accomplished using a Luna® Omega Polar C18 Phenomenex column (100 × 2.1 mm, 1.6 µm). The column temperature was optimized at 35 °C, and the flow rate was set to 0.5 mL·min^−1^. The mobile phases consisted of A) 0.1% FA in H_2_O and B) ACN, following a gradient program for the A eluent (%): t(0 min) = 90, t(0.5 min) = 90, t(13.0 min) = 35, t(14.0 min) = 10, and t(15.5 min) = 90. The LC method was set to a stop time of 16 min, with a 2-min re-equilibration time. The injection volume for all analyses was 7 µL. To prevent carry-over, an external needle wash was performed using a wash solvent composed of FA:H_2_O:ACN at a ratio of 1:9:90 for 30 s.

The MS conditions were set as follows for electrospray ionization in positive mode: spray voltage: 4,500 V; cone temperature: 350 °C; cone gas flow: 15 arbitrary units (a.u.); heated probe temperature: 500 °C; probe gas flow: 25 a.u.; nebulizer gas flow: 45 a.u.; and exhaust gas: ON. For both quantitative and qualitative analysis of PhACs, the multiple reaction monitoring (MRM) mode was employed, using the specific MRM transitions outlined in Table S7. Argon served as the collision gas at a pressure of 1.5 mTorr.

### Method validation

All three extraction methods, including sample preparation through ultrasound-assisted extraction and solid-phase extraction for soil samples and QuEChERS for lettuce and earthworm samples, were validated. The Method Limits of Detection (MLoD) and Limits of Quantification (MLoQ) were recalculated from instrumental limits using Eqs. 1–2, considering sample preparation and recovery rates. Matrix effects were compensated for using internal standards for soil samples and matrix-matched calibration for lettuce and earthworm samples. Instrumental Limits of Detection (ILoD) and Limits of Quantification (ILoQ) were determined using a method based on the standard deviation of the blank extract response and calibration slope. Linearity was considered validated when the determination coefficient (R^2^) exceeded 0.995 within the working concentration range. Recovery rates (RR%) were determined by spiking blank samples (soil, lettuce, or earthworms) at different concentrations, and Eq. 3 was employed for the calculation. Matrix effects (% ME) were determined using Eq. 4, which represents the ratio of the analyte signal in the post-extraction spiked blank matrix to the analyte signal in the solvent. Trueness was expressed as RR%, and intra-day and inter-day precisions were calculated in terms of the relative standard deviation (RSD%) of RR (Eq. 5). The selectivity/specificity of the method was evaluated by analyzing blank samples. Confirmation of identity was achieved by monitoring the ion ratio of multiple MRM transitions during LC–MS/MS analysis.

1 $${\text{MLoD}}={\text{ILoD}}\cdot \frac{100}{\mathrm{RR\%}}=3\cdot \frac{\upsigma }{{\text{S}}}\cdot \frac{100}{\mathrm{RR\%}}$$

where ILoD stands for Instrumental Limit of Detection, RR% is recovery rate at the lowest tested concentration level*, σ* stands for the standard deviation of the blank extract response, and *S* is the slope of the calibration curve.

2 $$M{\text{LoQ}}={\text{ILoQ}}\cdot \frac{100}{\mathrm{RR\%}}=9\cdot \frac{\upsigma }{{\text{S}}}\cdot \frac{100}{\mathrm{RR\%}}$$

where ILoQ stands for Instrumental Limit of Quantification, RR% is recovery rate at the lowest tested concentration level*, σ* stands for the standard deviation of the blank extract response, and *S* is the slope of the calibration curve.

3 $$\mathrm{Recovery\;rate }\left(\mathrm{\%\;RR}\right)=\frac{\mathrm{Measured\;Analyte\;concentration}}{\mathrm{Spiked\;Analyte\;concentration}}\cdot 100$$

4 $$\mathrm{Matrix\;effect\;}(\mathrm{\% ME})=\frac{{\mathrm{Analyte\;signal}}_{{\text{post}}-\mathrm{extraction\;spiked\;matrix}}}{{\mathrm{Analyte\;signal}}_{{\text{solvent}}}}\cdot 100$$

5 $$\mathrm{RSD \%}=\frac{\mathrm{Standard\;deviation\;of\;RR\%}}{\mathrm{Mean\;RR\%}}\cdot 100$$

### Exposure experiments

Following the development and validation of the extraction methods, exposure experiments were conducted using lettuce and earthworms in a contaminated soil environment containing a mixture of 27 PhACs at a concentration of 1,000 ng·g^−1^. A detailed and comprehensive description of these uptake experiments, including the list of utilized pharmaceuticals, is provided in the Supplementary Information.

## Results and discussion

### Optimization of extraction procedures for soil samples

During method development, air-dried soil was spiked with known concentrations of PhACs, and the following parameters were optimized: composition of the extraction medium (different organic solvents, pH of aqueous phase, presence of MgNO_3_), amount of EDTA, and extraction temperature. These parameters were changed to shift equilibrium, therefore increasing the recovery rates of extraction, as the following physicochemical soil properties influence recovery yields: pH, ionic strength, organic matter content (amount and quality of organic matter), type of divalent cations present, type of minerals present, application of organic waste, and weather conditions (Hu et al. 2019; Kuppusamy et al. 2018; Pan and Chu 2017).

#### Influence of extraction media composition

When simultaneously extracting various groups of PhACs, a challenge arises due to the broad spectrum of their properties, such as pKa and log_Kow_. The optimal extraction conditions for these substances can vary significantly. Consequently, multiple combinations, as detailed in Table S8, were explored, encompassing different organic solvents (methanol vs acetonitrile), varied pH levels (2.6, 4.0, 6.0, and 8.0), and distinct extraction mechanisms (including MgNO_3_-mediated extraction via metal complexation) as can be seen in Fig. S4. The selection of extraction media (EM) and extraction parameters drew inspiration from previous studies (Hang et al. 2021; Huang et al. 2013; Silva et al. 2020; Tetzner et al. 2016; Xu et al. 2021; Yu et al. 2012), considering the complexity of the substances being targeted.

##### Influence of organic solvent

The impact of organic solvents on extraction efficiency was explored in this study, employing ACN and MeOH in extraction experiments (5 mL of organic solvent + 5 mL of Milli-Q water; Fig. S5). ACN, characterized by higher elution strength and lower polarity compared to MeOH (Ferhi et al. 2016), exhibited differential extraction selectivity due to distinct functional groups. Notably, FQs displayed a higher RR in the presence of acetonitrile/water solvent, achieving a 19% RR with ACN versus 6% with MeOH. Similarly, MLs showed a higher RR in the presence of ACN 19% versus 3% with MeOH. In contrast, SAs exhibited higher extraction efficiencies with methanol/water, showcasing 49% RR with ACN versus 75% with MeOH, attributed to the attraction of NH_2_ on the aromatic ring to MeOH. However, the influence of organic solvent on other PhAC groups was not observed, attributed to varying functional groups. This solvent-specific effect for SAs, MLs and FQs was particularly evident when Milli-Q water was employed without pH adjustment. This aligns with existing literature highlighting the substantial impact of pH on RR (Carballo et al. 2007; Ferhi et al. 2016; Golovko et al. 2016), which is influenced by the differing pKa values of PhACs and soil composition. Additionally, a previous study by Chitescu et al. (2012) suggested that ACN/H_2_O is more suitable for sandy soil samples, whereas MeOH/H_2_O is preferable for clay soil samples. The ratio of organic solvent (e.g., ACN, MeOH) to water has also been investigated in several studies by Ferhi et al. (2016) and Vazquez-Roig et al. (2010). Generally, a higher ratio of the water phase resulted in increased RR for more hydrophilic substances, whereas a higher ratio of organic solvent led to elevated RR for hydrophobic substances (Ferhi et al. 2016). Despite variations, the commonly adopted ratio of water to organic solvent is approximately 0.5 (Huang et al. 2013).

##### Influence of pH

The extraction experiments involving different pH values of aqueous buffers (5 mL of ACN or 5 mL of MeOH + 5 mL of McIlvaine buffer of specific pH; Fig. S6) yielded varied results across PhAC groups, highlighting the challenge of identifying a universally optimal pH due to the broad range of their pKa values. McIlvaine buffer is frequently employed in various studies by Bian et al. (2015); Ferhi et al. (2016); Hang et al. (2021); Silva et al. (2020), known for its ability to be prepared across a broad range of pH values. Additionally, its citric acid content, with chelating properties, contributes to improved extraction efficiency of pharmaceuticals from the soil matrix (Deschamps et al. 2014). For instance, the overall RR at pH 2.6 was 10% with ACN and 24% with MeOH, at pH 4.0 was 37% with ACN and 31% with MeOH, and at pH 8.0 was 43% with ACN and 29% with MeOH. Discrepancies in scientific studies further complicate the determination of the most suitable pH for PhAC extraction. While some studies by Carballo et al. (2007); Golovko et al. (2016) advocate for low-pH extraction mediums to achieve high RR (ensuring certain PhACs are in their cationic state), this contradicts our findings where the overall optimal RR was achieved at pH 8.0. In our study, higher RR was consistently observed when PhACs were in their anionic or zwitterionic form, which was attributed to the negatively charged pH of the buffer and soil/organic matter (humic and fulvic acids). This aligns with studies employing buffers with alkaline pH (Ferhi et al. 2016). Given the absence of an ideal buffer pH applicable to all compounds, our study introduced a novel approach involving multi-step extraction with varying pH values. This approach resulted in an overall RR of 78% in the final method for the extraction of pharmaceuticals from soil, offering a pragmatic compromise among the complexity of PhAC behavior under different pH conditions.

##### Influence of extraction mechanism

The composition of the EM (overview of EM in Table S8), plays a crucial role in influencing the extraction mechanism. This is evident in cases such as the MgNO_3_ extraction mechanism, where ionic interactions or metal complexation are applied (Turiel et al. 2006). A notable difference was observed between EM14 (utilizing metal complexation mechanism with 0.6 g EDTA + 7.5 mL ACN + 7.5 mL McIlvaine buffer pH 6.0 + 0.2 mL 2.5% NH_3_ + 4.8 mL 0.5 g·mL^−1^ Mg(NO_3_)_2_·6H_2_O) and EM2 (5 mL MeOH + 5 mL Milli-Q water), as can be seen in Fig. S7. EM14, employing metal complexation, achieved an overall RR of 48%, with specific recovery rates for FQs at 35%, TCs at 45%, and SAs at 48%, among others. In contrast, EM2 resulted in an overall RR of 28%, with specific recovery rates for FQs at 6%, TCs at 2%, and SAs at 75%. Therefore, a combination of two EMs with different extraction mechanisms would be suitable for the comprehensive extraction of multiple residues. Therefore, the final extraction method in our study incorporated different extraction mechanisms, with variations in the composition of the EM during the four-step extraction process for PhACs in soil.

##### Influence of EDTA

Articles from Ferhi et al. (2016); Hu et al. (2012) and Mirzaei et al. (2017) have highlighted that TCs, FQs, and MLs may form chelates with di- and trivalent metal cations (e.g., Ca^2+^, Mg^2+^, Al^3+^) in the clay mineral interlayers or hydroxyl groups at the soil particle surface. Therefore, chelating agents such as EDTA (or citric acid, oxalic acid, or their buffers) are commonly employed to enhance the recovery rates of PhACs from soil samples. The addition of EDTA can also elevate the ionic strength, potentially improving the solubility of PhACs in the organic solvent of the EM and enhancing their RR (Ferhi et al. 2016). However, an excessive amount of EDTA may chelate not only metals but also organic compounds (Hu et al. 2012). Studies from Huang et al. (2013) and Zhang et al. (2021) have utilized varying amounts of EDTA, ranging from 0.01 to 0.6 g, recognizing that the optimal quantity can depend on the pharmaceutical class and soil properties. In this study, two different extraction media, EM14 and EM2, featuring different EDTA amounts (0, 0.6, and 1.2 g), were tested (Fig. S8). EM14 achieved the highest overall RR of 46% with 0.6 g EDTA, predominantly in pharmaceutical groups such as beta blockers, TCs, MLs, and FQs. EM2 reached the highest overall RR of 33% with 1.2 g EDTA across all pharmaceutical groups except SAs. Interestingly, for both EMs, the RR of SAs was highest without EDTA. The lack of complementarity in EDTA amounts between EM14 and EM2 may stem from their different compositions and extraction mechanisms. EM14 already contains a high amount of salts (MgNO_3_) and citric acid in the form of McIlvaine buffer, potentially explaining why, in EM2, increasing EDTA leads to higher RR. Additionally, different pH values could influence extraction equilibrium and complex stability. While an article from Deschamps et al. (2014) suggests that the RR of FQs and TCs increases in the order of no ligand < citrate < EDTA, another study from Ferhi et al. (2016) found that the presence of EDTA decreases the RR of all PhACs except fluoroquinolones and tetracyclines. The absence of a universal EDTA amount for the extraction of several PhACs necessitates a compromise in selecting a single EDTA amount with the best overall results. An alternative approach is to use several steps of ultrasound-assisted extraction (UAE) with varying amounts of EDTA between cycles, similar to this study and a study from Ferhi et al. (2016), where a two-step UAE was performed, the first without EDTA and the second with EDTA.

#### Influence of extraction temperature in the ultrasound bath

Temperature is a crucial factor in modulating extraction equilibrium, given the varying thermodynamics (ΔH) of endothermic (ΔH > 0) and exothermic (ΔH < 0) processes. As indicated in studies from Albero et al. (2015) and Ferrero et al. (2015), temperature is one of the most important extraction conditions to optimize. In particular, the temperature of the ultrasonic bath plays a significant role in influencing RR by affecting the solubility of PhACs in the EM and inducing cavitation phenomena that affect mass transfer (Albero et al. 2015). Elevated bath temperatures facilitate the permeation of EM into the sample matrix by reducing solvent viscosity, although it may lead to enhanced co-elution of interference compounds and potential degradation of the target analyte (Albero et al. 2015). Contrary to other studies and our findings, a study by Turiel et al. (2006) contends that temperature does not influence RR, although it focused solely on the extraction of quinolones and fluoroquinolones. This discrepancy underscores the complexity of the extraction process, where multiple factors interact, and the impact of temperature may vary depending on the specific conditions and compounds involved.

To understand the influence of temperature on extraction efficiency, experiments were conducted at various temperatures, including 12 °C, 35 °C, 45 °C, and 55 °C (Fig. S9). To comprehensively assess whether temperature serves as a universal parameter to enhance RR, these experiments were conducted using two distinct extraction mediums (EMs), each employing different extraction mechanisms. Notably, the highest overall RR was observed at temperatures of 35 °C (EM14, average RR 42%) and 45 °C (EM2, average RR 26%). Specifically, optimal temperatures for the extraction of beta blockers were 35 °C (EM14) and 25 °C (EM2), for TCs at 12 °C in both EM cases, for SAs at 25 °C in both EMs, and for FQs at 35 °C in both EMs. MLs showed the highest RR at 35 °C (EM14) and 12 °C (EM2). NSAIDs and topical products exhibited the highest RR at 25 °C (EM14) and 45 °C (EM2), while antiepileptics demonstrated peak RR at 25 °C (EM14) and 12 °C (EM2). Although the results obtained using either EM are quite similar, indicating that temperature is a potentially universal parameter in UAE, there is no one-size-fits-all temperature for the extraction of a broad spectrum of PhACs. Therefore, a compromise must be made in selecting a single temperature that yields the best overall results. Alternatively, a multi-step UAE approach with varying temperatures between cycles, similar to previous studies from Aznar et al. (2013) and Ferhi et al. (2016), could be considered.

### Optimization of extraction procedures for lettuce leave samples

Initially, a comprehensive literature review on the extraction of pharmaceuticals from vegetables was conducted. Several studies have previously developed QuEChERS methods for pharmaceutical extraction (Caldú and Diaz-Cruz 2021; Chen et al. 2020; Chuang et al. 2015; Yu et al. 2017). While experiments were conducted on the basis of these studies, the most successful results were achieved by employing the QuEChERS method outlined in study from Chuang et al. (2015), as can be seen in Fig. S10. In that study, the extraction of 10 pharmaceuticals from lettuce using the QuEChERS method resulted in an overall average RR of 82 ± 6%, with the extraction medium composed of MeOH:H_2_O:ACN in a ratio of 8:20:72. Subsequently, we expanded upon this method, successfully extracting 42 pharmaceuticals from lettuce with an overall average RR of 78 ± 6%. This prompted further optimization of the QuEChERS method, involving the investigation of parameters such as the composition of the extraction medium (including pH of aqueous buffer), the quantity of EDTA, and the composition of dSPE.

#### Influence of pH

Optimizing the extraction process for pharmaceuticals, considering their diverse pKa values, required further adjustments to the pH of the aqueous phase. Milli-Q water and McIlvaine buffer were used across a range of pH values (2.6 to 8.0), Fig. S11. Given that pH influences the charge of pharmaceuticals and, consequently, their extraction yields, recovery rates were assessed as follows: 78 ± 6% with H_2_O, 90 ± 7% with buffer pH 2.6, 88 ± 7% with buffer pH 4.0, 75 ± 5% with buffer pH 6.0, 94 ± 7% with buffer pH 7.0, and 80 ± 5% with buffer pH 8.0. pH 2.6 was deemed the most suitable because it yielded the highest extraction efficiency (70–130%) for most pharmaceuticals. It is noteworthy that favorable recovery rates were achieved across the entire pH spectrum during the extraction of pharmaceuticals from lettuce. This observation contrasts with extraction from soil, where adjusting the pH of the aqueous buffer plays a crucial role in the extraction process. The study by He et al. (2018) investigated the impact of pH using a McIlvaine buffer within the pH range of 2–4. Notably, no significant differences between buffers were observed for SAs, whereas the RR of MLs and TCs increased at pH 2.4, aligning with our findings. This phenomenon could potentially be attributed to the heightened stability of drugs at acidic pH or an increased extraction efficiency due to the charge of the pharmaceuticals, subsequently altering the oil/water partition coefficient (He et al. 2018; Yu et al. 2017). Other studies focusing on the extraction of PhACs from vegetables have also utilized acidified extraction media, such as acetic acid (Chen et al. 2020; Chung et al. 2017).

#### Influence of EDTA

Similar to the extraction of pharmaceuticals from soil, the addition of EDTA has been theorized to potentially enhance the RR of PhACs while concurrently reducing the complexation of TCs and FQs with metal cations (Chuang et al. 2015). Although studies have applied the addition of EDTA (Chuang et al. 2015), no studies have specifically explored the impact of EDTA addition on the RR of PhACs from lettuce or other leafy vegetables. Within this study, the optimization of EDTA quantity in the pharmaceutical extraction process revealed negligible effects on yields: 89 ± 3% without EDTA; 88 ± 2% with 10 mg of EDTA, and 93 ± 7% with the addition of 20 mg of EDTA (Fig. S12). Although presence of citric acid in McIlvaine buffer can be sufficient for chelation of present cations, unlike in the context of soil extraction. This suggests that in scenarios where the buffer itself contains chelating agents, the additional use of EDTA may not significantly alter the recovery rates.

#### Influence of dSPE sorbents

PSA, C18 and GCB have been used in previous studies by Chen et al. (2020); He et al. (2018); Lawal et al. (2018); Yu et al. (2017) as purification sorbents. The initial composition of dSPE sorbents, as per the study from Chuang et al. (2015), included 0 mg GCB, 12.5 mg DSC-18, 12.5 mg PSA, and 225 mg of anhydrous Na_2_SO_4_. Given that GCB is highly effective in removing pigments from plant matrices, potentially reducing the matrix effect, we investigated the impact of varying amounts of GCB on RR. The following RRs were achieved: 88 ± 5% with 0 mg of GCB; 72 ± 5% with 10 mg of GCB; 71 ± 3% with 20 mg of GCB, and 67 ± 5% with 30 mg of GCB (Fig. S13). Our experiments revealed that the inclusion of GCB in dSPE not only removes pigments but also affects the recovery of certain analytes, such as b-blockers (pindolol), throat preparations (flurbiprofen), FQs (moxifloxacin, norfloxacin, ofloxacin), SAs (sulfadimethoxine, sulfamerazine, sulfamethazine, sulfamethoxazole and sulfamethoxypyridazine), to varying extents. This adverse effect of GCB has been previously reported in studies by He et al. (2018); Lawal et al. (2018); and Yu et al. (2017). Consequently, GCB was excluded from the final method, leading to increased matrix effects. However, these effects can be effectively compensated by implementing matrix calibration. Moreover, study by He et al. (2018) have not only examined the impact of GCB but also investigated the effects of PSA and C18. The findings revealed that C18 exhibited negligible adsorption of PhACs, whereas a reduction in RR was observed for FQs, MLs, TCs, and β-lactams in clean-up processes employing > 25 mg of PSA.

### Optimization of extraction procedures for earthworm samples

The QuEChERS method employed for the extraction of PhACs from earthworms was adapted from an earlier extraction method designed for PhACs from lettuce. During method development, freeze-dried earthworms were intentionally spiked with known concentrations of PhACs. The optimization process focused on key parameters, including the composition of the extraction medium (varied organic solvents and pH of aqueous buffer), the quantity of EDTA, and the composition of the dSPE.

#### Influence of organic solvents

Extraction experiments with different ratios of organic solvents were performed during method optimisation. The obtained recovery rates were as follows: 75 ± 2% with MeOH:H_2_O:ACN (8:20:72), 78 ± 2% with MeOH:H_2_O (80:20), and 73 ± 5% with ACN:H_2_O (80:20), (Fig. S14). Opting for MeOH:H_2_O (80:20) with a recovery rate of 78 ± 2%, which demonstrated the extraction of the highest number of pharmaceuticals within the efficiency range of 70–130%, was considered the most suitable. However, no significant influence on recovery rates was observed, possibly because of the relatively straightforward nature of the matrix compared with the complexity of the soil matrix. In contrast to our results, previous studies by Bergé and Vulliet (2015) and Montemurro et al. (2021) have utilized acetonitrile for the extraction of PhACs, although these studies did not investigate impact of the organic solvent on RRs.

#### Influence of pH

During the optimization of the extraction method for the earthworm matrix, we investigated the impact of the pH of the aqueous phase on recovery rates (using both Milli-Q water and McIlvaine buffer within the pH range of 2.6 to 8.0). The recovery rates obtained were as follows: 81 ± 4% with H_2_O; 103 ± 3% with buffer pH 2.6; 81 ± 10% with buffer pH 4.0; 77 ± 4% with buffer pH 6.0; 79 ± 4% with buffer pH 7.0, and 76 ± 3% with buffer pH 8.0 (Fig. S15). Opting for pH 2.6 was considered the most suitable because it resulted in the extraction of the highest number of pharmaceuticals within the efficiency range of 70–130%. However, consistently high RRs were achieved across the entire pH spectrum when extracting pharmaceuticals from earthworms, similar to the findings in lettuce extraction. Meanwhile, study by Bergé and Vulliet (2015) employed a combination of organic solvent and Milli-Q water for the efficient extraction of veterinary antibiotics from earthworm tissue with RR 45.2–105%.

#### Influence of EDTA

Extraction experiments were conducted by adding 0, 10, 20, and 30 mg of EDTA. The resulting RRs were as follows: 121 ± 11% with 0 mg of EDTA; 81 ± 6% with 10 mg of EDTA; 76 ± 4% with 20 mg of EDTA, and 77 ± 2% with 30 mg of EDTA (Fig. S16). Opting for the addition of 10 mg of EDTA was considered the most suitable, as it led to the extraction of the highest number of pharmaceuticals within the efficiency range of 70%–130% while maintaining a low RSD%. Higher average RRs were achieved without the addition of EDTA, although this was caused by certain analytes exceeding the RR of 100%. The objective of method optimization also includes reducing the RSD during sample preparation. The study by Montemurro et al. (2021) described that the introduction of EDTA resulted in an increase in RRs for earthworm samples, which was attributed to the metal complexation of cations present in the soil environment.

#### Influence of dSPE sorbents

In line with previous studies by Bergé and Vulliet (2015) and Montemurro et al. (2021), the utilization of a combined PSA/C18 sorbent system was explored.The initial composition of the dSPE sorbents included 12.5 mg DSC-18, 12.5 mg PSA, and 225 mg of anhydrous Na_2_SO_4_. The coexistence of both dSPE sorbents likely establishes a favorable hydrophilic-lipophilic balance, facilitating the transit of target compounds through the organic phase (Bergé and Vulliet 2015). The function of DSC-18 is to eliminate lipids, sterols, and other non-polar substances in earthworm extracts. To optimize the method, various amounts of DSC-18 were tested to assess their impact on RRs. The results were as follows: 97 ± 4% with 0 mg of DSC-18; 99 ± 2% with 12.5 mg of DSC-18; 83 ± 2% with 20 mg of DSC-18; 81 ± 2% with 30 mg of DSC-18, and 87 ± 3% with 40 mg of DSC-18 (Fig. S17). The most suitable amount, 12.5 mg of DSC-18, was selected because it allowed the extraction of the highest number of pharmaceuticals within an efficiency range of 70–130%. Similar to other studies by Bergé and Vulliet (2015) and Montemurro et al. (2021), this decision aimed to strike a balance between achieving high RR with low RSD% and matrix removal. Moreover, a study by Montemurro et al. (2021) revealed that the use of PSA can result in a notable decrease in the recovery RRs of PhACs.

### Method validation

Validation is acknowledged as a crucial step because it demonstrates the reliability and consistency of the analytical method, thereby enabling its application to real samples. The criteria employed for validation encompassed the determination of MLoD, MLoQ, linearity, recovery rate, intra- and inter-day precisions, selectivity/specificity, and identity confirmation.

#### Soil

To quantify PhACs in soil, an external calibration method incorporating isotopically labeled internal standards (IS) was employed (Table S6). The calibration range for all PhACs extended from 0.1 to 500 ng·mL^−1^ (equivalent to 0.1–500 ng·g^−1^ dry weight (dw) of soil), aligning with reported concentrations in terrestrial environments. Coefficients of determination (R^2^) exceeding 0.995 for all PhACs confirmed linearity. The validation results for individual PhACs, including MLoD, MLoQ, RRs, inter-day RSD, and ME, are presented in Table 1.

**Table 1:** Validation results of the multiresidual extraction method for pharmaceuticals from soil (MLoD – Method Limit of Detection; MLoQ – Method Limit of Quantification; RR – Recovery Rate; Inter-day RSD – Inter-day Relative Standard Deviation; and ME – Matrix Effect)

Pharmaceutical group	Analyte name	MLoD [ng·g^−1^]	MLoQ [ng·g^−1^]	RR 25 ng·g^−1^ [%]	RR 50 ng·g^−1^ [%]	RR 100 ng·g^−1^ [%]	RR 250 ng·g^−1^ [%]	RR 500 ng·g^−1^ [%]	Inter-day RSD [%]	ME [%]
Antiepileptics	Carbamazepine	0.8	2.5	100 ± 4	97 ± 5	93 ± 3	79 ± 4	72 ± 2	4	94
Beta Blockers	Acebutolol	0.7	2.1	95 ± 4	94 ± 5	91 ± 5	81 ± 3	68 ± 5	5	90
	Atenolol	N.D	N.D	< 10	11 ± 2	16 ± 3	19 ± 7	19 ± 15	38	107
	Nadolol	0.05	0.15	133 ± 1	116 ± 4	105 ± 4	76 ± 5	73 ± 6	5	106
	Pindolol	N.D	N.D	< 10	< 10	< 10	< 10	< 10	N.D	50
	Propranolol	1.3	4.0	50 ± 3	50 ± 2	50 ± 2	55 ± 8	51 ± 12	10	83
Fluoroquinolone antibacterials	Ciprofloxacin	0.5	1.5	59 ± 2	73 ± 2	74 ± 8	76 ± 4	75 ± 9	7	88
	Enrofloxacin	0.1	0.3	65 ± 10	75 ± 3	50 ± 6	75 ± 5	78 ± 7	9	96
	Moxifloxacin	9.9	30	64 ± 4	52 ± 7	63 ± 4	88 ± 7	94 ± 5	8	120
	Norfloxacin	1.4	4.2	59 ± 4	56 ± 12	69 ± 10	94 ± 5	84 ± 12	12	95
	Ofloxacin	1.0	3.1	58 ± 5	63 ± 13	66 ± 12	80 ± 7	86 ± 11	14	103
	Pefloxacin	0.5	1.6	61 ± 6	59 ± 13	64 ± 12	80 ± 3	77 ± 8	13	93
Macrolides	Azithromycin	5.2	15.7	56 ± 4	60 ± 5	75 ± 2	86 ± 6	84 ± 2	6	100
	Clarithromycin	2.0	6.1	82 ± 5	77 ± 3	71 ± 2	105 ± 9	101 ± 7	6	136
	Erythromycin	0.3	1.0	63 ± 10	68 ± 4	67 ± 8	84 ± 4	79 ± 6	9	90
	Roxithromycin	1.1	3.2	91 ± 7	82 ± 4	70 ± 3	115 ± 5	121 ± 12	6	141
Nonsteroidal anti-inflammatory drugs	Celecoxib	N.D	N.D	< 10	< 10	< 10	< 10	< 10	N.D	104
	Ketoprofen	0.9	2.7	55 ± 17	51 ± 4	50 ± 8	54 ± 7	47 ± 17	21	109
	Mefenamic acid	N.D	N.D	< 10	< 10	< 10	< 10	< 10	N.D	45
	Meloxicam	N.D	N.D	< 10	< 10	< 10	< 10	< 10	N.D	106
	Naproxen	2.4	7.2	29 ± 12	24 ± 1	24 ± 7	24 ± 18	27 ± 4	33	88
	Piroxicam	0.6	1.7	76 ± 2	73 ± 3	77 ± 2	54 ± 4	48 ± 9	7	110
Sulfonamides and Trimethoprim	Sulfacetamide	0.2	0.7	31 ± 2	34 ± 3	36 ± 2	38 ± 14	45 ± 9	16	88
	Sulfadiazine	0.04	0.14	74 ± 5	53 ± 3	53 ± 2	63 ± 14	70 ± 9	10	69
	Sulfadimethoxine	0.14	0.40	50 ± 2	54 ± 4	49 ± 6	60 ± 18	67 ± 5	12	71
	Sulfamerazine	0.35	1.04	48 ± 2	42 ± 4	50 ± 2	74 ± 14	83 ± 10	10	71
	Sulfamethazine	1.2	3.5	60 ± 3	64 ± 4	68 ± 2	94 ± 6	91 ± 7	6	80
	Sulfamethoxazole	1.3	3.8	50 ± 3	40 ± 4	46 ± 2	71 ± 15	76 ± 5	10	76
	Sulfamethoxypyridazine	0.09	0.29	34 ± 3	35 ± 2	41 ± 2	62 ± 15	68 ± 5	10	75
	Sulfapyridine	1.5	4.5	40 ± 2	42 ± 3	37 ± 2	67 ± 18	79 ± 10	11	86
	Sulfasalazine	N.D	N.D	< 10	< 10	< 10	< 10	< 10	N.D	122
	Sulfathiazole	2.1	6.3	40 ± 2	39 ± 3	45 ± 1	61 ± 18	69 ± 9	11	72
	Trimethoprim	1.9	5.6	80 ± 4	76 ± 3	75 ± 1	91 ± 3	72 ± 13	6	109
Tetracyclines	Chlortetracycline	3.0	9.1	109 ± 6	100 ± 8	96 ± 4	125 ± 21	97 ± 10	9	262
	Doxycycline	0.9	2.6	84 ± 4	72 ± 6	75 ± 4	108 ± 2	81 ± 5	5	149
	Minocycline	N.D	N.D	< 10	< 10	< 10	< 10	< 10	N.D	157
	Oxytetracycline	0.15	0.45	111 ± 5	105 ± 10	105 ± 4	118 ± 4	98 ± 11	8	185
	Tetracycline	3.7	11	83 ± 13	69 ± 6	63 ± 7	86 ± 1	71 ± 5	9	135
Throat Preparations	Flurbiprofen	N.D	N.D	< 10	< 10	< 10	< 10	< 10	N.D	92
Topical products for joint and muscular pain	Diclofenac	N.D	N.D	< 10	< 10	< 10	< 10	< 10	N.D	118
	Indomethacin	N.D	N.D	< 10	< 10	< 10	< 10	< 10	N.D	120
	Nimesulide	1.9	5.6	63 ± 9	59 ± 5	64 ± 4	79 ± 4	63 ± 5	8	105

The calculated MLoDs fall within the low ng·g^−1^ range for most compounds, except 10 PhACs where MLoD and MLoQ were not determined, as their RR% were < 10% (Beta blockers—atenolol, pindolol; SAs – sulfasalazine; NSAIDs—celecoxib, mefenamic acid, meloxicam; TCs – minocycline; Throat Preparations – flurbiprofen; Topical products for joint and muscular pain – diclofenac, indomethacin). Similar MLoD values are reported in the available literature, presenting extraction methods for PhACs from soil (Table S2); in some cases, lower MLoDs in the range of tenths to hundredths of ng·g^−1^ are reported. MLoD and MLoQ values are influenced by the calculation method (differing by up to an order of magnitude) (Kruve et al. 2015a), the ratio of sample weight to extraction solvent, concentration steps, the sensitivity of the instrument, recovery rates and also matrix effects (Kruve et al. 2015b). Moreover, in alignment with Bergé and Vulliet (2015), MLoDs significantly differ even within the same pharmaceutical group.

Recovery rates were determined by spiking blank soil at five concentration levels (25, 50, 100, 250, and 500 ng·g^−1^). Among the 42 pharmaceuticals analyzed in soil, nine exhibited insufficient recovery rates (< 10%), whereas 29 out of 42 compounds showed average recovery rates exceeding 50%, with intra- and inter-day RSD below 25%. These 29 compounds had an average RR of 73%, ranging from 50.8% to 107.4%, which is consistent with the studies presented in Table S2, although the majority of these studies have focused on a smaller number of pharmaceutical groups, thus covering a narrower range of compounds.

Sample preparation resulted in a matrix effect exceeding 30% in 10 of the 40 PhACs. The study by Golovko et al. (2016) reported similar matrix effects, while also noting variations in matrix effects among different soils.

#### Lettuce

The extraction method for extracting PhACs was validated for both lettuce leaves and roots, yielding similar results. However, only the results for lettuce leaves are thoroughly discussed below, as quantifying PhACs in edible parts of lettuce is of greater importance due to potential uptake and future regulatory policies.

To quantify PhACs in lettuce leaves, we employed a matrix-matched external calibration to mitigate matrix effects. The calibration matrix was prepared by spiking lettuce blank extracts with known concentrations of PhACs, which were extracted using the optimized method. The calibration spanned 0.5 to 100 ng·mL^−1^ (equivalent to 20–4,000 ng·g^−1^ dw of lettuce), aligning with concentrations reported in lettuce and other leafy vegetables (ranging from ng·g^−1^ to μg·g^−1^) (Kodešová et al. 2019). Coefficients of determination (R^2^) exceeding 0.995 for all PhACs confirmed linearity. The validation results for individual PhACs, including MLoD, MLoQ, RRs, inter-day RSD, and ME, are presented in Table 2.

**Table 2:** Validation results of the multiresidual extraction method for pharmaceuticals from lettuce leaves (MLoD – Method Limit of Detection; MLoQ – Method Limit of Quantification; RR – Recovery Rate; Inter-day RSD – Inter-day Relative Standard Deviation; and ME – Matrix Effect)

Pharmaceutical group	Analyte name	MLoD [ng·g^−1^]	MLoQ [ng·g^−1^]	RR 250 ng·g^−1^ [%]	RR 500 ng·g^−1^ [%]	RR 1,000 ng·g^−1^ [%]	RR 2,000 ng·g^−1^ [%]	RR 2,500 ng·g^−1^ [%]	Inter-day RSD [%]	ME [%]
Antiepileptics	Carbamazepine	8.8	26	130 ± 24	120 ± 27	120 ± 15	101 ± 9	88 ± 6	15	136
Beta Blockers	Acebutolol	15	45	113 ± 23	131 ± 27	126 ± 30	91 ± 24	88 ± 10	30	48
	Atenolol	60	181	< 10	129 ± 5	113 ± 20	85 ± 23	66 ± 11	29	41
	Nadolol	44	132	69 ± 8	69 ± 8	114 ± 9	85 ± 8	81 ± 10	15	23
	Pindolol	28	83	114 ± 11	99 ± 18	128 ± 26	98 ± 28	84 ± 9	22	15
	Propranolol	31	94	135 ± 13	119 ± 17	120 ± 16	122 ± 14	77 ± 3	25	58
Fluoroquinolone Antibacterials	Ciprofloxacin	52	156	79 ± 27	132 ± 13	96 ± 15	67 ± 6	77 ± 9	21	96
	Enrofloxacin	33	100	117 ± 23	121 ± 12	110 ± 27	77 ± 24	77 ± 1	25	180
	Moxifloxacin	144	432	< 10	< 10	117 ± 17	106 ± 21	73 ± 3	27	171
	Norfloxacin	77	231	< 10	109 ± 42	79 ± 18	65 ± 9	71 ± 8	28	107
	Ofloxacin	56	167	116 ± 18	114 ± 24	121 ± 13	107 ± 10	78 ± 12	27	44
	Pefloxacin	54	163	90 ± 19	136 ± 17	124 ± 20	85 ± 9	76 ± 8	28	199
Macrolides	Azithromycin	23	70	< 10	103 ± 16	90 ± 25	92 ± 11	85 ± 11	16	23
	Clarithromycin	11	32	114 ± 24	124 ± 27	122 ± 25	117 ± 24	82 ± 4	24	66
	Erythromycin	147	440	< 10	< 10	48 ± 16	85 ± 24	75 ± 24	21	21
	Roxithromycin	21	64	119 ± 16	197 ± 18	118 ± 11	135 ± 10	87 ± 13	22	83
Nonsteroidal anti-inflammatory drugs	Celecoxib	6	18	118 ± 8	129 ± 11	120 ± 7	98 ± 7	88 ± 12	23	95
	Ketoprofen	7	22	149 ± 13	137 ± 11	154 ± 11	115 ± 12	87 ± 9	30	43
	Mefenamic acid	34	101	106 ± 22	91 ± 23	76 ± 19	78 ± 8	111 ± 29	29	89
	Meloxicam	7	22	113 ± 13	144 ± 26	128 ± 14	74 ± 17	72 ± 5	24	40
	Naproxen	41	122	131 ± 16	112 ± 16	115 ± 11	107 ± 18	95 ± 11	25	56
	Piroxicam	23	69	119 ± 20	114 ± 12	145 ± 13	104 ± 8	82 ± 7	29	90
Sulfonamides and Trimethoprim	Sulfacetamide	65	196	58 ± 9	60 ± 10	72 ± 3	69 ± 15	73 ± 7	17	100
	Sulfadiazine	118	354	51 ± 8	73 ± 3	85 ± 28	84 ± 17	72 ± 2	26	52
	Sulfadimethoxine	46	137	108 ± 17	83 ± 6	125 ± 12	97 ± 21	73 ± 6	23	43
	Sulfamerazine	32	95	84 ± 19	64 ± 13	125 ± 15	88 ± 20	71 ± 5	26	229
	Sulfamethazine	50	151	67 ± 24	102 ± 29	90 ± 18	80 ± 15	79 ± 3	23	209
	Sulfamethoxazole	29	87	110 ± 22	89 ± 19	114 ± 29	79 ± 17	71 ± 6	27	72
	Sulfamethoxypyridazine	56	169	61 ± 22	85 ± 17	87 ± 28	57 ± 20	51 ± 6	30	106
	Sulfapyridine	78	235	61 ± 7	54 ± 7	90 ± 24	91 ± 22	63 ± 4	21	128
	Sulfasalazine	57	170	75 ± 18	133 ± 17	121 ± 9	104 ± 6	82 ± 13	12	112
	Sulfathiazole	48	144	54 ± 11	55 ± 9	98 ± 25	100 ± 20	60 ± 3	30	88
	Trimethoprim	26	77	115 ± 23	70 ± 25	109 ± 15	73 ± 11	88 ± 13	24	50
Tetracyclines	Chlortetracycline	28	83	81 ± 7	97 ± 6	67 ± 17	59 ± 10	61 ± 6	30	359
	Doxycycline	66	198	86 ± 22	94 ± 21	96 ± 21	70 ± 11	113 ± 23	29	180
	Minocycline	184	551	< 10	< 10	38 ± 6	28 ± 3	39 ± 10	26	181
	Oxytetracycline	71	215	29 ± 8	40 ± 22	59 ± 17	43 ± 12	50 ± 5	20	283
	Tetracycline	26	77	127 ± 14	105 ± 28	98 ± 13	74 ± 7	69 ± 14	21	111
Throat Preparations	Flurbiprofen	40	119	126 ± 23	110 ± 21	104 ± 15	91 ± 13	76 ± 12	16	63
Topical products for joint and muscular pain	Diclofenac	16	47	77 ± 16	98 ± 21	88 ± 16	78 ± 17	88 ± 16	16	90
	Indomethacin	14	43	110 ± 29	109 ± 23	98 ± 12	79 ± 4	102 ± 26	14	68
	Nimesulide	8	25	117 ± 13	110 ± 9	118 ± 6	119 ± 6	96 ± 4	25	64

The calculated MLoDs are within the tens to hundreds of ng·g^−1^, albeit relatively higher than those reported in the existing literature (Table S3). The calculated MLoDs within our study are comparable to or higher than those reported in studies from Chuang et al. (2015) and Sallach et al. (2015), while being in the range of tens to units of ng·g^−1^. It is crucial to acknowledge the distinction in our study, where a lower sample weight of lyophilized lettuce (0.1 g) was utilized, in contrast to other studies employing 0.5–2.0 g of sample weight (Chuang et al. 2015; Chung et al. 2017; Montemurro et al. 2017), and even up to 10 g in some cases (Chung et al. 2017; He et al. 2018; Yu et al. 2017). Our research employed a reduced sample weight, a factor contributing to elevated MLoDs. This approach facilitates the examination of young lettuce samples, particularly when harvested at the onset of the exposure period (e.g. 14 days). This consideration is crucial, given that fresh lettuce generally comprises approximately 95% water (Tao et al. 2022). In addition, our instrumental LoDs are affected by the use of a low-end mass spectrometer. Method LoDs could be enhanced by adopting more sensitive instruments, as demonstrated in other studies. It is noteworthy that MLoDs are influenced by various factors, including the calculation method, which can vary by up to an order of magnitude (Kruve et al. 2015a), the ratio of sample weight to extraction solvent, preconcentration steps, recovery rates and matrix effects (Kruve et al. 2015b).

The RR of PhACs was assessed by spiking blank lettuce leaf extracts at five concentration levels (250 ng·g^−1^, 500 ng·g^−1^, 1,000 ng·g^−1^, 2,000 ng·g^−1^, and 2,500 ng·g^−1^ of lettuce dry weight). Lower RR values (below 10%) were observed only at the lowest spike concentration (250 ng·g^−1^) for 6 out of the 42 pharmaceuticals because of the low sensitivity of the MS instrument. However, an average RR greater than 50% was achieved for 39 out of the 42 pharmaceuticals (except erythromycin, minocycline and mefenamic acid), with an RSD% below 30%. These 40 PhACs demonstrated an average RR across the entire concentration range of 94%, ranging from 59 to 131%. This performance is notably favorable when compared with other studies addressing the extraction of PhACs from lettuce (Chuang et al. 2015; Montemurro et al. 2017; Sallach et al. 2015), where the spectrum of validated PhACs was lower (11, 4, and 13, respectively) with similar recovery rates.

It is important to note that, in our method, the absence of graphitized carbon black (GCB) to remove pigments, owing to the adsorption of target compounds, led to significant matrix effects exceeding 30% for 28 of the 42 pharmaceuticals. This observation aligns with a study by He et al. (2018) in which substantial matrix effects were also reported. However, it is crucial to mention that these matrix effects were mitigated to some extent by the use of a matrix-matched external calibration.

#### Earthworms

To quantify PhACs in earthworms, we employed a matrix-matched external calibration to mitigate matrix effects. The calibration matrix was prepared by spiking earthworm blank extracts with known concentrations of PhACs, which were extracted using the optimized method. The calibration spanned 0.5 to 100 ng·mL^−1^ (equivalent to 20–4,000 ng·g^−1^ dw), aligning with the concentrations reported in earthworms (ranging from ng·g^−1^ to μg·g^−1^) (Carter et al. 2014; Kinney et al. 2008). Coefficients of determination (R^2^) exceeding 0.995 for all PhACs confirmed linearity. The validation results for individual PhACs, including MLoD, MLoQ, RRs, inter-day RSD, and ME, are presented in Table 3.

**Table 3:** Validation results of the multiresidual extraction method for pharmaceuticals from earthworms (MLoD – Method Limit of Detection; MLoQ – Method Limit of Quantification; RR – Recovery Rate; Inter-day RSD – Inter-day Relative Standard Deviation; and ME – Matrix Effect)

Pharmaceutical group	Analyte name	LoD [ng·g^−1^]	LoQ [ng·g^−1^]	RR 250 ng·g^−1^ [%]	RR 500 ng·g^−1^ [%]	RR 1,000 ng·g^−1^ [%]	RR 2,000 ng·g^−1^ [%]	RR 2,500 ng·g^−1^ [%]	Inter-day RSD [%]	ME [%]
Antiepileptics	Carbamazepine	3.7	11	97 ± 3	91 ± 2	87 ± 4	81 ± 2	101 ± 2	13	88
Beta Blockers	Acebutolol	4.3	13	110 ± 1	96 ± 2	89 ± 3	83 ± 3	88 ± 5	9	64
	Atenolol	4.8	14	107 ± 4	94 ± 5	87 ± 3	81 ± 6	80 ± 10	17	35
	Nadolol	3.1	9.2	121 ± 3	102 ± 1	89 ± 2	88 ± 5	100 ± 20	10	29
	Pindolol	6.5	19	108 ± 2	89 ± 1	77 ± 1	76 ± 4	89 ± 6	14	38
	Propranolol	11	32	106 ± 4	81 ± 3	63 ± 2	55 ± 2	87 ± 6	18	19
Fluoroquinolone Antibacterials	Ciprofloxacin	45	136	112 ± 10	92 ± 1	84 ± 5	79 ± 3	70 ± 7	9	141
	Enrofloxacin	51	154	59 ± 13	51 ± 16	42 ± 17	39 ± 12	80 ± 8	19	367
	Moxifloxacin	30	89	109 ± 10	89 ± 5	74 ± 6	68 ± 7	71 ± 1	17	201
	Norfloxacin	17	50	122 ± 4	95 ± 5	86 ± 1	82 ± 2	73 ± 4	13	309
	Ofloxacin	16	47	122 ± 2	94 ± 2	85 ± 2	81 ± 2	110 ± 16	7	193
	Pefloxacin	20	59	118 ± 8	90 ± 1	84 ± 4	79 ± 1	78 ± 2	7	303
Macrolides	Azithromycin	46	139	109 ± 7	88 ± 10	37 ± 2	25 ± 9	83 ± 2	6	37
	Clarithromycin	24	71	103 ± 6	92 ± 1	85 ± 5	81 ± 3	84 ± 1	15	50
	Erythromycin	113	338	61 ± 13	49 ± 24	46 ± 21	45 ± 19	43 ± 9	25	328
	Roxithromycin	11	34	116 ± 8	100 ± 4	87 ± 2	91 ± 6	90 ± 2	15	125
Nonsteroidal anti-inflammatory drugs	Celecoxib	13	38	118 ± 4	107 ± 4	93 ± 2	89 ± 1	85 ± 3	21	124
	Ketoprofen	7	20	126 ± 2	101 ± 4	86 ± 3	82 ± 5	87 ± 3	14	64
	Mefenamic acid	274	821	< 10	< 10	43 ± 6	54 ± 5	64 ± 2	16	51
	Meloxicam	2.8	8.5	123 ± 8	96 ± 2	81 ± 4	79 ± 1	78 ± 2	13	86
	Naproxen	15	46	125 ± 6	97 ± 6	79 ± 6	73 ± 10	70 ± 5	16	67
	Piroxicam	73	218	114 ± 2	91 ± 4	80 ± 4	77 ± 5	107 ± 2	12	100
Sulfonamides and Trimethoprim	Sulfacetamide	7.8	23	131 ± 6	90 ± 6	87 ± 2	77 ± 5	72 ± 2	13	28
	Sulfadiazine	41	123	114 ± 9	86 ± 2	83 ± 5	82 ± 1	74 ± 1	13	45
	Sulfadimethoxine	16	48	93 ± 5	80 ± 3	76 ± 5	70 ± 1	88 ± 7	19	94
	Sulfamerazine	21	63	116 ± 1	95 ± 1	82 ± 3	78 ± 3	86 ± 5	8	37
	Sulfamethazine	20	61	118 ± 1	98 ± 3	85 ± 2	83 ± 3	82 ± 5	9	52
	Sulfamethoxazole	31	93	113 ± 4	94 ± 1	83 ± 1	79 ± 1	77 ± 4	8	69
	Sulfamethoxypyridazine	24	72	91 ± 2	82 ± 4	81 ± 1	78 ± 1	80 ± 5	9	81
	Sulfapyridine	18	55	122 ± 9	90 ± 17	62 ± 24	83 ± 3	81 ± 2	27	30
	Sulfasalazine	40	121	144 ± 14	111 ± 10	89 ± 2	75 ± 3	79 ± 2	10	44
	Sulfathiazole	13	38	102 ± 2	85 ± 6	70 ± 4	72 ± 3	83 ± 3	15	60
	Trimethoprim	37	109	122 ± 14	98 ± 5	85 ± 3	84 ± 4	90 ± 9	16	27
Tetracyclines	Chlortetracycline	35	105	118 ± 5	94 ± 2	80 ± 5	80 ± 8	74 ± 9	19	142
	Doxycycline	59	178	121 ± 16	100 ± 6	73 ± 15	81 ± 12	100 ± 1	20	181
	Minocycline	70	210	93 ± 17	114 ± 7	75 ± 9	63 ± 3	67 ± 4	15	160
	Oxytetracycline	66	196	121 ± 7	96 ± 7	79 ± 2	79 ± 3	73 ± 4	11	150
	Tetracycline	72	215	102 ± 18	110 ± 13	69 ± 6	80 ± 21	80 ± 4	23	31
Throat Preparations	Flurbiprofen	54	163	52 ± 14	59 ± 14	56 ± 11	55 ± 16	70 ± 7	14	53
Topical products for joint and muscular pain	Diclofenac	23	69	91 ± 17	93 ± 6	74 ± 15	92 ± 19	64 ± 3	16	68
	Indomethacin	3.6	11	123 ± 4	105 ± 1	92 ± 1	87 ± 1	74 ± 10	19	56
	Nimesulide	1.9	5.6	101 ± 5	115 ± 3	112 ± 7	106 ± 3	74 ± 12	11	36

The calculated MLoDs are within the tens to hundreds of ng·g^−1^, albeit slightly higher than those reported by Bergé and Vulliet (2015) (QuEChERS, MLoD < 14 ng·g^−1^) and significantly higher than those reported by Montemurro et al. (2021) (Vortexing + SPE, MLoD 0.01–0.54 ng·g^−1^). The MLoD values achieved in the study by Montemurro et al. (2021) are significantly lower because of the concentration step during SPE, whereas the QuEChERS method dilutes samples during sample preparation. Moreover, it is crucial to acknowledge the distinction in our study, where a lower sample weight of lyophilized earthworm (0.1 g) was utilized, in contrast to other studies employing 0.25–0.5 g of sample weight (Bergé and Vulliet 2015; Montemurro et al. 2021). In alignment with OECD guidelines, our exposure experiments utilized earthworms with an average weight of 400 mg. As a result, individual lyophilized earthworms weighed approximately 100 mg. Additionally, method LoDs could be enhanced by adopting more sensitive instruments, as demonstrated in other studies. It is noteworthy that MLoDs are influenced by various factors, including the calculation method, which can vary by up to an order of magnitude (Kruve et al. 2015a), the ratio of sample weight to extraction solvent, preconcentration steps, recovery rates and matrix effects (Kruve et al. 2015b).

The recovery rates of PhACs were evaluated by spiking blank earthworm extracts at five concentration levels (250 ng·g^−1^, 500 ng·g^−1^, 1,000 ng·g^−1^, 2,000 ng·g^−1^, and 2,500 ng·g^−1^ of earthworm dry weight). Suboptimal RR values (below 10%) were identified solely at the lowest spike concentration (250 ng·g^−1^) for one of the 42 pharmaceuticals, which was attributable to the limited sensitivity of the mass spectrometry instrument, specifically for Mefenamic acid. However, an average RR exceeding 50% was attained for 40 of the 42 pharmaceuticals (excluding Erythromycin and Mefenamic acid), with an RSD% below 30%. These 40 PhACs exhibited an average RR across the entire concentration range of 88%, ranging from 54 to 102%. This performance aligns with the results achieved by Bergé and Vulliet (2015), both in terms of recovery rates and the number of validated compounds. It is noteworthy that Bergé and Vulliet (2015) primarily focused on hormones and steroids. Meanwhile, study by Montemurro et al. (2021) focused on wastewater-borne pollutants, with particular attention given to pharmaceuticals as a subset of these pollutants. In contrast, our study predominantly investigated veterinary antimicrobials (SA, FQs, TCs, MLs); hence, our method included different compounds.

Despite employing dSPE (C18/PSA) for the purification of earthworm extracts, notable matrix effects exceeding 30% were identified in the case of 35 out of 42 pharmaceuticals. Consequently, to address these observed significant matrix effects, a matrix-matched external calibration was prepared. These findings align with the results reported by Bergé and Vulliet (2015), where the QuEChers extraction method was employed. Similar matrix effects were also observed in study by Montemurro et al. (2021), which used extraction via vortex followed by subsequent SPE in both ESI + and ESI- modes.

### Sample analysis

Consequently, these validated methods were employed to investigate pharmaceutical uptake by both lettuce and earthworms in a soil environment in which the initial concentrations of PhACs in the soil were 1,000 ng·g^−1^ dw of each compound. A detailed and comprehensive description of these uptake experiments is provided in the Supplementary Information. Earthworms (N = 11) were sampled at the conclusion of a 21-day exposure period (refer to Results in Table S9), whereas lettuces (N = 5) were sampled after 28 days of exposure. The concentrations of PhACs in lettuce roots are detailed in Table S10, and those in lettuce leaves are provided in Table S11. Additionally, the degradation rates of PhACs in the soil during the two exposure experiments are outlined in Table S9 for earthworms and Table S10 for lettuce.

The concentrations of PhACs in earthworm tissue and lettuce displayed a wide range, ranging from below MLoD values to several thousands of ng·g^−1^ in earthworms, from the < MLoD to hundreds of ng·g^−1^ in lettuce roots, and from the < MLoD to dozens of ng·g^−1^ in lettuce leaves. The measured concentrations were consistent with values reported in relevant studies by Bergé and Vulliet (2015); Carter et al. (2014); Kinney et al. (2008); Kodešová et al. (2019); and Montemurro et al. (2021). In alignment with findings from studies by Keerthanan et al. (2020) and Stuchlíková Raisová et al. (2017) exploring the uptake of PhACs, lettuces exhibited higher concentrations of drugs in their roots than in leaves, attributed to limited translocation to the leaves. In addition, the detection frequencies of individual PhACs varied: in earthworms, ranging from 1/11 (9%) to 8/11 (73%); in lettuce roots, ranging from 0/5 (0%) to 5/5 (100%); and in lettuce leaves, ranging from 0/5 (0%) to 2/5 (40%).

As indicated in the study by Li et al. (2019), the concentrations of uptake PhACs are based on the contamination level of the soil environment. Furthermore, the uptake of PhACs is influenced by their physicochemical properties and various soil characteristics, including pH, organic matter content, and the presence of cations (Keerthanan et al. 2020; Kim et al. 2010; Sanford et al. 2009). It is essential to highlight that targeted analysis can solely detect the parent pharmaceuticals, potentially leading to an underestimation of environmental and health risks, as drugs taken up by organisms may undergo further metabolization and remain undetected (Keerthanan et al. 2020; Miller et al. 2016; Riemenschneider et al. 2016).

Throughout the exposure experiments, PhACs in the soil exhibited diverse degradation rates, varying among different pharmaceutical groups. The average degradation rates for soil from earthworm exposure experiments were as follows: SAs at 89%, FQs at 34%, TCs at 23%, MLs at 43%, beta blockers at 63%, and NSAIDs at 27%. In the lettuce uptake experiment, the degradation rates were as follows: SAs at 94%, FQs at 34%, TCs at 69%, MLs at 0%, beta blockers at 99%, and NSAIDs at 99%. Slight differences in PhAC degradation between these two experiments could be attributed to variations in the experimental setup, the presence of different organisms, and consequently, different uptake rates. Nevertheless, these degradation rates align with the known half-lives of veterinary antibiotics reported in the literature by Song and Guo (2014), with SAs exhibiting greater degradation than more persistent antibiotics such as FQs, TCs, and MLs.

Furthermore, Risk Quotients (RQs) were computed from PhAC soil concentrations at the conclusion of exposure experiments to assess the potential risk of antimicrobial resistance (AMR), as outlined in the Supplementary Information, “Introduction” section. RQs were determined when both predicted no effect concentration values and soil distribution coefficients (Kd) were available in the scientific literature. RQs were calculated within a range (MIN–MAX) for both earthworm-soil (Table S9) and lettuce-soil (Table S10) samples, as specific Kd values for our soil were not available. Therefore, a range of Kd values from the literature was utilized. The interpretation criteria for RQs were consistent with common practice: low risk when RQ < 0.1, medium risk when 0.1 < RQ < 1, and high risk for RQ > 1 (Deschamps et al. 2017; Sun et al. 2017). Moreover, RQs were notably influenced by the initial concentration of PhACs in the soil (in our experiments, 1,000 ng·g^−1^), although similar concentrations have been reported in soil (Cycoń et al. 2019; Pan and Chu 2017). Following 21 days (for earthworms) and 28 days (for lettuce), the results indicate that more persistent antimicrobials such as FQ (ciprofloxacin, enrofloxacin) and MLs (azithromycin, clarithromycin, erythromycin) present a significantly higher risk of AMR emergence (RQs >  > 1.0) compared with SAs (in our case, sulfamethoxazole with RQs < 1.0) in both lettuce and earthworm experiments. These results emphasize the multidisciplinary challenge posed by PhAC residues and resistance genes in the soil environment.

## Conclusion

In this study, we present optimized extraction methods for analyzing 42 pharmaceuticals in soil, lettuce leaves, and earthworms. These methods were validated by investigating parameters such as MLoD, MLoQ, linearity, recovery rate, precision, selectivity, and identity confirmation. Following the validation, exposure experiments confirmed pharmaceutical uptake by earthworms and lettuce, demonstrating the applicability of the methods in real-world scenarios for all three matrices. These methodologies hold the potential for applications such as identifying contamination hotspots, mapping affected areas, evaluating compliance with permissible limits (if established), and gauging the efficacy of diverse technologies in mitigating the presence of these micropollutants or reducing their uptake by plants and/or soil organisms. The proposed methods can be successfully employed to quantitatively monitor pharmaceutical residues in various environmental compartments. While essential, it is imperative to complement these environmental analyses with PCR analyses of resistance genes or, at the very least, estimate RQs to assess the emergence of antimicrobial resistance. This comprehensive approach allows for a thorough assessment of environmental pollution, health risks, and the effectiveness of removal technologies.

### Supplementary Information

Below is the link to the electronic supplementary material.

Supplementary file1 (DOCX 1370 KB)

## Data Availability

The data that support the findings of this study are available from the corresponding author, Jan Fučík, upon reasonable request.

## References

[CR1] Ajibola AS, Tisler S, Zwiener C (2020). Simultaneous determination of multiclass antibiotics in sewage sludge based on QuEChERS extraction and liquid chromatography-tandem mass spectrometry. Anal Methods.

[CR2] Albero B, Sánchez-Brunete C, García-Valcárcel AI, Pérez RA, Tadeo JL (2015). Ultrasound-assisted extraction of emerging contaminants from environmental samples. TrAC, Trends Anal Chem.

[CR3] Albero B, Tadeo JL, Pérez RA (2019). Ultrasound-assisted extraction of organic contaminants. TrAC, Trends Anal Chem.

[CR4] Aznar R, Sánchez-Brunete C, Albero B, Rodríguez JA, Tadeo JL (2013). Occurrence and analysis of selected pharmaceutical compounds in soil from Spanish agricultural fields. Environ Sci Pollut Res.

[CR5] Bair DA, Mukome FND, Popova IE, Ogunyoku TA, Jefferson A, Wang D, Hafner SC, Young TM, Parikh SJ (2016). Sorption of Pharmaceuticals, Heavy Metals, and Herbicides to Biochar in the Presence of Biosolids. J Environ Qual.

[CR6] Bartrons M, Peñuelas J (2017). Pharmaceuticals and Personal-Care Products in Plants. Trends Plant Sci.

[CR7] Beek T, Weber F, Bergmann A, Hickmann S, Ebert I, Hein A, Küster A (2016). Pharmaceuticals in the environment—Global occurrences and perspectives. Environ Toxic Chem.

[CR8] Bergé A, Vulliet E (2015). Development of a method for the analysis of hormones and pharmaceuticals in earthworms by quick, easy, cheap, effective, rugged and safe (QuEChERS) extraction followed by liquid chromatography-tandem mass spectrometry (LC-MS/MS). Anal Bioanal Chem.

[CR9] Bian K, Liu Y, Wang Z, Zhou T, Song X, Zhang F, He L (2015). Determination of multi-class antimicrobial residues in soil by liquid chromatography-tandem mass spectrometry. RSC Adv.

[CR10] Bielińska AB, Kumirska J, Borecka M, Caban M, Paszkiewicz M, Pazdro K, Stepnowski P (2016). Selected analytical challenges in the determination of pharmaceuticals in drinking/marine waters and soil/sediment samples. J Pharm Biomed Anal.

[CR11] Caldú AA, Diaz-Cruz MS (2021). Development of a QuEChERS-based method for the analysis of pharmaceuticals and personal care products in lettuces grown in field-scale agricultural plots irrigated with reclaimed water. Talanta.

[CR12] Carballo EM, González-Barreiro C, Scharf S, Gans O (2007). Environmental monitoring study of selected veterinary antibiotics in animal manure and soils in Austria. Environ Pollut.

[CR13] Carter LJ, Garman CD, Ryan J, Dowle A, Bergström E, Thomas-Oates J, Boxall ABA (2014). Fate and Uptake of Pharmaceuticals in Soil-Earthworm Systems. Environ Sci Technol.

[CR14] Carvalho PN, Zhang Y, Lyu T, Arias CA, Bester K, Brix H (2018). Methodologies for the analysis of pesticides and pharmaceuticals in sediments and plant tissue. Anal Methods.

[CR15] Carvalho PN (2020) Constructed Wetlands and Phytoremediation as a Tool for Pharmaceutical Removal. The Handbook of Environmental Chemistry.10.1007/698_2020_624

[CR16] Celiz MD, Tso J, Aga DS (2009). Pharmaceutical metabolites in the environment: Analytical challenges and ecological risks. Environ Toxic Chem.

[CR17] Chen J, He L-X, Cheng Y-X, Ye P, Wu D-L, Fang Z-Q, Li J, Ying G-G (2020). Trace analysis of 28 antibiotics in plant tissues (root, stem, leaf and seed) by optimized QuEChERS pretreatment with UHPLC-MS/MS detection. J Chromatogr B.

[CR18] Chitescu CL, Oosterink E, de Jong J, Stolker AAM (Linda) (2012). Ultrasonic or accelerated solvent extraction followed by U-HPLC-high mass accuracy MS for screening of pharmaceuticals and fungicides in soil and plant samples. Talanta.

[CR19] Chuang Y-H, Zhang Y, Zhang W, Boyd SA, Li H (2015). Comparison of accelerated solvent extraction and quick, easy, cheap, effective, rugged and safe method for extraction and determination of pharmaceuticals in vegetables. J Chromatogr A.

[CR20] Chung HS, Lee Y-J, Rahman MdM, Abd El-Aty AM, Lee HS, Kabir MdH, Kim SW, Park B-J, Kim J-E, Hacımüftüoğlu F, Nahar N, Shin H-C, Shim J-H (2017). Uptake of the veterinary antibiotics chlortetracycline, enrofloxacin, and sulphathiazole from soil by radish. Sci Total Environ.

[CR21] Cycoń M, Mrozik A, Piotrowska-Seget Z (2019) Antibiotics in the Soil Environment—Degradation and Their Impact on Microbial Activity and Diversity. Front Microbiol 10. 10.3389/fmicb.2019.00338 10.3389/fmicb.2019.00338 PMC6418018 30906284

[CR22] Deschamps MB, Leang S, Bernet N, Daudin J-J, Nélieu S (2014). Multi-residue analysis of pharmaceuticals in aqueous environmental samples by online solid-phase extraction–ultra-high-performance liquid chromatography-tandem mass spectrometry: Optimisation and matrix effects reduction by quick, easy, cheap, effective, rugged and safe extraction. J Chromatogr A.

[CR23] Deschamps BM, Ferhi S, Bernet N et al (2017) Fate and impacts of pharmaceuticals and personal care products after repeated applications of organic waste products in long-term field experiments. Sci Total Environ 607–608:271–280. 10.1016/j.scitotenv.2017.06.240 10.1016/j.scitotenv.2017.06.240 28692897

[CR24] Duarte RMBO, Matos JTV, Senesi N (2018) Organic Pollutants in Soils. Soil Pollut 103–126. 10.1016/B978-0-12-849873-6.00005-4

[CR25] Emhofer L, Himmelsbach M, Buchberger W, Klampfl CW (2017). High-performance liquid chromatography – mass spectrometry analysis of the parent drugs and their metabolites in extracts from cress (Lepidium sativum) grown hydroponically in water containing four non-steroidal anti-inflammatory drugs. J Chromatogr A.

[CR26] European Medicines Agency (2023) Sales of veterinary antimicrobial agents in 31 European countries in 2022: trends from 2010 to 2022 : thirteenth ESVAC report. Publications Office, LU. 10.2809/895656

[CR27] Ferhi S, Bourdat-Deschamps M, Daudin J-J, Houot S, Nélieu S (2016). Factors influencing the extraction of pharmaceuticals from sewage sludge and soil: an experimental design approach. Anal Bioanal Chem.

[CR28] Ferrero PG, Borova V, Dasenaki ME, Τhomaidis ΝS (2015). Simultaneous determination of 148 pharmaceuticals and illicit drugs in sewage sludge based on ultrasound-assisted extraction and liquid chromatography–tandem mass spectrometry. Anal Bioanal Chem.

[CR29] Gaffney VJ, Cardoso VV, Cardoso E, Teixeira AP, Martins J, Benoliel MJ, Almeida CMM (2017). Occurrence and behaviour of pharmaceutical compounds in a Portuguese wastewater treatment plant: Removal efficiency through conventional treatment processes. Environ Sci Pollut Res.

[CR30] Gao Q, Dong Q, Wu L, Yang Y, Hale L, Qin Z, Xie C, Zhang Q, Van Nostrand JD, Zhou J (2020). Environmental antibiotics drives the genetic functions of resistome dynamics. Environ Int.

[CR31] Golovko O, Koba O, Kodesova R, Fedorova G, Kumar V, Grabic R (2016). Development of fast and robust multiresidual LC-MS/MS method for determination of pharmaceuticals in soils. Environ Sci Pollut Res.

[CR32] Gros M, Mas-Pla J, Boy-Roura M, Geli I, Domingo F, Petrović M (2019). Veterinary pharmaceuticals and antibiotics in manure and slurry and their fate in amended agricultural soils: Findings from an experimental field site (Baix Empordà, NE Catalonia). Sci Total Environ.

[CR33] Gworek B, Kijeńska M, Wrzosek J, Graniewska M (2021) Pharmaceuticals in the Soil and Plant Environment: a Review. Water Air Soil Pollut 232. 10.1007/s11270-020-04954-8

[CR34] Hang L, Zhao Y, Liu C, Yu Y, He Y, Xu J, Lu Z (2021). Determine Multiple Classes of Veterinary Antibiotics in Soil: Comparing Dispersive and Solid-Phase Extraction for Sample Cleanup. Chromatographia.

[CR35] He Z, Wang Y, Xu Y, Liu X (2018). Determination of Antibiotics in Vegetables Using QuEChERS-Based Method and Liquid Chromatography-Quadrupole Linear Ion Trap Mass Spectrometry. Food Anal Methods.

[CR36] Hernandez JCS (2020) Vermiremediation of Pharmaceutical-Contaminated Soils and Organic Amendments. The Handbook of Environmental Chemistry.10.1007/698_2020_625

[CR37] Ho YB, Zakaria MP, Latif PA, Saari N (2013). Degradation of veterinary antibiotics and hormone during broiler manure composting. Biores Technol.

[CR38] Hu W, Ma L, Guo C, Sha J, Zhu X, Wang Y (2012). Simultaneous extraction and determination of fluoroquinolones, tetracyclines and sulfonamides antibiotics in soils using optimised solid phase extraction chromatography-tandem mass spectrometry. Int J Environ Anal Chem.

[CR39] Hu S, Zhang Y, Shen G, Zhang H, Yuan Z, Zhang W (2019). Adsorption/desorption behavior and mechanisms of sulfadiazine and sulfamethoxazole in agricultural soil systems. Soil Till Res.

[CR40] Huang Y, Cheng M, Li W, Wu L, Chen Y, Luo Y, Christie P, Zhang H (2013). Simultaneous extraction of four classes of antibiotics in soil, manure and sewage sludge and analysis by liquid chromatography-tandem mass spectrometry with the isotope-labelled internal standard method. Anal Methods.

[CR41] Huang Q, Zhang J, Xiong S, Peng X, Wei G, Liu L, Sun X, Li L (2018). Development of ultrasound-assisted extraction of commonly used azole antifungals in soils. Anal Methods.

[CR42] Keerthanan S, Jayasinghe C, Biswas JK, Vithanage M (2020). Pharmaceutical and Personal Care Products (PPCPs) in the environment: Plant uptake, translocation, bioaccumulation, and human health risks. Crit Rev Environ Sci Technol.

[CR43] Kim K-R, Owens G, Kwon S-I, So K-H, Lee D-B, Ok YS (2010). Occurrence and Environmental Fate of Veterinary Antibiotics in the Terrestrial Environment. Water Air Soil Pollut.

[CR44] Kim L, Lee D, Cho H-K, Choi S-D (2019). Review of the QuEChERS method for the analysis of organic pollutants: Persistent organic pollutants, polycyclic aromatic hydrocarbons, and pharmaceuticals. Trends Environ Anal Chem.

[CR45] Kinney CA, Furlong ET, Kolpin DW, Burkhardt MR, Zaugg SD, Werner SL, Bossio JP, Benotti MJ (2008). Bioaccumulation of Pharmaceuticals and Other Anthropogenic Waste Indicators in Earthworms from Agricultural Soil Amended With Biosolid or Swine Manure. Environ Sci Technol.

[CR46] Kodešová R, Klement A, Golovko O, Fér M, Nikodem A, Kočárek M, Grabic R (2019). Root uptake of atenolol, sulfamethoxazole and carbamazepine, and their transformation in three soils and four plants. Environ Sci Pollut Res.

[CR47] Kruve A, Rebane R, Kipper K, Oldekop M-L, Evard H, Herodes K, Ravio P, Leito I (2015). Tutorial review on validation of liquid chromatography–mass spectrometry methods: Part I. Anal Chim Acta.

[CR48] Kruve A, Rebane R, Kipper K, Oldekop M-L, Evard H, Herodes K, Ravio P, Leito I (2015). Tutorial review on validation of liquid chromatography–mass spectrometry methods: Part II. Anal Chim Acta.

[CR49] Kuppusamy S, Kakarla D, Venkateswarlu K, Megharaj M, Yoon Y-E, Lee YB (2018). Veterinary antibiotics (VAs) contamination as a global agro-ecological issue: A critical view. Agr Ecosyst Environ.

[CR50] Lawal A, Wong RCS, Tan GH, Abdulra’uf LB, Alsharif AMA (2018). Recent Modifications and Validation of QuEChERS-dSPE Coupled to LC–MS and GC–MS Instruments for Determination of Pesticide/Agrochemical Residues in Fruits and Vegetables: Review. J Chromatogr Sci.

[CR51] Li Y, Sallach JB, Zhang W, Boyd SA, Li H (2019). Insight into the distribution of pharmaceuticals in soil-water-plant systems. Water Res.

[CR52] Li Y, Lian J, Wu B, Zou H, Tan SK (2020). Phytoremediation of pharmaceutical-contaminated wastewater: Insights into rhizobacterial dynamics related to pollutant degradation mechanisms during plant life cycle. Chemosphere.

[CR53] Llompart M, Celeiro M, Dagnac T (2019). Microwave-assisted extraction of pharmaceuticals, personal care products and industrial contaminants in the environment. TrAC, Trends Anal Chem.

[CR54] Maia AS, Ribeiro AR, Amorim CL, Barreiro JC, Cass QB, Castro PML, Tiritan ME (2014). Degradation of fluoroquinolone antibiotics and identification of metabolites/transformation products by liquid chromatography–tandem mass spectrometry. J Chromatogr A.

[CR55] Manasfi R, Labad F, Montemurro N (2020) Development of Methods for the Determination of PhACs in Soil/Earthworm/Crop System Irrigated with Reclaimed Water. The Handbook of Environmental Chemistry. 10.1007/698_2020_650

[CR56] Miller EL, Nason SL, Karthikeyan KG, Pedersen JA (2016). Root Uptake of Pharmaceuticals and Personal Care Product Ingredients. Environ Sci Technol.

[CR57] Mirzaei R, Yunesian M, Nasseri S, Gholami M, Jalilzadeh E, Shoeibi S, Bidshahi HS, Mesdaghinia A (2017) An optimized SPE-LC-MS/MS method for antibiotics residue analysis in ground, surface and treated water samples by response surface methodology- central composite design. J Environ Health Sci Eng 15. 10.1186/s40201-017-0282-2 10.1186/s40201-017-0282-2 PMC5646162 29075502

[CR58] Montemurro N, Postigo C, Lonigro A, Perez S, Barceló D (2017). Development and validation of an analytical method based on liquid chromatography–tandem mass spectrometry detection for the simultaneous determination of 13 relevant wastewater-derived contaminants in lettuce. Anal Bioanal Chem.

[CR59] Montemurro N, Joedicke J, Pérez S (2021). Development and application of a QuEChERS method with liquid chromatography-quadrupole time of flight-mass spectrometry for the determination of 50 wastewater-borne pollutants in earthworms exposed through treated wastewater. Chemosphere.

[CR60] Mullen RA, Wigginton KR, Noe-Hays A, Nace K, Love NG, Bott CB, Aga DS (2017). Optimizing extraction and analysis of pharmaceuticals in human urine, struvite, food crops, soil, and lysimeter water by liquid chromatography-tandem mass spectrometry. Anal Methods.

[CR61] Pan M, Chu LM (2017). Fate of antibiotics in soil and their uptake by edible crops. Sci Total Environ.

[CR62] Pereira LC, Souza AO, Bernardes MFF, Pazin M, Tasso MJ, Pereira PH, Dorta DJ (2015). A perspective on the potential risks of emerging contaminants to human and environmental health. Environ Sci Pollut Res.

[CR63] Pino MR, Muñiz S, Val J, Navarro E (2016). Phytotoxicity of 15 common pharmaceuticals on the germination of Lactuca sativa and photosynthesis of Chlamydomonas reinhardtii. Environ Sci Pollut Res.

[CR64] Rakonjac N, Seatm S, Wipfler L, Roex E, Kros H (2022). Emission estimation and prioritization of veterinary pharmaceuticals in manure slurries applied to soil. Sci Total Environ.

[CR65] Riemenschneider C, Al-Raggad M, Moeder M, Seiwert B, Salameh E, Reemtsma T (2016). Pharmaceuticals, Their Metabolites, and Other Polar Pollutants in Field-Grown Vegetables Irrigated with Treated Municipal Wastewater. J Agric Food Chem.

[CR66] Riva F, Zuccato E, Pacciani C, Colombo A, Castiglioni S (2021). A multi-residue analytical method for extraction and analysis of pharmaceuticals and other selected emerging contaminants in sewage sludge. Anal Methods.

[CR67] Rodrigues JA, Silva S, Cardoso VV, Benoliel MJ, Almeida CMM (2023). Different approaches for estimation of the expanded uncertainty of an analytical method developed for determining pharmaceutical active compounds in wastewater using solid-phase extraction and a liquid chromatography coupled with tandem mass spectrometry method. Anal Methods.

[CR68] Safont DF, Gracia-Marín E, Ibáñez M, Pitarch E, Hernández F (2023). Analytical key issues and challenges in the LC-MS/MS determination of antibiotics in wastewater. Anal Chim Acta.

[CR69] Sallach JB, Snow D, Hodges L, Li X, Bartelt-Hunt S (2015). Development and comparison of four methods for the extraction of antibiotics from a vegetative matrix. Environ Toxic Chem.

[CR70] Sanford JC, Mackie RI, Koike S, Krapac IG, Lin Y, Yannarell AC, Maxwell S, Aminov RI (2009). Fate and Transport of Antibiotic Residues and Antibiotic Resistance Genes following Land Application of Manure Waste. J Environ Qual.

[CR71] Silva JJ, Silva BF, Stradiotto NR, Petrovic M, Gago-Ferrero P, Gros M (2020). Pressurized Liquid Extraction (PLE) and QuEChERS evaluation for the analysis of antibiotics in agricultural soils. MethodsX.

[CR72] Solliec M, Roy-Lachapelle A, Gasser M-O, Coté C, Généreux M, Sauvé S (2016). Fractionation and analysis of veterinary antibiotics and their related degradation products in agricultural soils and drainage waters following swine manure amendment. Sci Total Environ.

[CR73] Song W, Guo M (2014) Residual Veterinary Pharmaceuticals in Animal Manures and Their Environmental Behaviors in Soils. Applied Manure and Nutrient Chemistry for Sustainable Agriculture and Environment 23–52. 10.1007/978-94-017-8807-6_2

[CR74] Souza MP, Rizzetti TM, Francesquett JZ, Prestes OD, Zanella R (2018). Bar adsorptive microextraction (BAμE) with a polymeric sorbent for the determination of emerging contaminants in water samples by ultra-high performance liquid chromatography with tandem mass spectrometry. Anal Methods.

[CR75] Stuchlíková Raisová L, Podlipná R, Szotáková B, Syslová E, Skálová L (2017). Evaluation of drug uptake and deactivation in plant: Fate of albendazole in ribwort plantain (Plantago laceolata) cells and regenerants. Ecotoxicol Environ Saf.

[CR76] Sun J, Zeng Q, Tsang DCW, Zhu LZ, Li XD (2017). Antibiotics in the agricultural soils from the Yangtze River Delta, China. Chemosphere.

[CR77] Tadić Đ, Gramblicka M, Mistrik R, Bayona JM (2022). Systematic identification of trimethoprim metabolites in lettuce. Anal Bioanal Chem.

[CR78] Tao Y, Xing Y, Jing J, Yu P, He M, Zhang J, Chen L, Jia C, Zhao E (2022). Insight into the uptake, accumulation, and metabolism of the fungicide phenamacril in lettuce (Lactuca sativa L.) and radish (Raphanus sativus L.). Environ Pollut.

[CR79] Tetzner NF, Maniero MG, Rodrigues-Silva C, Rath S (2016). On-line solid phase extraction-ultra high performance liquid chromatography-tandem mass spectrometry as a powerful technique for the determination of sulfonamide residues in soils. J Chromatogr A.

[CR80] Tian R, Zhang R, Uddin M, Qiao X, Chen J, Gu G (2019). Uptake and metabolism of clarithromycin and sulfadiazine in lettuce. Environ Pollut.

[CR81] Turiel E, Martín-Esteban A, Tadeo JL (2006). Multiresidue analysis of quinolones and fluoroquinolones in soil by ultrasonic-assisted extraction in small columns and HPLC-UV. Anal Chim Acta.

[CR82] Vazquez-Roig P, Segarra R, Blasco C, Andreu V, Picó Y (2010). Determination of pharmaceuticals in soils and sediments by pressurized liquid extraction and liquid chromatography tandem mass spectrometry. J Chromatogr A.

[CR83] Xu D, Xiao Y, Pan H, Mei Y (2019). Toxic effects of tetracycline and its degradation products on freshwater green algae. Ecotoxicol Environ Saf.

[CR84] Xu Y, Yu X, Xu B, Peng D, Guo X (2021). Sorption of pharmaceuticals and personal care products on soil and soil components: Influencing factors and mechanisms. Sci Total Environ.

[CR85] Yu Z, Yediler A, Yang M, Schulte-Hostede S (2012). Leaching behavior of enrofloxacin in three different soils and the influence of a surfactant on its mobility. J Environ Sci.

[CR86] Yu X, Liu H, Pu C, Chen J, Sun Y, Hu L (2017). Determination of multiple antibiotics in leafy vegetables using QuEChERS–UHPLC–MS/MS. J Sep Sci.

[CR87] Zhang Z, Zhao J, Yu C, Dong S, Zhang D, Yu R, Wang C, Liu Y (2015). Evaluation of aerobic co-composting of penicillin fermentation fungi residue with pig manure on penicillin degradation, microbial population dynamics and composting maturity. Biores Technol.

[CR88] Zhang X, Zhao H, Du J, Qu Y, Shen C, Tan F, Chen J, Quan X (2017). Occurrence, removal, and risk assessment of antibiotics in 12 wastewater treatment plants from Dalian, China. Environ Sci Pollut Res.

[CR89] Zhang Y, Lin L, Li Y, Zeng Q, Guo S, Nkinahamira F, Yu C-P, Sun Q (2021). Determination of 38 pharmaceuticals and personal care products in water by lyophilization combined with liquid chromatography-tandem mass spectrometry. Anal Methods.

