# Supplementary material for: Optimization and validation of multiresidual extraction methods for pharmaceuticals in Soil, Lettuce, and Earthworms
Source: Environ Sci Pollut Res Int. 2024 Apr 27;31(22):33120–40. doi: 10.1007/s11356-024-33492-7 (PMC11133184; doi:10.1007/s11356-024-33492-7)
Supplement: Supplementary file 1 — Supplementary file1 (DOCX 1370 KB) [file 11356_2024_33492_MOESM1_ESM.docx]

**Supplementary Information for**

**Optimization and Validation of Multiresidual Extraction Methods**

**for Pharmaceuticals in Soil, Lettuce, and Earthworms**

Ludmila Mravcová^1^, Anna Amrichová^1^, Jitka Navrkalová^1^, Marie Hamplová^1^, Marian Sedlář^2^, Helena Zlámalová Gargošová^1^, Jan Fučík^1,*^

*^1^ Institute of Environmental Chemistry, Faculty of Chemistry, Brno University of Technology, Purkyňova 118, 612 00 Brno, Czech Republic*

*^2^* *CEITEC Brno University of Technology, Purkyňova 656/123, 612 00 Brno, Czech Republic*

*Corresponding author: [xcfucikj@vutbr.cz](mailto:xcfucikj@vutbr.cz)**Table of Contents**

**Section 1.** Assessing Potential Environmental Risks Associated with the Emergence of Antimicrobial Resistance

**Table S1.** List of Antimicrobials according to priority list for Antimicrobial Resistance Risk

**Table S2.** Overview of extraction methods for parent pharmaceuticals in soil samples

**Table S3.** Overview of extraction methods for parent pharmaceuticals in lettuce and vegetable samples

**Table S4.** Overview of extraction methods for parent pharmaceuticals in earthworms

**Table S5.** Physico-chemical properties of pharmaceuticals

**Table S6.** Physico-chemical properties of soil

**Fig. S1** Extraction scheme for extraction of pharmaceuticals from soil samples

**Fig. S2** QuEChERS extraction scheme for extraction of pharmaceuticals from lettuce samples

**Fig. S3** QuEChERS extraction scheme for extraction of pharmaceuticals from earthworm samples

**Table S7.** MRM transitions of selected pharmaceuticals

**Section 2.** Description of the Exposure Experiments

**Table S8.** Overview of different EM used for extraction of pharmaceuticals from soil

**Section 3.** Preparation of the Buffer Solution for the Extraction Media

**Fig. S4** Method optimization for extraction of PhACs from soil using EM1-EM15

**Fig. S5** Method optimization for extraction of PhACs from soil (influence of organic solvent)

**Fig. S6** Method optimization for extraction of PhACs from soil (infuence of pH)

**Fig. S7** Method optimization for extraction of PhACs from soil (infuence of extraction mechanism)

**Fig. S8** Method optimization for extraction of PhACs from soil (infuence of EDTA)

**Fig. S9** Method optimization for extraction of PhACs from soil (infuence of extraction temperature)

**Fig. S10** Method optimization for extraction of PhACs from lettuce leaves (different EM)

**Fig. S11** Method optimization for extraction of PhACs from lettuce leaves (influence of pH)

**Fig. S12** Method optimization for extraction of PhACs from lettuce leaves (influence of EDTA)

**Fig. S13** Method optimization for extraction of PhACs from lettuce leaves (influence of dSPE sorbents)

**Fig. S14** Method optimization for extraction of PhACs from earthworms (influence of organic solvents)

**Fig. S15** Method optimization for extraction of PhACs from earthworms (influence of pH)

**Fig. S16** Method optimization for extraction of PhACs from earthworms (influence of EDTA)

**Fig. S17** Method optimization for extraction of PhACs from earthworms (influence of dSPE sorbents)

**Table S9.** Pharmaceutical concentration in Earthworms

**Table S10.** Pharmaceutical concentration in Lettuce Roots

**Table S11.** Pharmaceutical concentration in Lettuce Leaves

**Section 1.** Assessing Potential Environmental Risks Associated with the Emergence of Antimicrobial Resistance

**Table S1.** List of Antimicrobials according to priority list for Antimicrobial Resistance Risk (AMR Alliance 2023; Gravesen et al. 2020; Harrower et al. 2021; Meng et al. 2023; Pan et al. 2016; Sanford et al. 2009; Rodríguez-López et al. 2022; Rodríguez-López et al. 2023; Tang et al. 2019; Thiele‐Bruhn 2003; WHO collective 2018)

| Antimicrobial Class | | Criterion  or Prioritization factor | | | | | Examples  of antimicrobial  agents within  this study | PNEC-MIC  water  [µg/L] | Kd  [L∙kg^-1^] | PNEC-MIC  soil  [µg/g]  [**calc. Eq 1**] |
| --- | --- | --- | --- | --- | --- | --- | --- | --- | --- | --- |
|  |  | C1 | C2 | P1 | P2 | P3 |  |  |  |  |
| Critically  Important  Antimicrobials | Macrolides | ✔ | ✔ | ✔ | ✔ | ✔ | Azithromycin | 0.25 | 37-347 | 9.3-86.8 |
|  |  |  |  |  |  |  | Clarithromycin | 0.25 | 2.5-10.5 | 0.6-2.6 |
|  |  |  |  |  |  |  | Erythromycin | 1 | 67.6-337 | 67.6-337 |
|  |  |  |  |  |  |  | Roxithromycin | 1 | 667-2,392 | 667-2,392 |
|  | Quinolones | ✔ | ✔ | ✔ | ✔ | ✔ | Ciprofloxacin | 0.06 | 427-430 | 25.6-25.8 |
|  |  |  |  |  |  |  | Enrofloxacin | 0.06 | 260-6,310 | 15.6-379 |
|  |  |  |  |  |  |  | Moxifloxacin | 0.13 | 3,018 | 392 |
|  |  |  |  |  |  |  | Norlofxacin | 0.5 | 591-5,791 | 296-2,896 |
|  |  |  |  |  |  |  | Ofloxacin | 0.5 | 309-4,325 | 155-2,163 |
|  |  |  |  |  |  |  | Pefloxacin | 8 | N.D. | N.D. |
| Highly  Important  Antimicrobial | Sufonamides | **×** | ✔ | **×** | **×** | **×** | Sulfacetamide | N.D. | N.D. | N.D. |
|  |  |  |  |  |  |  | Sulfadiazine | N.D. | 2.5 | N.D. |
|  |  |  |  |  |  |  | Sulfadimethoxine | N.D. | 2.3-10 | N.D. |
|  |  |  |  |  |  |  | Sulfamerazine | N.D. | N.D: | N.D. |
|  |  |  |  |  |  |  | Sulfamethazine | N.D. | 0.6-3.1 | N.D. |
|  |  |  |  |  |  |  | Sulfamethoxazole | 16 | 8.1-59.4 | 130-950 |
|  |  |  |  |  |  |  | Sulfamethoxypyridazine | N.D. | N.D. | N.D. |
|  |  |  |  |  |  |  | Sulfapyridine | N.D. | 1.6-7.4 | N.D. |
|  |  |  |  |  |  |  | Sulfasalazine | N.D. | N.D. | N.D. |
|  |  |  |  |  |  |  | Sulfathiazole | N.D. | 3.0-4.9 | N.D. |
|  |  |  |  |  |  |  | Trimethoprim | 0.5 | 10.0-104.6 | 5.0-52.3 |
|  | Tetracyclines | ✔ | **×** | **×** | **×** | **×** | Chlortetracycline | N.D. | 282-2,608 | N.D. |
|  |  |  |  |  |  |  | Doxycycline | 2 | 431-11,908 | 862-23,816 |
|  |  |  |  |  |  |  | Minocycline | 1 | N.D. | N.D. |
|  |  |  |  |  |  |  | Oxytetracycline | 0.5 | 417-1,026 | 209-513 |
|  |  |  |  |  |  |  | Tetracycline | 1 | 198-1,620 | 198-1,620 |

Table S1 utilizes the prioritization factors outlined by the World Health Organization (WHO) to establish a priority list for assessing antimicrobial resistance risk (WHO collective 2018). **Criterion 1 (C1):** The antimicrobial class is the sole, or one of limited available therapies, to treat serious bacterial infections in people. **Criterion 2 (C2):** The antimicrobial class is used to treat infections in people caused by either: (1) bacteria that may be transmitted to humans from nonhuman sources, or (2) bacteria that may acquire resistance genes from nonhuman sources. **Prioritization factor 1 (P1):** Large number of people in the community or in certain highrisk populations (e.g. patients with serious infections in health care settings), who are affected by diseases for which there are very limited antimicrobial choices. **Prioritization factor 2 (P2):** High frequency of use of the antimicrobial class for any indication in human medicine or in certain high-risk groups (e.g. patients with serious infections in health care settings), since use may favour selection of resistance. **Prioritization factor 3 (P3):** The antimicrobial class is used to treat infections in people for which there is already extensive evidence of transmission of resistant bacteria (e.g. nontyphoidal *Salmonella spp.* and *Campylobacter spp.*) or resistance genes (high for *E. coli* and *Enterococcus spp.*) from non-human sources (WHO collective 2018).

Furthermore, the PNEC-MIC-water values (Predicted No-Effect Concentration values) presented in Table S1 were derived from the AMR Alliance (2023), offering insights into the potential risk of antimicrobial resistance in aquatic environments. Subsequently, these values underwent recalculation, as previously conducted in the study by Fučík et al. (2024), utilizing Eq. 1 to predict PNEC-MIC-soil values. To accomplish this, distribution coefficients (Kd) were sourced from relevant studies (Gravesen et al. 2020; Harrower et al. 2021; Meng et al. 2023; Pan et al. 2016; Sanford et al. 2009; Rodríguez-López et al. 2022; Rodríguez-López et al. 2023; Tang et al. 2019; Thiele‐Bruhn 2003), acknowledging it dependence on soil physicochemical properties. This led to the determination of ranges for both Kd and PNEC-MIC-soil values, aiding in the estimation of potential risks associated with antimicrobial resistance in terrestrial environments. Additionally, RQ (Risk Quotient) values were calculated as the ratio of measured environmental concentrations (MEC) to predicted no-effect concentrations (PNEC), based on the results obtained from our laboratory experiments (Table S9 and Table S10).

PNEC_soil_ = PNEC_water_ ∙ Kd (1)

where PNEC_soil_ is the predicted no-effect concentration in soil, PNEC_water_ is the predicted no-effect concentration, and Kd is the distribution coefficient for a given compound in the soil environment.

**Table S2.** Overview of extraction methods for parent pharmaceuticals in soil samples (FQs – Fluoroquinolones, TCs – Tetracyclines, SAs – Sulfonamides, MLs – Macrolides, NSAIDs – Non-Steroidal Anti-Inflammatory Drugs, LAs – Lincosamides, PMs -Pleuromutilins, UAE – Ultrasound Assisted Extraction; SPE – Solid Phase Extraction; SLE – Solid-Liquid Extraction, MAE – Microwave Assisted Extraction)

| **Analytes (No. of compounds)** | **Sample Weight** | **Extraction**  **method** | **Extraction Solvent** | **No. of extractions** | **RR [%]** | **LoD^[[1]](#footnote-1)^**  **[ng∙g^-1^]** | **Reference** |
| --- | --- | --- | --- | --- | --- | --- | --- |
| FQs (1) | 1.0 g dw | UAE+SPE | 0.2 g of EDTA + 5 mL of mixture (acetonitrile:buffer pH 3; 50:50, v/v) | 4 | 70-80 | - | Yu et al. (2012) |
| TCs, SAs, FQs, MLs (17) | 2.0 g dw | UAE+SPE | 20 mL of mixture (EDTA-Sodium Phosphate Buffer with acetonitrile:Mg(NO_3_)_2_-NH_3_∙H_2_O; 3:1, v/v) | 3 | 52.8-137.5 | 0.5-14.8 | Huang et al. (2013) |
| SAs (7) | 2.0 g dw | SLE+online-SPE | 10 mL of the mixture (acetronitrile:water; 1:1, v/v) | 1 | 70.2–99.9 | 0.5 | Tetzner et al. (2016) |
| MLs, TCs, FQs, SAs (34) | 1.0 g dw | *Vortexing+SPE* | 5 mL of mixture (acetonitrile:methanol; 1:1, v/v) + 5 mL of EDTA-McIlvaine buffer (pH4) | 2 | 47-103 | 0.007-1.4 | Hang et al. (2021) |
| Cephalosporins, FQs, LAs, MLs, Penicillins, SAs, TCs, etc. (31) | 1.0 g dw | QuEChERS | 5 mL of citrate-phosphate buffer (pH 7) + 6 mL of acetonitrile | 1 | 11.3-152.4 | 0.03-3.1 | Silva et al. (2020) |
| NSAIDs, Cardiovascular drugs, MLs, SAs, Antidepressants, Lipid regulators, Antidiabetics, etc. (44) | 2.0 g dw | UAE | 2 mL of acetonitrile + 2 mL of water (with 0.1 % formic acid);  2^nd^ extraction with 4 mL of with 0.1 % formic acid in mixture (acetonitrile:2-propanol:water; 3:3:4, v/v/v) | 2 | 55-135 | 0.6-9.4 | Golovko et al. (2016) |
| FQs, SAs, MLs, NSAIDs (14) | 5.0 g dw | QuEChERS+online-SPE | 6 mL of McIlvaine buffer (pH 3, containing 0.12 M EDTA) + 15 mL of acetonitrile | 1 | 60-115 | 0.1-20 | Ferhi et al. (2016) |
| MLs, TCs, Beta-blockers, Antidepressants, NSAIDs, Antifungals (8) | 5.0 g dw | QuEChERS | 10 mL of water + 15 mL of acetonitrile + 2 mL of methanol | 1 | 79.5-92.8 | 10-18.4 | Mastro et al.  (2022) |
| NSAIDs, Antiepileptics, Beta-blockers, Hormones, Antidepressants (20) | 5.0 g dw | UAE+SPE | 10 ml of the mixture (Ethylacetate:formic acid; 50:1, v/v) | 3 | 55.8-108.9 | 0.3-1.7 | Kumirska et al. (2022) |
| TCs, SAs, FQs, MLs (5) | 1.0 g dw | UAE+SPE | 30 ml of mixture (acetonitrile:0.2M citric acid buffer - pH 4.4; 1:1, v/v) | 3 | 84.36-124 | 0.21-1.39 | [Pan](https://doi.org/10.1021/jf503850v) et al.  (2014) |
| TCs (4) | 1.0 g dw | MAE+μSPE | 10 ml of methanol | 1 | 71-110 | 0.1-6.3 | Jiao et al.  (2014) |
| FQs, NSAIDs, Beta-blockers, Antiepileptics, Diuretics, Lipid regulator (18) | 10.0 g dw | QuEChERS | 5 ml of water + 10 mL of 0.5% formic acid in acetonitrile | 1 | 0-99 | 0.05-0.5 | [Garcia](https://doi.org/10.1016/j.scitotenv.2021.146759) et al.  (2021) |
| FQs, TCs, SAs, MLs, LAs, PMs (24) | 5.0 g dw | UAE+SPE | 15 mL of extraction buffer (ACN:EDTA–McIlvaine buffer pH 4.0; 1:1, v/v);  2^nd^ extraction with 10 mL of 0.2 M NaOH | 2 | 53.8-95.8 | 0.01-2.0 | Bian et al.  (2015) |
| SAs, FQs, MLs, Inophore antibiotic, Cephalosporin, etc. (10) | 10.0 g dw | QuEChERS | 5 mL H_2_O + 10 mL Acetonitrile + 0.1 mL Acetic acid | 1 | 60.2-95.4 | 0.015-3.0 | Lee et al.  (2016) |

**Table S3.** Overview of extraction methods for parent pharmaceuticals in lettuce and vegetable samples (FQs – Fluoroquinolones, TCs – Tetracyclines, SAs – Sulfonamides, MLs – Macrolides, NSAIDs – Non-Steroidal Anti-Inflammatory Drugs, LAs – Lincosamides, ASE – Accelerated Solvent Extraction, SPE – Solid Phase Extraction, MASE – Microwave-Assisted Solvent Extraction, USE – Ultrasonic solvent extraction)

| **Analytes (No. of compounds)** | **Sample Weight** | **Extraction**  **method** | **Extraction Solvent** | **No. of extractions** | **RR [%]** | **LoD^[[2]](#footnote-2)^**  **[ng∙g^-1^]** | **Reference** |
| --- | --- | --- | --- | --- | --- | --- | --- |
| Analgesics, Anticonvulsants, SAs, LAs, TCs, MLs (11) | 0.5 g dw Lettuce | ASE+SPE | mixture (acetonitrile:methanol:water; 72:8:20, v/v/v) | 2 | 70.2-118.9 | 1.9-15.8 | Chuang et al.  (2015) |
| Analgesics, Anticonvulsants, SAs, LAs, TCs, MLs (11) | 0.5 g dw Lettuce | QuEChERS | 7 mL of mixture (acetonitrile:methanol:EDTA solution; 46.4:25.0:28.6, v:v:v) | 1 | 72.3-96.5 | 0.7-8.0 | Chuang et al.  (2015) |
| LAs, TCs, SAs, FQs (4) | 2.0 g dw  Lettuce | MASE+SPE | 10 mL of mixture (isopropyl alcohol:0.04-M citric acid solution; 80:20, v/v) | 1 | 55-93 | 40-105 | [Sallach](https://doi.org/10.1002/etc.3214) et al.  (2015) |
| Anticonvulsants, NSAIDs, SAs, Receptor Blockers, etc. (13) | 0.5 g dw  Lettuce | UAE+SPE | 1^st^ extraction 2 mL of water + 16 mL of a mixture (ACN:MeOH; 1:1, v/v)  2^nd^ extraction without water addition | 2 | 58-105 | 0.1-12.6 | Montemurro et al. (2017) |
| SAs, MLs, β-lactams, TCs (49) | 10.0 g  Cabbage | QuEChERS | 10 mL of acetonitrile | 1 | 46.5-103 | 2-5 | He et al.  (2018) |
| TCs, FQs, SAs (20) | 10.0 g  Vegetable | QuEChERS | 10 mL of mixture (acetonitrile:methanol; 85:15, v/v) | 1 | 58-92 | 0.41-2.92 | Yu et al.  (2017) |
| TCs, FQs, SAs (3) | 10.0 g  Radish leaf | QuEChERS | 20 mL of 1% acetic acid in acetonitrile | 1 | 100-111.3 | 0.6-6 | Chung et al.  (2017) |
| SAs, FQs, TCs, MLs (28) | 0.2 g dw  Leafy Vegetable | QuEChERS | 5 mL of citric acid buffer (pH 3) + 10 mL of 1.0% acetic acid in acetonitrile | 1 | 70.7-125 | 0.06-2.50 | Chen et al.  (2020) |

**Table S4.** Overview of extraction methods for parent pharmaceuticals in earthworms (FQs – Fluoroquinolones, SAs – Sulfonamides, MLs – Macrolides, NSAIDs – Non-Steroidal Anti-Inflammatory Drugs, SPE – Solid Phase Extraction)

| **Analytes (No. of compounds)** | **Sample Weight** | **Extraction**  **method** | **Extraction Solvent** | **No. of extractions** | **RR [%]** | **LoD^[[3]](#footnote-3)^**  **[ng∙g^-1^]** | **Reference** |
| --- | --- | --- | --- | --- | --- | --- | --- |
| SAs, Antiparasitics, MLs, β-Lactams, Hormones, Analgesics, NSAIDs (31) | Fresh weight  0.25 g | QuEChERS | 10 mL of acetonitrile + 6 mL of water | 1 | 45.2-105 | <14.0 | Bergé et al.  (2015) |
| Analgesics, Lipid regulators, FQs, MLs, Diuretics, Beta blocker, SAs, Anti-hypertensives, etc. (34) | Fresh weight  0.5 g | Vortexing+SPE | 8 mL of EDTA-McIlvaine buffer + 10 mL of acetonitrile | 1 | 14-130 | 0.01-0.54 | [Montemurro](https://doi.org/10.1016/j.chemosphere.2020.128222) et al. (2021) |

**Table S5.** Physico-chemical properties of pharmaceuticals (values from pubchem, N.D. not determined)

| **Pharmaceutical group** | **Substance name** | **Chemical formula** | **Mw [-]** | **pKa** | **log P** | **Solubility in water [mg∙L^-1^]** |
| --- | --- | --- | --- | --- | --- | --- |
| [Aantiepileptics](https://www.whocc.no/atc_ddd_index/?code=N03A&showdescription=no) | Carbamazepin | C_15_H_12_N_2_O | 236.3 | 13.9 | 2.5 | 35.4 |
| Beta blockers | Acebutolol | C_18_H_28_N_2_O_4_ | 336.4 | 9.5 | 1.7 | 259 |
|  | Atenolol | C_14_H_22_N_2_O_3_ | 266.3 | 9.6 | 0.2 | 40 |
|  | Nadolol | C_17_H_27_NO_4_ | 309.4 | 9.7 | 0.7 | 46.4 |
|  | Pindolol | C_14_H_20_N_2_O_2_ | 248.3 | 9.3 | 1.8 | 37.2 |
|  | Propranolol | C_16_H_21_NO_2_ | 259.3 | 9.5 | 3 | 61.7 |
| Fluoroquinolone antibacterials | Ciprofloxacin | C_17_H_18_FN_3_O_3_ | 331.3 | 6.1; 8.7 | -1.1 | <1 |
|  | Enrofloxacin | C_19_H_22_FN_3_O_3_ | 359.4 | N.D. | -0.2 | 53.9 |
|  | Moxifloxacin | C_21_H_24_FN_3_O_4_ | 401.4 | 6.3; 9.1 | 0.6 | 1,146 |
|  | Norfloxacin | C_16_H_18_FN_3_O_3_ | 319.3 | 6.1; 8.8 | -1 | 250,000 |
|  | Ofloxacin | C_18_H_20_FN_3_O_4_ | 361.4 | 6.0; 9.3 | -0.4 | 28,300 |
|  | Pefloxacin | C_17_H_20_FN_3_O_3_ | 333.4 | N.D. | 0.3 | 11,400 |
| Macrolides | Azithromycin | C_38_H_72_N_2_O_12_ | 749.0 | 8.5 | 4 | 2.37 |
|  | Clarithromycin | C_38_H_69_NO_13_ | 748.0 | 9.0 | 3.2 | 0.33 |
|  | Erythromycin | C_37_H_67_NO_13_ | 733.9 | 8.9 | 2.7 | 4.2 |
|  | Roxithromycin | C_41_H_76_N_2_O_15_ | 837.0 | 9.3 | 1.7 | 0.0189 |
| Nonsteroidal anti-inflammatory drugs | Celecoxib | C_17_H_14_F_3_N_3_O_2_S | 381.4 | 11.1 | 3.4 | 4.3 |
|  | Ketoprofen | C_16_H_14_O_3_ | 254.3 | 4.0 | 3.1 | 51 |
|  | Mefenamic acid | C_15_H_15_NO_2_ | 241.3 | 4.2 | 5.1 | 36.2 |
|  | Meloxicam | C_14_H_13_N_3_O_4_S_2_ | 351.4 | 4.4; 7.8 | 3 | 48.7 |
|  | Naproxen | C_14_H_14_O_3_ | 230.3 | 4.2 | 3.3 | 15.9 |
|  | Piroxicam | C_15_H_13_N_3_O_4_S | 331.3 | 6.3 | 3.1 | 49.7 |
| Sulfonamides  and Trimethoprim | Sulfacetamide | C_8_H_10_N_2_O_3_S | 214.2 | N.D. | -1 | 32.1 |
|  | Sulfadiazine | C_10_H_10_N_4_O_2_S | 250.3 | 6.4 | -0.1 | 77 |
|  | Sulfadimethoxine | C_12_H_14_N_4_O_4_S | 310.3 | N.D. | 1.6 | 343 |
|  | Sulfamerazine | C_11_H_12_N_4_O_2_S | 264.3 | N.D. | 0.1 | 202 |
|  | Sulfamethazine | C_12_H_14_N_4_O_2_S | 278.3 | 2.7; 7.7 | 0.3 | 1,500 |
|  | Sulfamethoxazole | C_10_H_11_N_3_O_3_S | 253.3 | 1.6; 5.7 | 0.9 | 610 |
|  | Sulfamethoxypyridazine | C_11_H_12_N_4_O_3_S | 280.3 | N.D. | 0.3 | N.D. |
|  | Sulfapyridine | C_11_H_11_N_3_O_2_S | 249.3 | 8.4 | 0 | 33.1 |
|  | Sulfasalazine | C_18_H_14_N_4_O_5_S | 398.4 | 2.3; 6.5 | -0.7 | Insoluble |
|  | Sulfathiazole | C_9_H_9_N_3_O_2_S_2_ | 255.3 | 2.2; 7.2 | 0.1 | 373 |
|  | Trimethoprim | C_14_H_18_N_4_O_3_ | 290.3 | 7.1 | 0.9 | 400 |
| Tetracyclines | Chlortetracycline | C_22_H_23_ClN_2_O_8_ | 478.9 | 7.4 | -0.62 | N.D. |
|  | Doxycycline | C_22_H_24_N_2_O_8_ | 444.4 | 3.1 | -0.7 | 50 |
|  | Minocycline | C_23_H_27_N_3_O_7_ | 457.5 | 2.8; 5.0; 7.8; 9.3 | -0.6 | 52,000 |
|  | Oxytetracycline | C_22_H_24_N_2_O_9_ | 460.4 | 3.3; 9.5 | -1.6 | 47 |
|  | Tetracycline | C_22_H_24_N_2_O_8_ | 444.4 | 3.3; 7.7 | -2 | 231 |
| Throat Preparations | Flurbiprofen | C_15_H_13_FO_2_ | 244.3 | 4.0 | 4.2 | 8 |
| Topical products for joint and muscular pain | Diclofenac | C_14_H_11_Cl_2_NO_2_ | 296.1 | 4.0 | 4.4 | 2.37 |
|  | Indomethacin | C_19_H_16_ClNO_4_ | 357.8 | 4.5 | 4.3 | 0.938 |
|  | Nimesulide | C_13_H_12_N_2_O_5_S | 308.3 | N.D. | 2.6 | N.D. |

**Table S6.** Physico-chemical properties of soil

| **Sampling location** | |
| --- | --- |
| State | Czech Republic |
| Region | South Moravian Region |
| City | Brno |
| Sampling depth [cm] | 0-25 |
| **Physico-chemical properties of soil** | |
| Soil texture | Silt Loam |
| Soil type | Fluvisol |
| Sand [%] | 18.3 |
| Silt [%] | 59.55 |
| Clay [%] | 22.15 |
| pH _(CaCl2)_ [-] | 5.97 |
| pH _(H2O)_ [-] | 6.65 |
| EC [mS∙cm^-1^] | 0.385 |
| Exchangable Mg^2+^ [mg∙kg-1] | 22.37 |
| Exchangable Ca^2+^ [mg∙kg^-1^] | 165.95 |
| Exchangable K^+^ [mg∙kg^-1^] | 4.12 |
| Exchangable Na^+^ [mg∙kg^-1^] | 1.54 |
| Total phosphorus [mg∙kg^-1^] | 93.3 |
| Total Nitrogen [%] | 0.8 |
| Organic carbon [%] | 1.5 |
| Organic matter [%] | 2.6 |
| As - Aqua regia [mg∙kg^-1^] | 10.1 |
| Al - Aqua regia [mg∙kg^-1^] | 25,007 |
| Be - Aqua regia [mg∙kg^-1^] | 1.2 |
| Ca - Aqua regia [mg∙kg^-1^] | 4,792 |
| Cd - Aqua regia [mg∙kg^-1^] | 0.3 |
| Co - Aqua regia [mg∙kg^-1^] | 14.3 |
| Cr - Aqua regia [mg∙kg^-1^] | 52.0 |
| Cu - Aqua regia [mg∙kg^-1^] | 25.3 |
| Fe - Aqua regia [mg∙kg^-1^] | 30,186 |
| K - Aqua regia [mg∙kg^-1^] | 4,609 |
| Mg - Aqua regia [mg∙kg^-1^] | 6,616 |
| Mn - Aqua regia [mg∙kg^-1^] | 844 |
| Mo - Aqua regia [mg∙kg^-1^] | 0.7 |
| Ni - Aqua regia [mg∙kg^-1^] | 35.8 |
| P - Aqua regia [mg∙kg^-1^] | 845 |
| Pb - Aqua regia [mg∙kg^-1^] | 25.4 |
| S - Aqua regia [mg∙kg^-1^] | 286 |
| V - Aqua regia [mg∙kg^-1^] | 51.3 |
| Zn - Aqua regia [mg∙kg^-1^] | 87.4 |

**
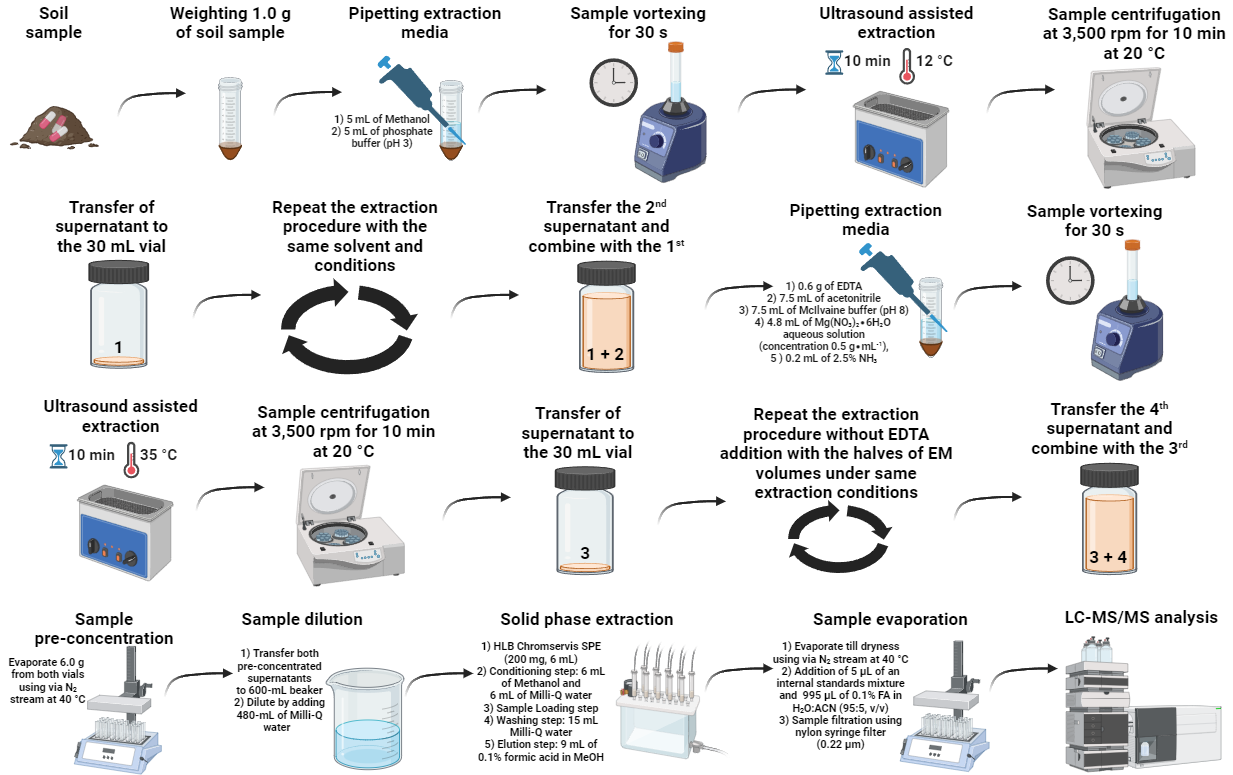
**

**Fig. S1** Extraction scheme for extraction of pharmaceuticals from soil samples
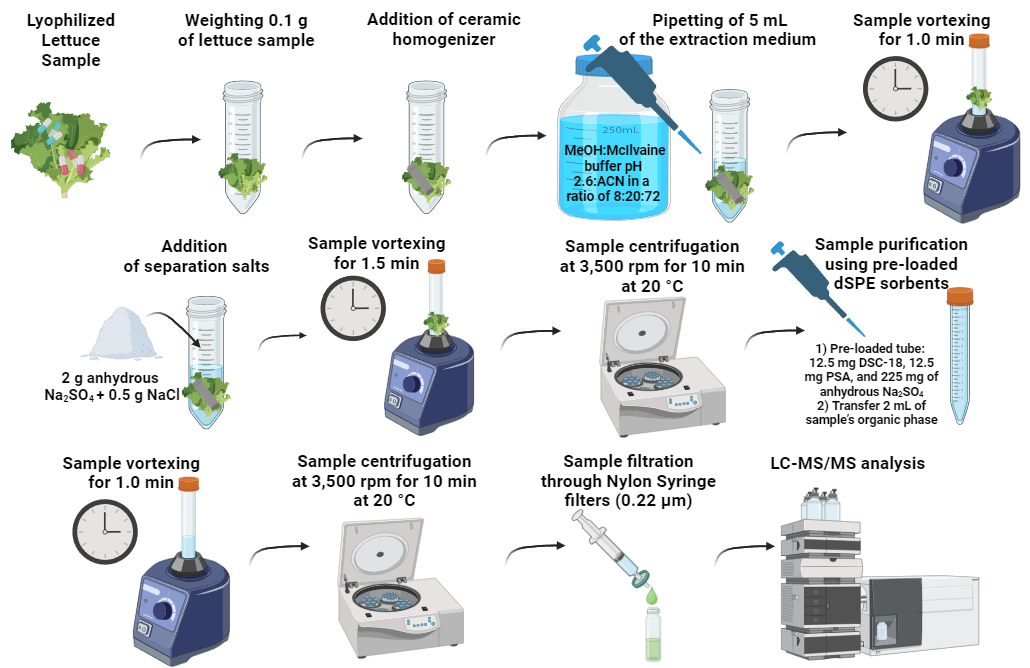


**Fig. S2** QuEChERS extraction scheme for extraction of pharmaceuticals from lettuce samples
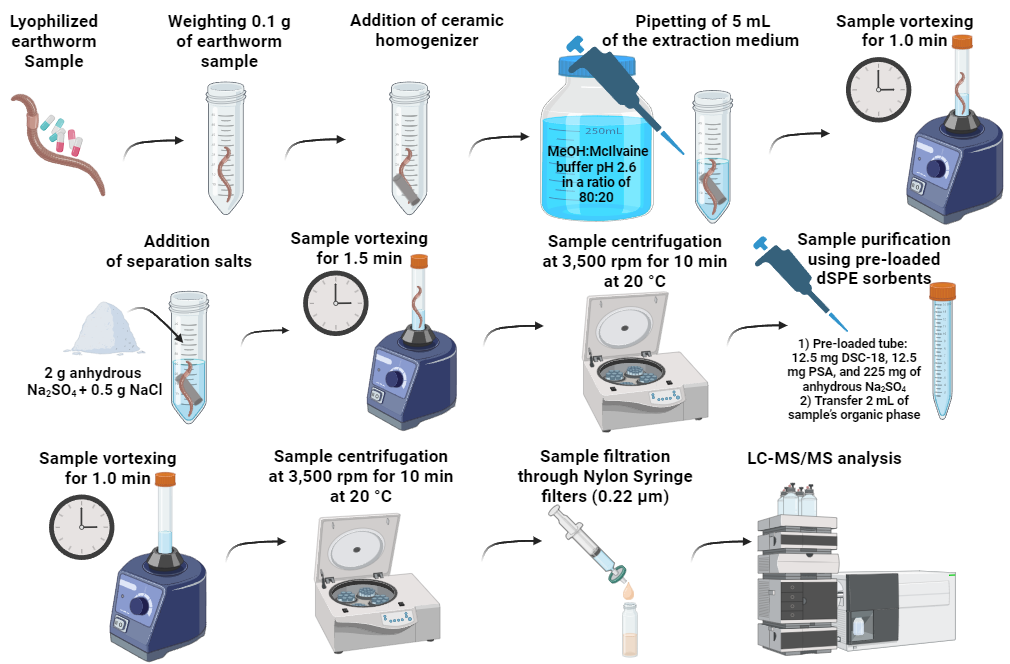


**Fig. S3** QuEChERS extraction scheme for extraction of pharmaceuticals from earthworm samples

**Table S7.** MRM transitions of selected pharmaceuticals

| **Analyte name** | **RT**  **[min]** | **Quantitative transition** | | | **Quantitative transition** | | | **Internal standards** |
| --- | --- | --- | --- | --- | --- | --- | --- | --- |
|  |  | **Precursor [m/z]** | **Productn[m/z]** | **CE [eV]** | **Precursor [m/z]** | **Productn[m/z]** | **CE [eV]** |  |
| Acebutolol | 3.65 | 337.2 | 116.0 | 20.0 | 337.2 | 319.0 | 10.0 | Atenolol-d7 |
| Atenolol | 1.17 | 267.0 | 145.2 | 20.0 | 267.0 | 190.1 | 10.0 | Atenolol-d7 |
| Atenolol-d7 | 1.17 | 274.0 | 145.0 | 20.0 | 274.0 | 190.0 | 10.0 | - |
| Azithromycin | 4.84 | 375.2 | 591.3 | 10.0 | 375.2 | 158.0 | 20.0 | Spiramycin |
| Carbamazepine | 6.68 | 237.1 | 194.1 | 20.0 | 237.1 | 179.0 | 10.0 | none |
| Celecoxib | 10.99 | 382.2 | 361.8 | 20.0 | 382.2 | 301.1 | 30.0 | none |
| Chlortetracycline | 4.39 | 479.0 | 462.3 | 10.0 | 479.0 | 443.7 | 20.0 | none |
| Ciprofloxacin | 3.35 | 332.4 | 314.2 | 10.0 | 332.4 | 288.2 | 10.0 | Ciprofloxacin-d8 |
| Ciprofloxacin-d8 | 3.35 | 340.4 | 322.1 | 15.0 | 340.4 | 296.1 | 15.0 | - |
| Clarithromycin | 7.54 | 748.5 | 158.1 | 20.0 | 748.5 | 590.4 | 10.0 | Spiramycin |
| Diclofenac | 10.25 | 296.0 | 214.9 | 10.0 | 296.0 | 249.9 | 10.0 | none |
| Doxycycline | 5.00 | 445.4 | 428.1 | 10.0 | 445.4 | 320.9 | 30.0 | none |
| Enrofloxacin | 3.78 | 360.4 | 316.2 | 10.0 | 360.4 | 342.2 | 20.0 | Enrofloxacin-d5 |
| Enrofloxacin-d5 | 3.78 | 365.4 | 321.0 | 10.0 | 365.4 | 346.8 | 20.0 | - |
| Erythromycin | 6.57 | 734.5 | 158.2 | 30.0 | 734.5 | 576.3 | 10.0 | Spiramycin |
| Flurbiprofen | 9.85 | 243.3 | 198.8 | 10.0 | 198.8 | 198.8 | 20.0 | none |
| Indomethacin | 10.39 | 357.7 | 139.1 | 10.0 | 357.7 | 111.0 | 40.0 | none |
| Ketoprofen | 8.34 | 255.0 | 209.0 | 10.0 | 255.0 | 105.0 | 20.0 | none |
| Mefenamic acid | 11.19 | 240.0 | 180.0 | 20.0 | 240.0 | 196.0 | 10.0 | none |
| Meloxicam | 8.63 | 352.0 | 115.0 | 10.0 | 352.0 | 141.0 | 20.0 | none |
| Minocycline | 2.35 | 458.5 | 337.0 | 30.0 | 458.5 | 441.3 | 15.0 | none |
| Moxifloxacin | 4.83 | 402.0 | 358.2 | 20.0 | 402.0 | 384.2 | 20.0 | Enrofloxacin-d5 |
| Nadolol | 2.50 | 310.2 | 253.8 | 10.0 | 310.2 | 200.8 | 20.0 | Atenolol-d7 |
| Naproxen | 8.39 | 231.0 | 185.2 | 10.0 | 231.0 | 169.0 | 20.0 | none |
| Nimesulide | 9.22 | 307.3 | 229.1 | 10.0 | 307.3 | 198.0 | 20.0 | none |
| Norfloxacin | 3.22 | 320.1 | 276.1 | 10.0 | 320.1 | 231.1 | 40.0 | Ciprofloxacin-d8 |
| Ofloxacin | 3.25 | 362.2 | 318.1 | 10.0 | 362.2 | 261.1 | 20.0 | Enrofloxacin-d5 |
| Oxytetracycline | 2.85 | 461.0 | 426.2 | 20.0 | 461.0 | 443.2 | 5.0 | none |
| Pefloxacin | 3.34 | 334.0 | 317.0 | 10.0 | 334.0 | 289.0 | 10.0 | Enrofloxacin-d5 |
| Pindolol | 2.59 | 249.2 | 116.22 | 10.0 | 249.2 | 172.2 | 10.0 | Atenolol-d7 |
| Piroxicam | 6.78 | 332.0 | 95.0 | 20.0 | 332.0 | 121.0 | 20.0 | none |
| Propranolol | 5.42 | 259.3 | 116.0 | 15.0 | 259.3 | 183.0 | 15.0 | none |
| Roxithromycin | 8.03 | 837.5 | 679.5 | 20.0 | 837.5 | 157.9 | 30.0 | Spiramycin |
| Spiramycin | 4.78 | 422.8 | 699.5 | 5.0 | 422.8 | 540.5 | 5.0 | - |
| Sulfacetamide | 1.62 | 215.2 | 156.0 | 5.0 | 215.2 | 108.2 | 10.0 | Sulfamethoxazole-d4 |
| Sulfadiazine | 1.96 | 251.3 | 156.0 | 10.0 | 251.3 | 92.2 | 20.0 | Sulfamethoxazole-d4 |
| Sulfadimethoxine | 5.48 | 255.0 | 156.0 | 10.0 | 255.0 | 92.1 | 15.0 | Sulfamethoxazole-d4 |
| Sulfamerazine | 2.51 | 265.3 | 156.0 | 10.0 | 265.3 | 172.0 | 10.0 | Sulfamethoxazole-d4 |
| Sulfamethazine | 3.02 | 279.3 | 186.0 | 10.0 | 279.3 | 92.0 | 30.0 | Sulfamethoxazole-d4 |
| Sulfamethoxazole | 4.14 | 254.3 | 156.0 | 10.0 | 254.3 | 108.1 | 20.0 | Sulfamethoxazole-d4 |
| Sulfamethoxazole-d4 | 4.12 | 258.3 | 159.8 | 10.0 | 258.3 | 96.2 | 20.0 | - |
| Sulfamethoxypyridazine | 3.20 | 281.1 | 156.0 | 10.0 | 281.1 | 92.2 | 20.0 | Sulfamethoxazole-d4 |
| Sulfapyridine | 1.97 | 250.3 | 156.0 | 10.0 | 250.3 | 92.2 | 20.0 | Sulfamethoxazole-d4 |
| Sulfasalazine | 7.11 | 399.3 | 381.0 | 10.0 | 399.3 | 223.0 | 20.0 | Sulfamethoxazole-d4 |
| Sulfathiazole | 2.27 | 256.0 | 156.0 | 10.0 | 256.0 | 92.2 | 20.0 | Sulfathiazole-d4 |
| Sulfathiazole-d4 | 2.23 | 260.0 | 96.0 | 20.0 | 260.0 | 160.0 | 10.0 | - |
| Tetracycline | 5.00 | 445.0 | 427.2 | 10.0 | 445.0 | 428.0 | 10.0 | none |
| Trimethoprim | 2.68 | 291.2 | 230.1 | 20.0 | 291.2 | 261.0 | 20.0 | Trimethoprim-d9 |
| Trimethoprim-d9 | 2.68 | 300.1 | 123.0 | 20.0 | 300.1 | 233.8 | 20.0 | - |

**Section 2. Description of the Exposure Experiments**

**Earthworms (*Eisenia fetida*)**

Earthworms were purchased from a local fish store called ProRyby (Czech Republic). The uptake experiment adhered to the Organisation for Economic Cooperation and Development (OECD) guidelines. Preceding the beginning of the experiment, the earthworms underwent a 3-day acclimatization period in uncontaminated soil. This phase was succeeded by a 1-day depuration period on filtration paper in the dark. The soil (physicochemical properties of soil in Table S6) was spiked with a mixture of 27 pharmaceuticals (Beta Blockers – Acetobutolol, Nadolol; FQs – Ciprofloxacin, Enrofloxacin, Moxifloxacin, Norfloxacin, Ofloxacin, Pefloxacin; MLs - Azithromycin, Clarithromycin, Erythromycin, Roxithromycin; NSAIDs – Ketoprofen; SAs - Sulfacetamide, Sulfadiazine, Sulfadimethoxine, Sulfamerazine, Sulfamethazine, Sulfamethoxazole, Sulfamethoxypyridazine, Sulfapyridine, Sulfathiazole, Trimethoprim; TCs - Doxycycline, Chlortetracycline, Oxytetracycline, Tetracycline) at a concentration of 1,000 ng∙g^-1^ dw of soil. Subsequently, 50 ± 0.1 g of dry soil was carefully measured into each 100 mL beaker, and one earthworm with average weight of 400 mg per beaker was transferred into the soil. To facilitate proper aeration, the beakers were covered with perforated food plastic wrap. Soil humidity was adjusted to 40% MWHC, meticulously checked, and adjusted every third day. Throughout the 24-h light period, the experiment maintained a temperature of 20 ± 1 °C. Earthworms were provided with flakes on the first day of the experiment and subsequently every third day. Sampling occurred after 21 days of exposure, with the depuration phase lasting 1 day. After depuration, the earthworms were lyophilized and extracted using the QuEChERS method. Soil sampling occurred at both the start and end of the experiment.

**Lettuce (*Lactuca sativa*)**

This study was conducted within a controlled environment using a grow box (Green-Qube 1020L) with a 16-h photoperiod (17,500 lux; LED panel: ViparSpectra XS2000 230W), maintaining an air temperature of 23±1°C and air humidity of 45±5%. To ensure proper air exchange and flow, a single extraction fan and two oscillating fans were strategically placed within the grow box. The soil was spiked with a mixture of 27 pharmaceuticals at a concentration of 1,000 ng∙g^-1^ dry weight. Each PET pot (diameter of 95 mm; height of 80 mm; without drainage) was filled with 500±1 g dry weight of soil (physicochemical properties of soil in Table S6). Subsequently, single pre-grown lettuces (5 days old) on cotton wool were planted in each pot, and five replicates of each experiment were established. Initially, dry soil was watered with tap water up to 40% maximum water holding capacity (MWHC) and was consistently watered daily to maintain constant soil humidity throughout the uptake experiments. Weekly applications of a water-soluble organic liquid fertilizer (Natura, Czech Republic) with N-P-K values of 6.4–1.7-9.0 were employed as recommended. The pots were arranged randomly within the grow box, with pot positions altered every third day to counterbalance variations in light intensity. Lettuces were sampled after 28 days of exposure, encompassing both control and contaminated samples. Samples of leaves and roots obtained were washed in deionized water to remove pharmaceuticals from the plant surface. Soil sampling occurred at the start and end of the experiment.**Table S8.** Overview of different EM used for extraction of pharmaceuticals from soil

| **Number**  **of Extraction Media** | **Composition of Extraction Media** |
| --- | --- |
| EM 1 | 5 ml ACN + 5 ml Milli-Q water |
| EM 2 | 5 ml MeOH + 5 ml Milli-Q water |
| EM 3 | 5 ml ACN + 5 ml phosphate buffer pH 3.0 (KH_2_PO_4_+H_3_PO_4_) |
| EM 4 | 5 ml MeOH + 5 ml phosphate buffer pH 3.0 (KH_2_PO_4_+H_3_PO_4_) |
| EM 5 | 5 ml ACN + 5 ml McIlvaine buffer pH 2.6 |
| EM 6 | 5 ml ACN + 5 ml McIlvaine buffer pH 4.0 |
| EM 7 | 5 ml ACN + 5 ml McIlvaine buffer pH 8.0 |
| EM 8 | 0.6 g EDTA + 2.5 ml ACN + 2.5 ml MeOH + 5 ml McIlvaine buffer pH 2.6 |
| EM 9 | 5 ml MeOH + 5 ml McIlvaine buffer pH 2.6 |
| EM 10 | 5 ml MeOH + 5 ml McIlvaine buffer pH 4.0 |
| EM 11 | 5 ml MeOH + 5 ml McIlvaine buffer pH 8.0 |
| EM 12 | 0.6 g EDTA + 7.5 ml ACN + 7.5 ml McIlvaine buffer pH 2.6 + 0.2 ml 2.5% NH_3_  4.8 ml Mg(NO_3_)_2_∙6H_2_O (C=0.5 g∙mL^-1^); second extraction with halves of volumes |
| EM 13 | 0.6 g EDTA + 7.5 ml ACN + 7.5 ml McIlvaine buffer pH 4.0 + 0.2 ml 2.5% NH_3_  4.8 ml Mg(NO_3_)_2_∙6H_2_O (C=0.5 g∙mL^-1^); second extraction with halves of volumes |
| EM 14 | 0.6 g EDTA + 7.5 ml ACN + 7.5 ml McIlvaine buffer pH 6.0 + 0.2 ml 2.5% NH_3_  4.8 ml Mg(NO_3_)_2_∙6H_2_O (C=0.5 g∙mL^-1^); second extraction with halves of volumes |
| EM 15 | 0.6 g EDTA + 7.5 ml ACN + 7.5 ml McIlvaine buffer pH 8.0 + 0.2 ml 2.5% NH_3_  4.8 ml Mg(NO_3_)_2_∙6H_2_O (C=0.5 g∙mL^-1^); second extraction with halves of volumes |

**Section 3. Preparation of the Buffer Solution for the Extraction Media**

**Phosphate buffer pH 3.0**

To prepare Phosphate buffer at pH 3.0, 27.2 g of potassium dihydrogen phosphate was weighed using analytical balances. This weight was quantitatively transferred to a 1,000 mL volumetric flask and dissolved in Milli-Q water. Subsequently, 1.35 mL of 85 % phosphoric acid was added to the volumetric flask, which was subsequently filled to the mark with Milli-Q water. The pH value of the buffer was verified using a pH meter and was determined to be 3.0 ± 0.1.

**McIlvaine buffer pH 2.6**

To prepare McIlvaine's buffer at pH 2.6, 3.91 g of sodium dodecahydrate and 9.36 g of citric acid were weighed using analytical balances. These weights were then quantitatively transferred to a 500 mL volumetric flask, which was subsequently filled to the mark with Milli-Q water. The pH value of the buffer was verified using a pH meter and was determined to be 2.6 ± 0.1.

**McIlvaine buffer pH 4.0**

To prepare McIlvaine's buffer at pH 4.0, 13.82 g of sodium dodecahydrate and 6.46 g of citric acid were weighed using analytical balances. These weights were then quantitatively transferred to a 500 mL volumetric flask, which was subsequently filled to the mark with Milli-Q water. The pH value of the buffer was verified using a pH meter and was determined to be 4.0 ± 0.1.

**McIlvaine buffer pH 6.0**

To prepare McIlvaine's buffer at pH 6.0, 23.66 g of sodium dodecahydrate and 3.56 g of citric acid were weighed using analytical balances. These weights were then quantitatively transferred to a 500 mL volumetric flask, which was subsequently filled to the mark with Milli-Q water. The pH value of the buffer was verified using a pH meter and was determined to be 6.0 ± 0.1.

**McIlvaine buffer pH 7.0**

To prepare McIlvaine's buffer at pH 7.0, 29.52 g of sodium dodecahydrate and 1.86 g of citric acid were weighed using analytical balances. These weights were then quantitatively transferred to a 500 mL volumetric flask, which was subsequently filled to the mark with Milli-Q water. The pH value of the buffer was verified using a pH meter and was determined to be 7.0 ± 0.1.

**McIlvaine buffer pH 8.0**

To prepare McIlvaine’s buffer at pH 8.0, 34.82 g of sodium dodecahydrate and 0.28 g of citric acid were weighed using analytical balances. These weights were then quantitatively transferred to a 500 mL volumetric flask, which was subsequently filled to the mark with Milli-Q water. The pH value of the buffer was verified using a pH meter and was determined to be 8.0 ± 0.1.


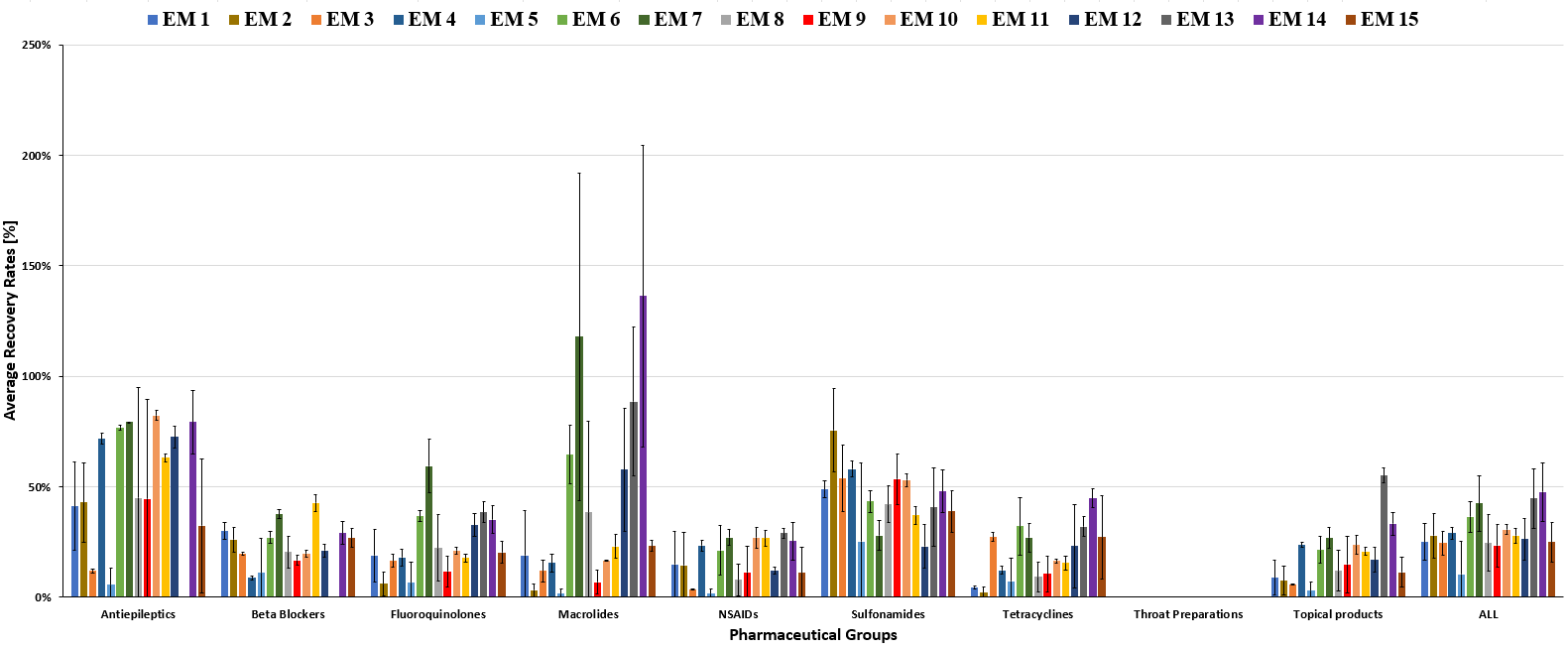


**Fig. S4** Method optimization for extraction of PhACs from soil using EM1-EM15


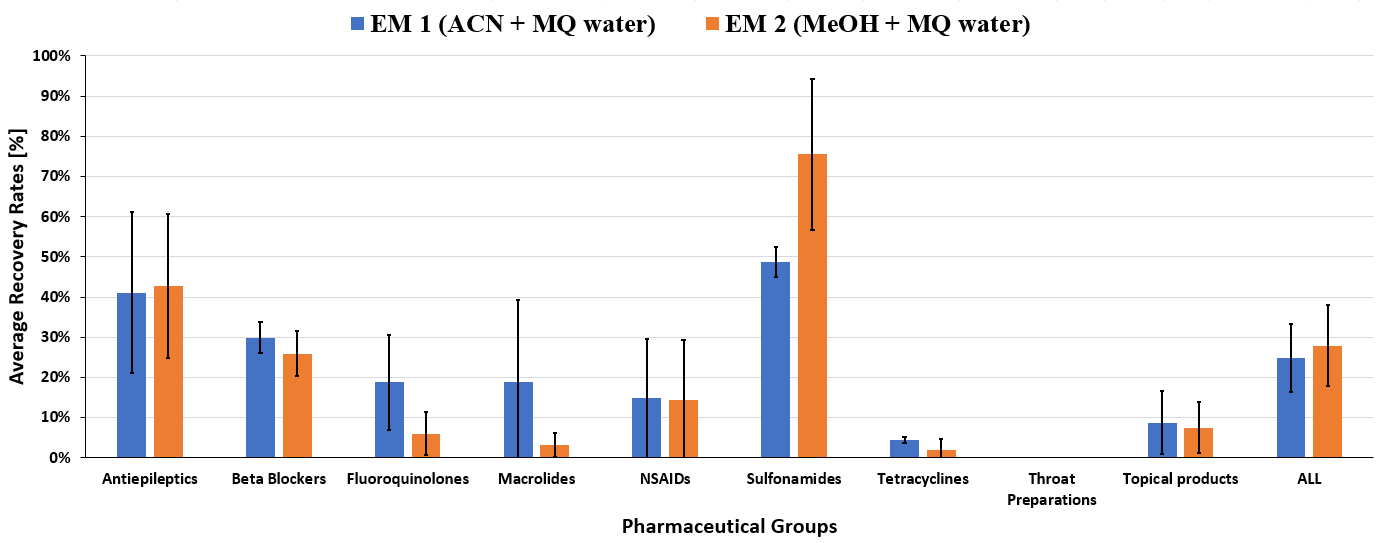


**Fig. S5** Method optimization for extraction of PhACs from soil (influence of organic solvent)


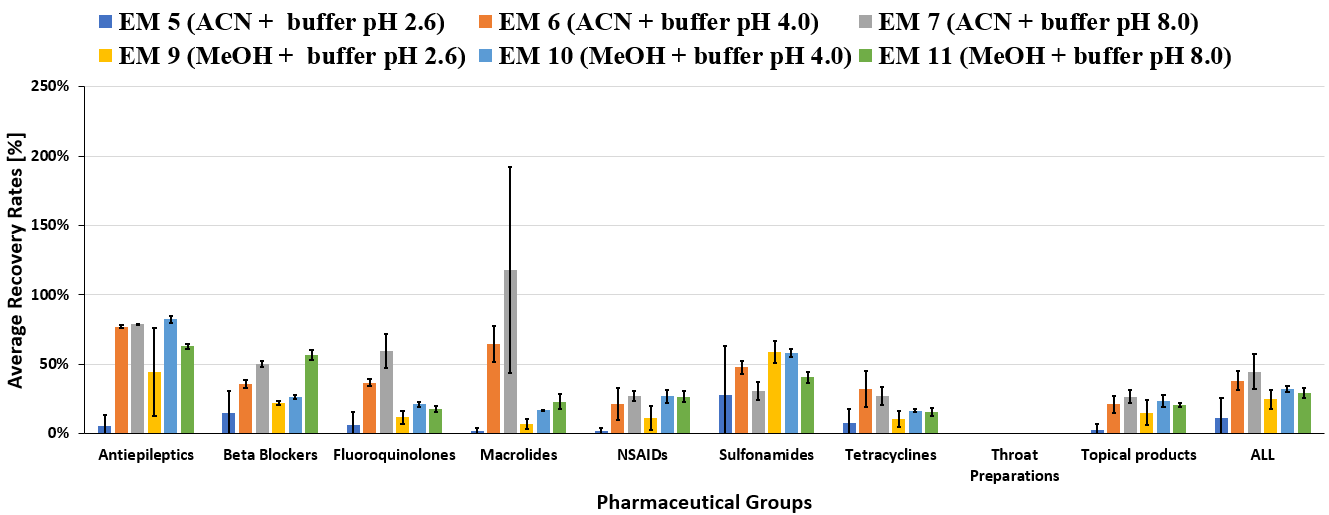


**Fig. S6** Method optimization for extraction of PhACs from soil (infuence of pH)


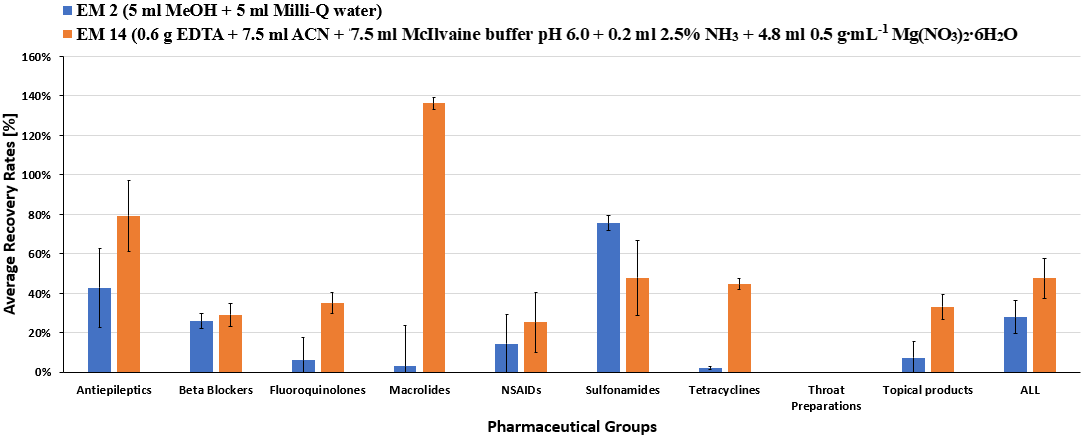


**Fig. S7** Method optimization for extraction of PhACs from soil (infuence of extraction mechanism)


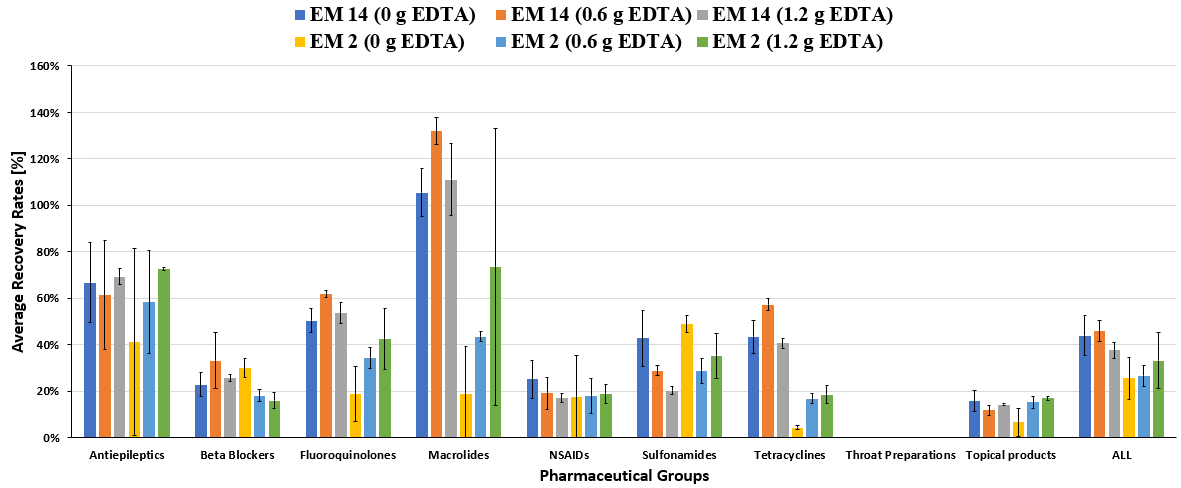


**Fig. S8** Method optimization for extraction of PhACs from soil (infuence of EDTA)


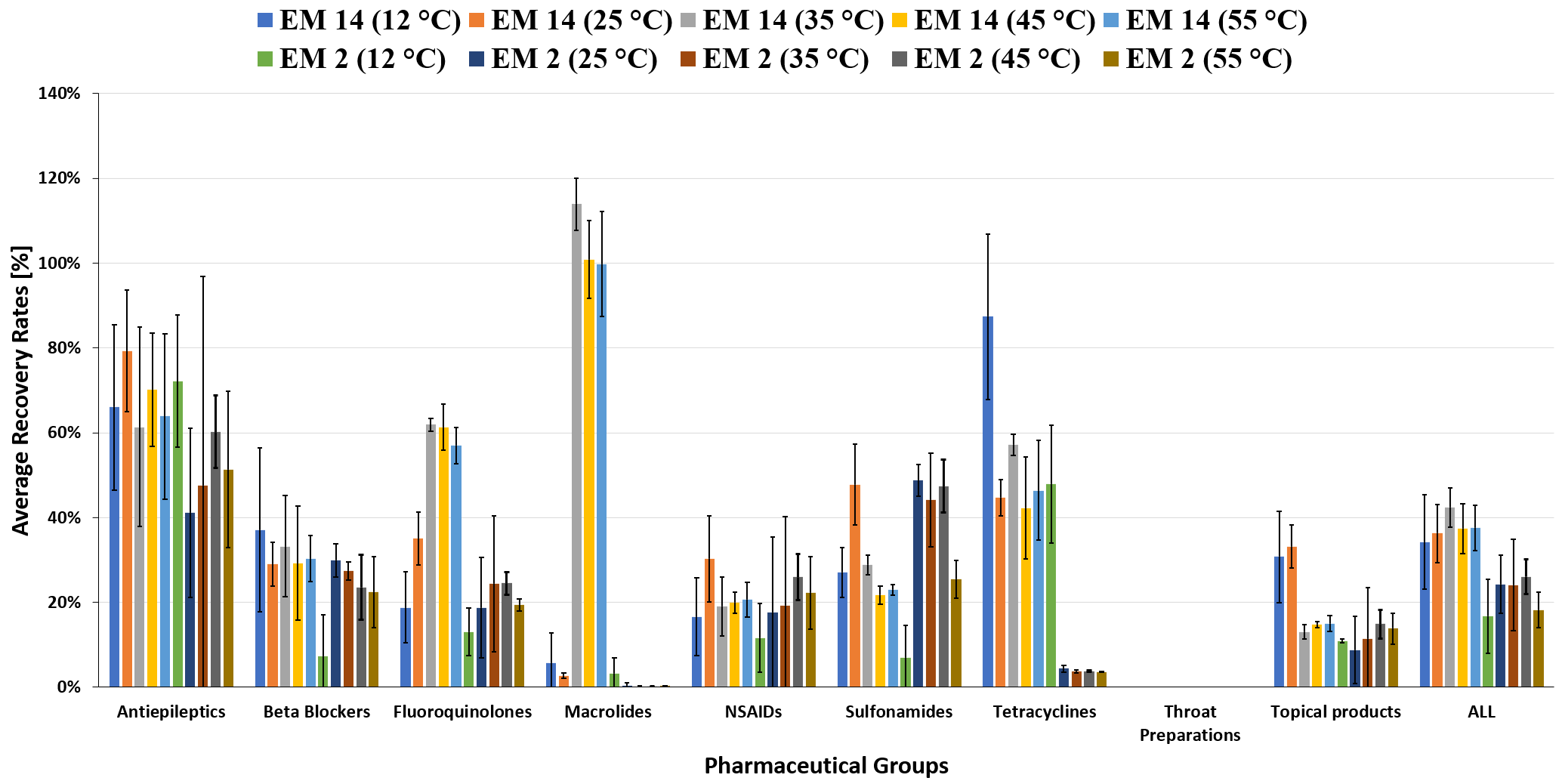


**Fig. S9** Method optimization for extraction of PhACs from soil (infuence of extraction temperature)


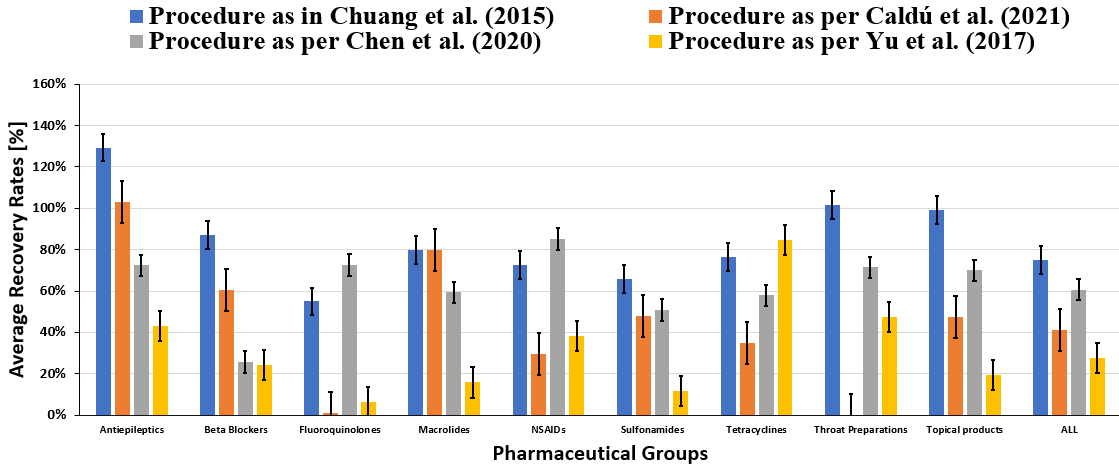


**Fig. S10** Method optimization for extraction of PhACs from lettuce leaves (different EM)


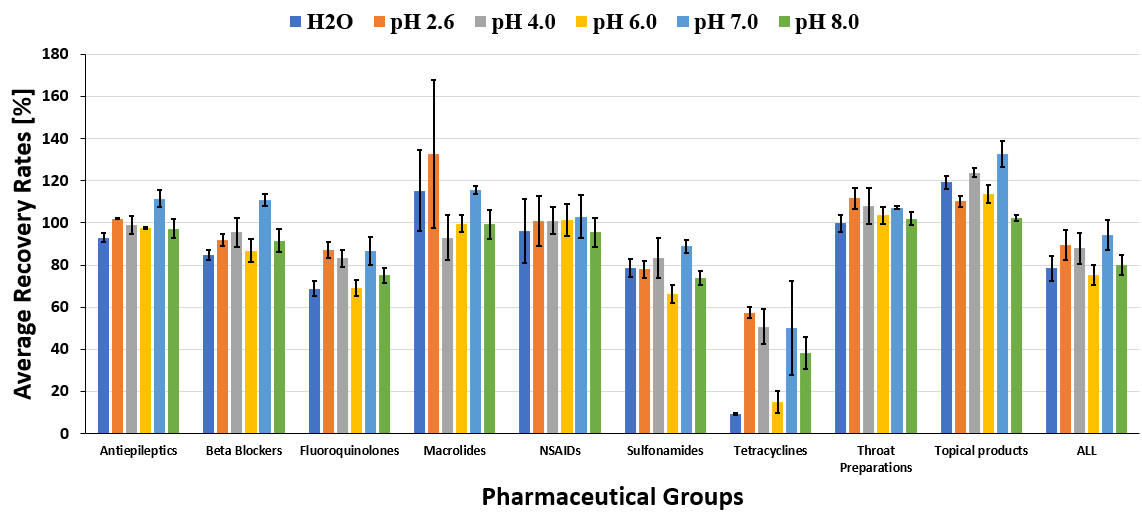


**Fig. S11** Method optimization for extraction of PhACs from lettuce leaves (influence of pH)


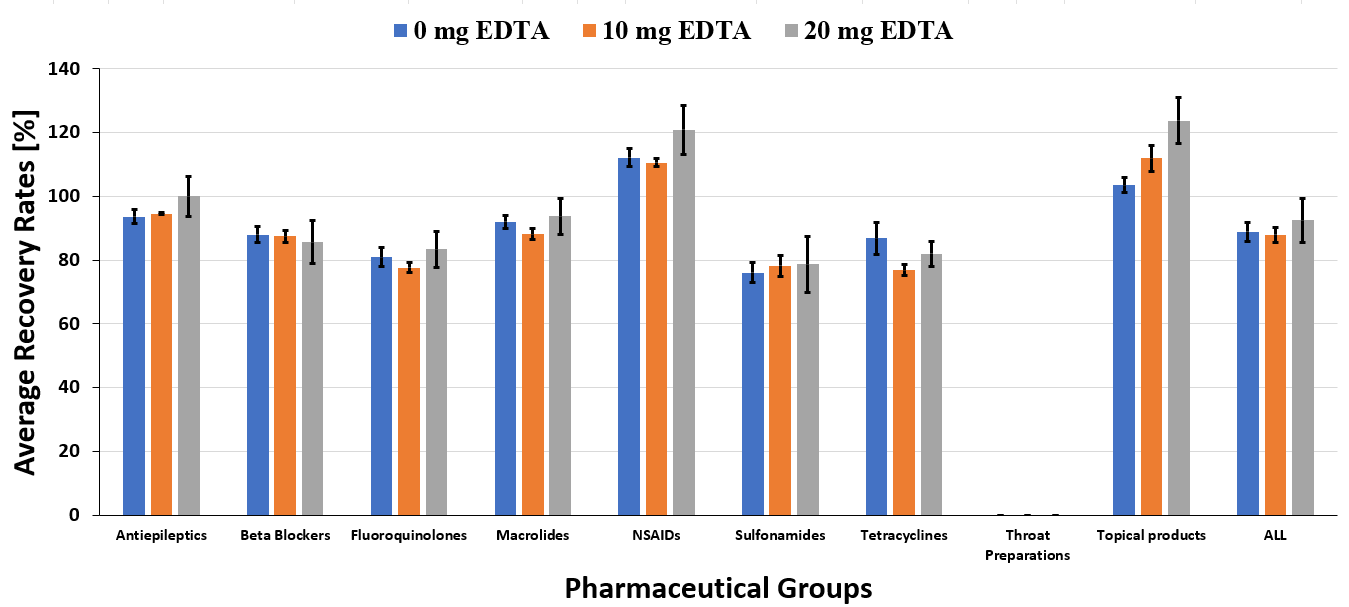


**Fig. S12** Method optimization for extraction of PhACs from lettuce leaves (influence of EDTA)


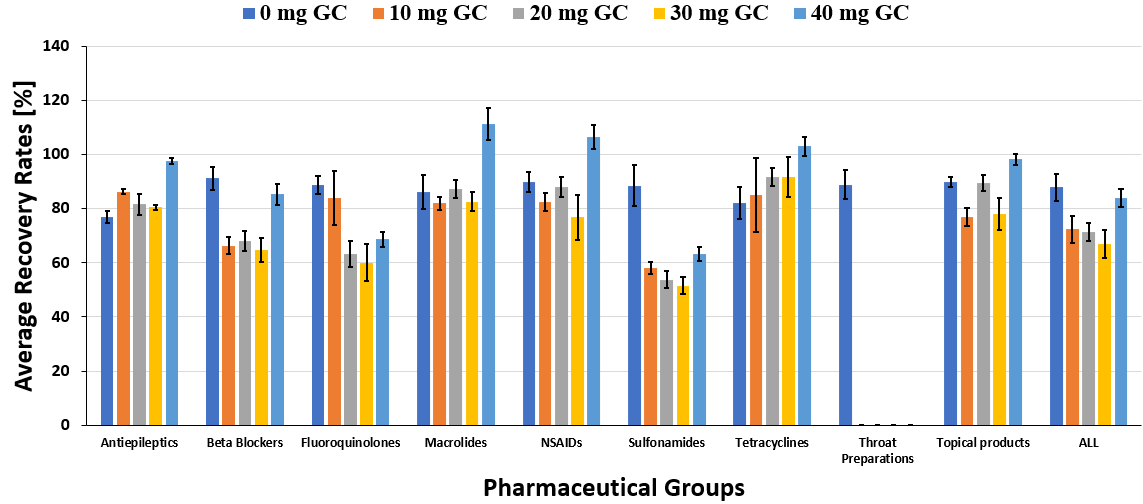


**Fig. S13** Method optimization for extraction of PhACs from lettuce leaves (influence of dSPE sorbents)


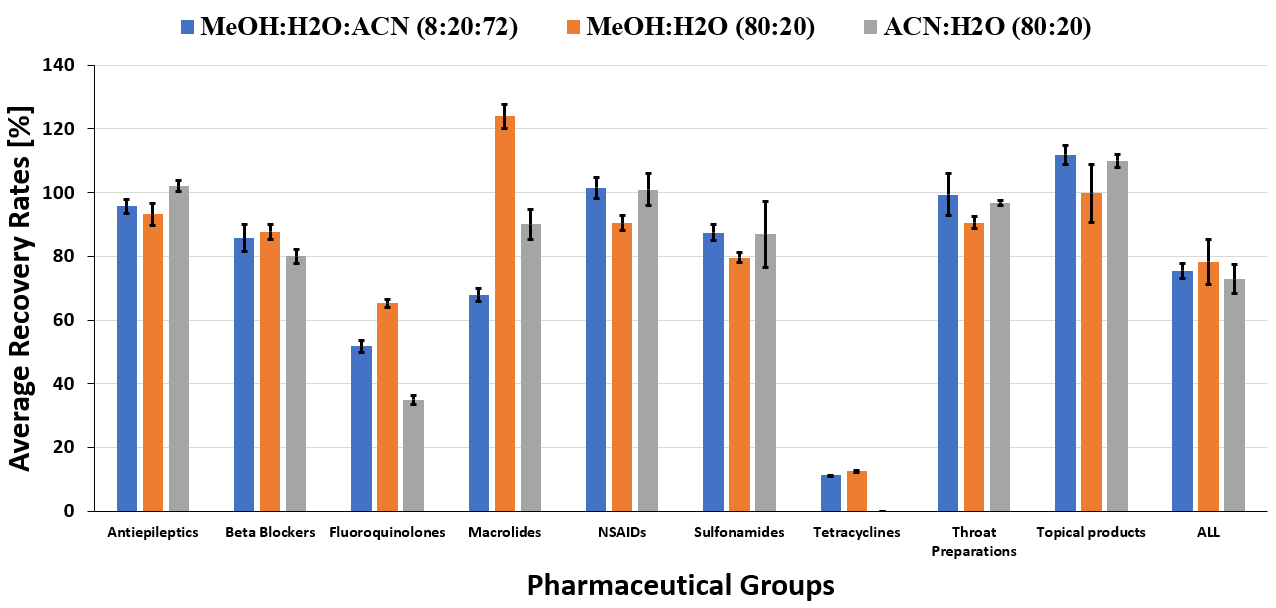


**Fig. S14** Method optimization for extraction of PhACs from earthworms (influence of organic solvents)


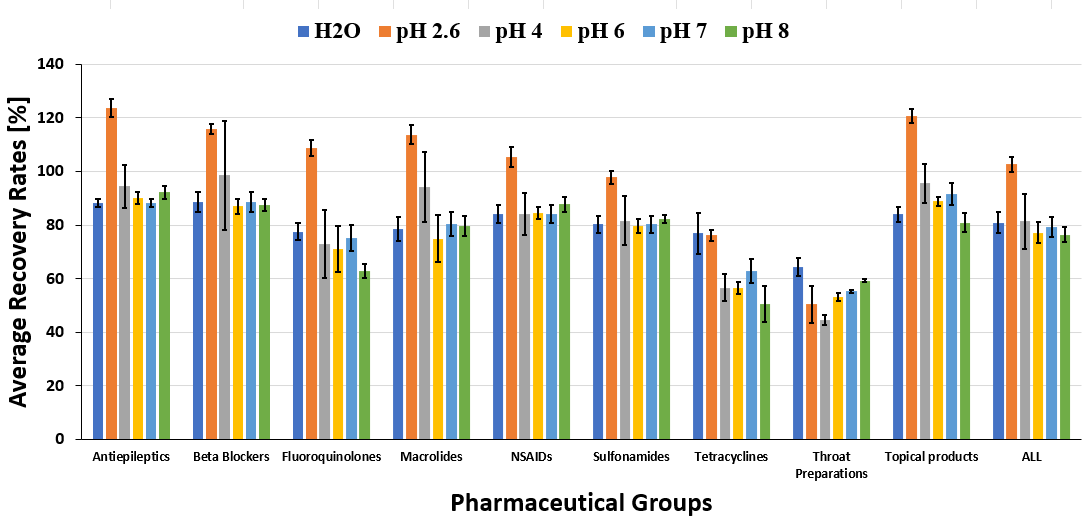


**Fig. S15** Method optimization for extraction of PhACs from earthworms (influence of pH)


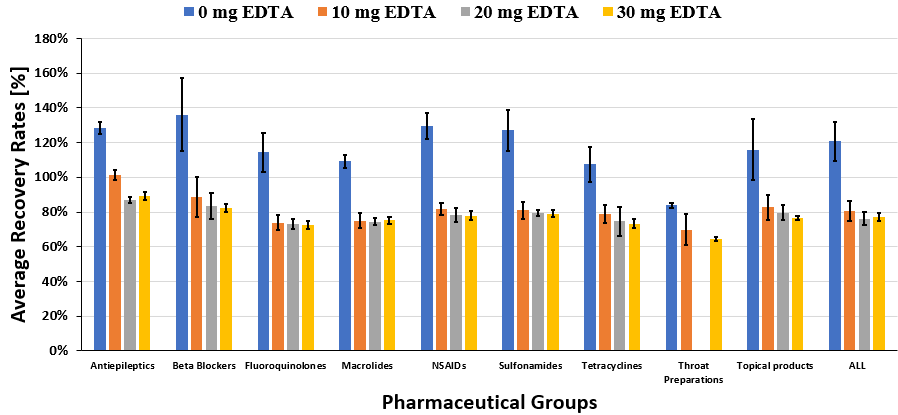


**Fig. S16** Method optimization for extraction of PhACs from earthworms (influence of EDTA)


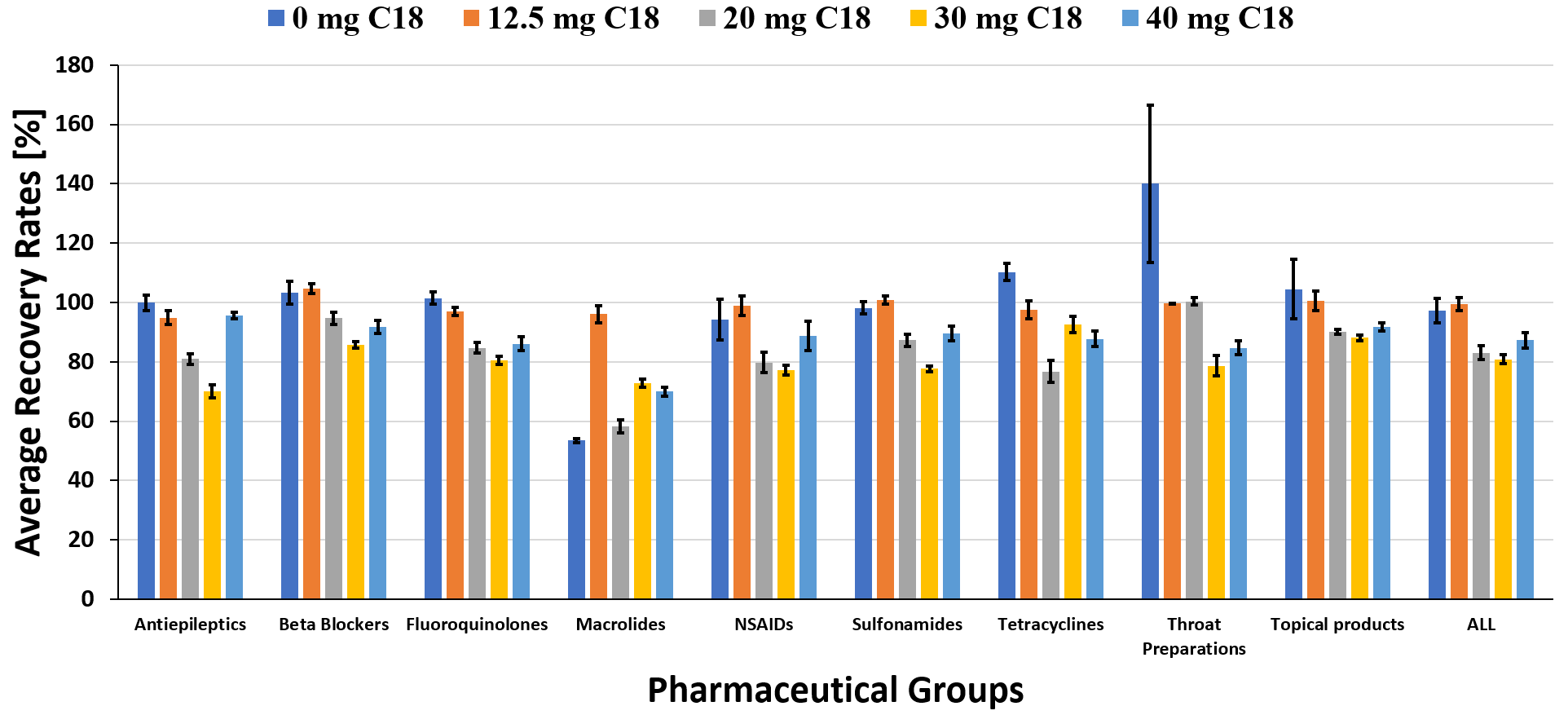


**Fig. S17** Method optimization for extraction of PhACs from earthworms (influence of dSPE sorbents)

**Table S9.** Pharmaceutical concentration in Earthworms (N=11) after 21 days of exposure at soil concentration 1,000 ng∙g^-1^

| **Pharmaceutical**  **group** | **Analyte Name** | **Detection**  **Frequency**  **Above**  **LoD [%]** | **Average**  **Concentration**  **in Earthworms**  **[ng∙g^-1^]** | **The Lowest**  **Detected**  **Concentration**  **[ng∙g^-1^]** | **The Highest**  **Detected**  **Concentration**  **[ng∙g^-1^]** | **Degradation**  **in Soil [%]** | **RQ quotients after 21 days** | |
| --- | --- | --- | --- | --- | --- | --- | --- | --- |
|  |  |  |  |  |  |  | **MIN value [-]** | **MAX value [-]** |
| Beta blocker | Acetobutolol | 73 | 327 | 53 | 1,791 | 29 | NA | NA |
|  | Nadolol | 55 | 56 | 23 | 90 | 97 | NA | NA |
| Fluoroquinolone antibacterials | Ciprofloxacin | 27 | 79 | 58 | 110 | 38 | 26.0 | 26.2 |
|  | Enrofloxacin | 9 | 39 | - | - | 14 | 2.0 | 48 |
|  | Moxifloxacin | 36 | 91 | 33 | 153 | 61 | 1.7 | |
|  | Norfloxacin | 9 | 297 | - | - | 30 | 0.2 | 2.0 |
|  | Ofloxacin | 36 | 534 | 56 | 839 | 24 | 0.3 | 4.0 |
|  | Pefloxacin | 18 | 86 | 28 | 144 | 9 | N.D. | N.D. |
| Macrolides | Azithromycin | 18 | 777 | 122 | 1,432 | 31 | 12 | 108 |
|  | Clarithromycin | 64 | 1,123 | 45 | 3,918 | 23 | 381 | 1,600 |
|  | Erythromycin | 9 | 2,559 | - | - | 56 | 3.0 | 15 |
|  | Roxithromycin | 45 | 482 | 55 | 2,016 | 63 | 0.4 | 1.5 |
| Nonsteroidal anti-inflammatory drugs | Ketoprofen | 55 | 1,185 | 48 | 3,449 | 27 | NA | NA |
| Sulfonamides  and Trimethoprim | Sulfacetamide | 27 | 76 | 43 | 115 | 94 | N.D. | N.D. |
|  | Sulfadiazine | 18 | 148 | 62 | 233 | 95 | N.D. | N.D. |
|  | Sulfadimethoxine | 18 | 215 | 138 | 291 | 85 | N.D. | N.D. |
|  | Sulfamerazine | 64 | 12,327 | 908 | 52,588 | 90 | N.D. | N.D. |
|  | Sulfamethazine | 27 | 51 | 31 | 73 | 80 | N.D. | N.D. |
|  | Sulfamethoxazole | 18 | 107 | 61 | 152 | 90 | 0.1 | 0.6 |
|  | Sulfamethoxypyridazine | 45 | 193 | 62 | 481 | 84 | N.D. | N.D. |
|  | Sulfapyridine | 45 | 91 | 35 | 239 | 89 | N.D. | N.D. |
|  | Sulfathiazole | 45 | 53 | 19 | 147 | 93 | N.D. | N.D. |
|  | Trimethoprim | 55 | 7,244 | 65 | 23,439 | 30 | 6.5 | 68 |
| Tetracyclines | Chlortetracycline | 9 | 140 | - | - | 46 | N.D. | N.D. |
|  | Doxycycline | 64 | 17,066 | 74 | 76,175 | 28 | 0.0 | 0.4 |
|  | Oxytetracycline | 9 | 94 | - | - | 0 | 0.7 | 1.7 |
|  | Tetracycline | 36 | 233 | 124 | 424 | 19 | 0.2 | 1.8 |

**Table S10.** Pharmaceutical concentration in Lettuce Roots (N=5) after 28 days of exposure at soil concentration 1,000 ng∙g^-1^

| **Pharmaceutical**  **group** | **Analyte Name** | **Detection**  **Frequency**  **Above**  **LoD [%]** | **Average**  **Concentration**  **in Earthworms**  **[ng∙g^-1^]** | **The Lowest**  **Detected**  **Concentration**  **[ng∙g^-1^]** | **The Highest**  **Detected**  **Concentration**  **[ng∙g^-1^]** | **Degradation**  **in Soil [%]** | **RQ quotients after 21 days** | |
| --- | --- | --- | --- | --- | --- | --- | --- | --- |
|  |  |  |  |  |  |  | **MIN value [-]** | **MAX value [-]** |
| Beta blocker | Acetobutolol | 100 | 309 | 61 | 456 | 56 | NA | NA |
|  | Nadolol | 0 | - | - | - | 99 | NA | NA |
| Fluoroquinolone antibacterials | Ciprofloxacin | 80 | 147 | 71 | 205 | 33 | 23.9 | 24.1 |
|  | Enrofloxacin | 100 | 233 | 48 | 351 | 25 | 2.3 | 55 |
|  | Moxifloxacin | 40 | 434 | 371 | 497 | 32 | 1.0 | |
|  | Norfloxacin | 80 | 157 | 112 | 211 | 40 | 0.2 | 2.4 |
|  | Ofloxacin | 80 | 304 | 179 | 386 | 38 | 0.4 | 5.0 |
|  | Pefloxacin | 60 | 321 | 304 | 333 | 34 | N.D. | N.D. |
| Macrolides | Azithromycin | 80 | 159 | 71 | 216 | 0 | 8.0 | 75 |
|  | Clarithromycin | 100 | 93 | 16 | 181 | 0 | 292 | 1,224 |
|  | Erythromycin | 60 | 447 | 211 | 567 | 0 | 1.3 | 6.5 |
|  | Roxithromycin | 80 | 545 | 107 | 912 | 0 | 0.2 | 0.6 |
| Nonsteroidal anti-inflammatory drugs | Ketoprofen | 20 | 17 | - | - | 99 | NA | NA |
| Sulfonamides  and Trimethoprim | Sulfacetamide | 0 | - | - | - | 98 | N.D. | N.D. |
|  | Sulfadiazine | 0 | - | - | - | 96 | N.D. | N.D. |
|  | Sulfadimethoxine | 80 | 163 | 121 | 246 | 91 | N.D. | N.D. |
|  | Sulfamerazine | 0 | - | - | - | 92 | N.D. | N.D. |
|  | Sulfamethazine | 0 | - | - | - | 92 | N.D. | N.D. |
|  | Sulfamethoxazole | 100 | 169 | 37 | 327 | 92 | 0.1 | 0.8 |
|  | Sulfamethoxypyridazine | 40 | 48 | 38 | 57 | 94 | N.D. | N.D. |
|  | Sulfapyridine | 0 | - | - | - | 93 | N.D. | N.D. |
|  | Sulfathiazole | 0 | - | - | - | 97 | N.D. | N.D. |
|  | Trimethoprim | 60 | 122 | 59 | 236 | 66 | 13 | 140 |
| Tetracyclines | Chlortetracycline | 40 | 53 | 42 | 64 | 77 | N.D. | N.D. |
|  | Doxycycline | 40 | 89 | 70 | 108 | 67 | 0.0 | 0.8 |
|  | Oxytetracycline | 40 | 92 | 26 | 157 | 64 | 1.9 | 4.8 |
|  | Tetracycline | 80 | 67 | 52 | 108 | 65 | 0.5 | 4.1 |

**Table S11.** Pharmaceutical concentration in Lettuce Leaves (N=5) after 28 days of exposure at soil concentration 1,000 ng∙g^-1^

| **Pharmaceutical**  **group** | **Analyte Name** | **Detection**  **Frequency**  **Above**  **LoD [%]** | **Average**  **Concentration**  **in Earthworms**  **[ng∙g^-1^]** | **The Lowest**  **Detected**  **Concentration**  **[ng∙g^-1^]** | **The Highest**  **Detected**  **Concentration**  **[ng∙g^-1^]** |
| --- | --- | --- | --- | --- | --- |
| Beta blocker | Acetobutolol | 40 | 41 | 26 | 56 |
|  | Nadolol | 0 | - | - | - |
| Fluoroquinolone antibacterials | Ciprofloxacin | 40 | 94 | 79 | 109 |
|  | Enrofloxacin | 40 | 101 | 79 | 124 |
|  | Moxifloxacin | 0 | - | - | - |
|  | Norfloxacin | 0 | - | - | - |
|  | Ofloxacin | 0 | - | - | - |
|  | Pefloxacin | 0 | - | - | - |
| Macrolides | Azithromycin | 0 | - | - | - |
|  | Clarithromycin | 0 | - | - | - |
|  | Erythromycin | 0 | - | - | - |
|  | Roxithromycin | 20 | 104 | - | - |
| Nonsteroidal anti-inflammatory drugs | Ketoprofen | 0 | - | - | - |
| Sulfonamides  and Trimethoprim | Sulfacetamide | 0 | - | - | - |
|  | Sulfadiazine | 0 | - | - | - |
|  | Sulfadimethoxine | 0 | - | - | - |
|  | Sulfamerazine | 0 | - | - | - |
|  | Sulfamethazine | 40 | 55 | 53 | 57 |
|  | Sulfamethoxazole | 20 | 62 | - | - |
|  | Sulfamethoxypyridazine | 0 | - | - | - |
|  | Sulfapyridine | 0 | - | - | - |
|  | Sulfathiazole | 0 | - | - | - |
|  | Trimethoprim | 0 | - | - | - |
| Tetracyclines | Doxycycline | 0 | - | - | - |
|  | Chlortetracycline | 40 | 32 | 26 | 38 |
|  | Oxytetracycline | 20 | 71 | - | - |
|  | Tetracycline | 20 | 49 | - | - |

**REFERENCES**

AMR Alliance science-based PNEC targets for risk assessments (2023) Available at: <https://www.amrindustryalliance.org/wp-content/uploads/2023/02/AMR-Table-1-Update-20230222_corrected.pdf>

Bergé A, Vulliet E (2015) Development of a method for the analysis of hormones and pharmaceuticals in earthworms by quick, easy, cheap, effective, rugged and safe (QuEChERS) extraction followed by liquid chromatography-tandem mass spectrometry (LC-MS/MS). Anal Bioanal Chem 407:7995–8008. https://doi.org/10.1007/s00216-015-8972-z

Bian K, Liu Y, Wang Z, Zhou T, Song X, Zhang F, He L (2015) Determination of multi-class antimicrobial residues in soil by liquid chromatography-tandem mass spectrometry. RSC Adv. 5:27584–27593. https://doi.org/10.1039/C4RA13919D

Chen J, He L-X, Cheng Y-X, Ye P, Wu D-L, Fang Z-Q, Li J, Ying G-G (2020) Trace analysis of 28 antibiotics in plant tissues (root, stem, leaf and seed) by optimized QuEChERS pretreatment with UHPLC-MS/MS detection. Journal of Chromatography B 1161:122450. https://doi.org/10.1016/j.jchromb.2020.122450

Chuang Y-H, Zhang Y, Zhang W, Boyd SA, Li H (2015) Comparison of accelerated solvent extraction and quick, easy, cheap, effective, rugged and safe method for extraction and determination of pharmaceuticals in vegetables. Journal of Chromatography A 1404:1–9. https://doi.org/10.1016/j.chroma.2015.05.022

Chung HS, Lee Y-J, Rahman MdM, Abd El-Aty AM, Lee HS, Kabir MdH, Kim SW, Park B-J, Kim J-E, Hacımüftüoğlu F, Nahar N, Shin H-C, Shim J-H (2017) Uptake of the veterinary antibiotics chlortetracycline, enrofloxacin, and sulphathiazole from soil by radish. Science of The Total Environment 605–606:322–331. <https://doi.org/10.1016/j.scitotenv.2017.06.231>

WHO collective - Critically important antimicrobials for human medicine, 6th revision. Geneva: World Health Organization (2019).

Ferhi S, Bourdat-Deschamps M, Daudin J-J, Houot S, Nélieu S (2016) Factors influencing the extraction of pharmaceuticals from sewage sludge and soil: an experimental design approach. Anal Bioanal Chem 408:6153–6168. <https://doi.org/10.1007/s00216-016-9725-3>

Fučík J, Amrichová A, Brabcová K, Karpíšková R, Koláčková I, Pokludová L, Poláková Š, Mravcová L (2024) Fate of fluoroquinolones in field soil environment after incorporation of poultry litter from a farm with enrofloxacin administration via drinking water. In Environmental Science and Pollution Research (Vol. 31, Issue 13, pp. 20017–20032). https://doi.org/10.1007/s11356-024-32492-x

García Valverde M, Martínez Bueno MJ, Gómez-Ramos MM, Aguilera A, Gil García MD, Fernández-Alba AR (2021) Determination study of contaminants of emerging concern at trace levels in agricultural soil. A pilot study. Science of The Total Environment 782:146759. https://doi.org/10.1016/j.scitotenv.2021.146759

Golovko O, Koba O, Kodesova R, Fedorova G, Kumar V, Grabic R (2016) Development of fast and robust multiresidual LC-MS/MS method for determination of pharmaceuticals in soils. Environ Sci Pollut Res 23:14068–14077. <https://doi.org/10.1007/s11356-016-6487-6>

Gravesen C, Judy JD (2020) Effect of biosolids characteristics on retention and release behavior of azithromycin and ciprofloxacin. Environmental Research 184:109333. https://doi.org/10.1016/j.envres.2020.109333

Hang L, Zhao Y, Liu C, Yu Y, He Y, Xu J, Lu Z (2021) Determine Multiple Classes of Veterinary Antibiotics in Soil: Comparing Dispersive and Solid-Phase Extraction for Sample Cleanup. Chromatographia 84:833–844. <https://doi.org/10.1007/s10337-021-04064-5>

Harrower J, McNaughtan M, Hunter C, Hough R, Zhang Z, Helwig K (2021) Chemical Fate and Partitioning Behavior of Antibiotics in the Aquatic Environment—A Review. Enviro Toxic and Chemistry 40:3275–3298. [https://doi.org/10.1002/etc.5191](https://doi.org/10.1007/s10337-021-04064-5)

He Z, Wang Y, Xu Y, Liu X (2018) Determination of Antibiotics in Vegetables Using QuEChERS-Based Method and Liquid Chromatography-Quadrupole Linear Ion Trap Mass Spectrometry. Food Anal. Methods 11:2857–2864. https://doi.org/10.1007/s12161-018-1252-8

Huang Y, Cheng M, Li W, Wu L, Chen Y, Luo Y, Christie P, Zhang H (2013) Simultaneous extraction of four classes of antibiotics in soil, manure and sewage sludge and analysis by liquid chromatography-tandem mass spectrometry with the isotope-labelled internal standard method. Anal. Methods 5:3721. https://doi.org/10.1039/c3ay40220g

Jiao Z, Guo Z, Zhang S, Chen H (2014) Microwave-assisted micro-solid-phase extraction for analysis of tetracycline antibiotics in environmental samples. International Journal of Environmental Analytical Chemistry 95:82–91. https://doi.org/10.1080/03067319.2014.983497

Kumirska J, Łukaszewicz P, Caban M, Migowska N, Plenis A, Białk-Bielińska A, Czerwicka M, Qi F, Piotr S (2019) Determination of twenty pharmaceutical contaminants in soil using ultrasound-assisted extraction with gas chromatography-mass spectrometric detection. Chemosphere 232:232–242. https://doi.org/10.1016/j.chemosphere.2019.05.164

Lee Y, Choi J, Abd El‐Aty AM, Chung HS, Lee HS, Kim S, Rahman MdM, Park B, Kim J, Shin H, Shim J (2016) Development of a single‐run analytical method for the detection of ten multiclass emerging contaminants in agricultural soil using an acetate‐buffered QuEChERS method coupled with LC–MS/MS. J of Separation Science 40:415–423. https://doi.org/10.1002/jssc.201600953

Mastro F, Cocozza C, Traversa A, Cacace C, Mottola F, Mezzina A, Brunetti G (2022) Validation of a modified QuEChERS method for the extraction of multiple classes of pharmaceuticals from soils. Chem. Biol. Technol. Agric. 9. <https://doi.org/10.1186/s40538-022-00305-3>

Meng F, Sun S, Geng J, Ma L, Jiang J, Li B, Yabo SD, Lu L, Fu D, Shen J, Qi H (2023) Occurrence, distribution, and risk assessment of quinolone antibiotics in municipal sewage sludges throughout China. Journal of Hazardous Materials 453:131322. [https://doi.org/10.1016/j.jhazmat.2023.131322](https://doi.org/10.1186/s40538-022-00305-3)

Montemurro N, Joedicke J, Pérez S (2021) Development and application of a QuEChERS method with liquid chromatography-quadrupole time of flight-mass spectrometry for the determination of 50 wastewater-borne pollutants in earthworms exposed through treated wastewater. Chemosphere 263:128222. https://doi.org/10.1016/j.chemosphere.2020.128222

Montemurro N, Postigo C, Lonigro A, Perez S, Barceló D (2017) Development and validation of an analytical method based on liquid chromatography–tandem mass spectrometry detection for the simultaneous determination of 13 relevant wastewater-derived contaminants in lettuce. Anal Bioanal Chem 409:5375–5387. https://doi.org/10.1007/s00216-017-0363-1

Pan M, Wong CKC, Chu LM (2014) Distribution of Antibiotics in Wastewater-Irrigated Soils and Their Accumulation in Vegetable Crops in the Pearl River Delta, Southern China. J. Agric. Food Chem. 62:11062–11069. <https://doi.org/10.1021/jf503850v>

Pan M, Chu LM (2016) Adsorption and degradation of five selected antibiotics in agricultural soil. Science of The Total Environment 545–546:48–56. [https://doi.org/10.1016/j.scitotenv.2015.12.040](https://doi.org/10.1021/jf503850v)

Rodríguez-López L, Santás-Miguel V, Cela-Dablanca R, Núñez-Delgado A, Álvarez-Rodríguez E, Pérez-Rodríguez P, Arias-Estévez M (2022) Ciprofloxacin and Trimethoprim Adsorption/Desorption in Agricultural Soils. IJERPH 19:8426. [https://doi.org/10.3390/ijerph19148426](https://doi.org/10.1021/jf503850v)

Rodríguez-López L, Santás-Miguel V, Cela-Dablanca R, Núñez-Delgado A, Álvarez-Rodríguez E, Rodríguez-Seijo A, Arias-Estévez M (2023) Clarithromycin as soil and environmental pollutant: Adsorption-desorption processes and influence of pH. Environmental Research 233:116520. [https://doi.org/10.1016/j.envres.2023.116520](https://doi.org/10.1021/jf503850v)

Sallach JB, Snow D, Hodges L, Li X, Bartelt‐Hunt S (2015) Development and comparison of four methods for the extraction of antibiotics from a vegetative matrix. Enviro Toxic and Chemistry 35:889–897. <https://doi.org/10.1002/etc.3214>

Sanford JCC, Mackie RI, Koike S, Krapac IG, Lin Y, Yannarell AC, Maxwell S, Aminov RI (2009) Fate and Transport of Antibiotic Residues and Antibiotic Resistance Genes following Land Application of Manure Waste. J of Env Quality 38:1086–1108. [https://doi.org/10.2134/jeq2008.0128](https://doi.org/10.1002/etc.3214)

Silva JJ, Silva BF, Stradiotto NR, Petrovic M, Gago-Ferrero P, Gros M (2020) Pressurized Liquid Extraction (PLE) and QuEChERS evaluation for the analysis of antibiotics in agricultural soils. MethodsX 7:101171. <https://doi.org/10.1016/j.mex.2020.101171>

Tang J, Wang S, Fan J, Long S, Wang L, Tang C, Tam NF, Yang Y (2019) Predicting distribution coefficients for antibiotics in a river water–sediment using quantitative models based on their spatiotemporal variations. Science of The Total Environment 655:1301–1310. [https://doi.org/10.1016/j.scitotenv.2018.11.163](https://doi.org/10.1016/j.mex.2020.101171)

Tetzner NF, Maniero MG, Rodrigues-Silva C, Rath S (2016) On-line solid phase extraction-ultra high performance liquid chromatography-tandem mass spectrometry as a powerful technique for the determination of sulfonamide residues in soils. Journal of Chromatography A 1452:89–97. <https://doi.org/10.1016/j.chroma.2016.05.034>

Thiele‐Bruhn S (2003) Pharmaceutical antibiotic compounds in soils – a review. Z. Pflanzenernähr. Bodenk. 166:145–167. [https://doi.org/10.1002/jpln.200390023](https://doi.org/10.1016/j.chroma.2016.05.034)

Yu X, Liu H, Pu C, Chen J, Sun Y, Hu L (2017) Determination of multiple antibiotics in leafy vegetables using QuEChERS–UHPLC–MS/MS. J of Separation Science 41:713–722. https://doi.org/10.1002/jssc.201700798

Yu Z, Yediler A, Yang M, Schulte-Hostede S (2012) Leaching behavior of enrofloxacin in three different soils and the influence of a surfactant on its mobility. Journal of Environmental Sciences 24:435–439. https://doi.org/10.1016/S1001-0742(11)60771-7

1. The stated limit of detection (LoD) represents the method's LoD in ng∙g⁻¹ for soil samples. [↑](#footnote-ref-1)
2. The stated limit of detection (LoD) represents the method's LoD in ng∙g⁻¹ for vegetable samples. [↑](#footnote-ref-2)
3. The stated limit of detection (LoD) represents the method's LoD in ng∙g⁻¹ for earthworms samples. [↑](#footnote-ref-3)
